# Heterocycle Effects on the Liquid Crystallinity of Terthiophene Analogues

**DOI:** 10.3390/ma12142314

**Published:** 2019-07-19

**Authors:** David F. Ester, Declan McKearney, Khrystyna Herasymchuk, Vance E. Williams

**Affiliations:** Department of Chemistry, Simon Fraser University, 8888 University Drive, Burnaby, BC V5A 1S6, Canada

**Keywords:** organic semiconductors, self-assembly, lamellar structures, 2D charge transport pathways (or 2D lamellar arrays), structure-property relationships, liquid crystals

## Abstract

Liquid crystalline self-assembly offers the potential to create highly ordered, uniformly aligned, and defect-free thin-film organic semiconductors. Analogues of one of the more promising classes of liquid crystal semiconductors, 5,5”-dialkyl-α-terthiophenes, were prepared in order to investigate the effects of replacing the central thiophene with either an oxadiazole or a thiadiazole ring. The phase behaviour was examined by differential scanning calorimetry, polarized optical microscopy, and variable temperature x-ray diffraction. While the oxadiazole derivative was not liquid crystalline, thiadiazole derivatives formed smectic C and soft crystal lamellar phases, and maintained lamellar order down to room temperature. Variation of the terminal alkyl chains also influenced the observed phase sequence. Single crystal structures revealed the face-to-face orientation of molecules within the layers in the solid-state, a packing motif that is rationalized based on the shape and dipole of the thiadiazole ring, as corroborated by density functional theory (DFT) calculations. The solution opto-electronic properties of the systems were characterized by absorption and emission spectroscopy, cyclic voltammetry, and time-dependent density functional theory (TD-DFT).

## 1. Introduction

Organic semiconductors are attractive materials due to their light weight, mechanical flexibility, tunability through chemical modification, and compatibility with solution processing techniques that allow for low-cost device fabrication [1,2,3,4]. Although they have proven suitable for applications such as light emitting diodes (OLEDs) [5,6,7,8], field-effect transistors (OFETs) [9,10,11,12,13], and photovoltaics (OPVs) [14,15,16,17], an organic semiconductor’s performance is ultimately limited by its supramolecular ordering. Structural defects, poor alignment, and grain boundaries all degrade mobility [18,19,20,21]. Despite significant efforts to address these issues through chemical modification and sample processing [22,23,24,25,26,27,28,29,30] reliable approaches for optimizing supramolecular ordering for high performance organic semiconductors based on 3D crystalline materials remain elusive.

Liquid crystals (LCs) represent a promising alternative to common crystalline solid organic semiconductors, owing to their ability to self-assemble into ordered self-healing structures capable of uniform alignment over large areas, and with supramolecular order that can be maintained in the solid-state [31,32,33,34,35,36,37]. Although columnar phases have been the most commonly explored liquid crystalline semiconductors, their one dimensional charge conduction is highly susceptible to defects [38,39,40,41,42,43,44]. More recently, smectic LCs have emerged as more attractive candidate materials since their layered structure permits conduction in two dimensions, potentially leading to more consistent performance [45,46,47,48,49]. Simultaneously tuning both the opto-electronic properties and self-assembly of smectic LC materials represents an ongoing challenge in their exploitation as organic semiconductors.

**Figure 1:**
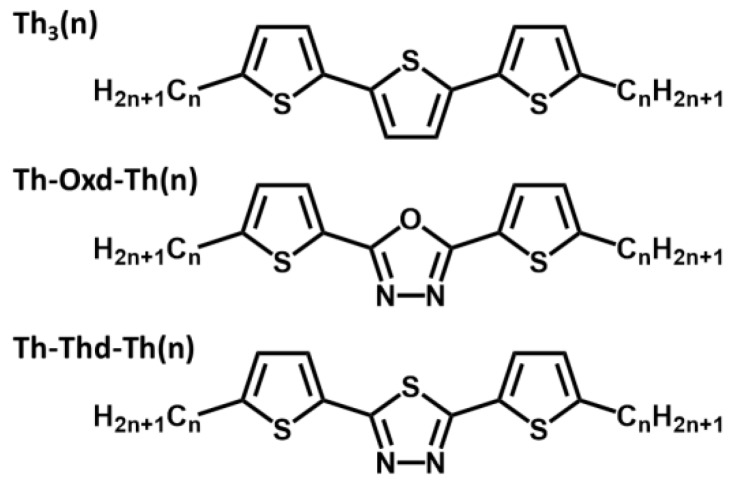
The structures of compounds **Th3(n)**, **Th-Oxd(n)** and **Th-Thd-Th(n)**.

Terthiophene derivatives are among the most extensively studied smectic LC organic semiconductors. Of particular interest, the 5,5”-di(*n*-alkyl)terthiophenes, **Th_3_(*n*)** (*n* = 7–10) (Figure 1), exhibit both crystalline lamellar phases at room temperature and smectic F (SmF) and C (SmC) phases at elevated temperatures [50,51,52,53,54,55]. Whereas charge carrier mobility increases in highly ordered lamellar crystalline phases [46,56,57] overlying smectic LC phases enable uniformly aligned, defect-free thin-films to be formed during processing [58]. For example, Hanna demonstrated that films of **Th_3_(8)** obtained from liquid crystalline phases at elevated temperatures exhibit improved OFET mobilities in the room temperature crystalline state [59,60]. In addition, samples cooled through multiple similar LCs, such as SmC and SmF phases, tend to possess improved morphologies [37,61].

The majority of studies on terthiophene-based mesogens have focused on modification of the terminal groups [37,50,51,52,62,63,64,65,66], whereas changes to the central terthiophene core have largely been neglected [57]. In the present work, we replaced the central thiophene of **Th_3_(*n*)** with either oxadiazole or thiadiazole rings to afford **Th-Oxd-Th(*n*)** and **Th-Thd-Th(*n*)**, respectively (Figure 1), and examined the impact of these changes on both the phase behavior and photophysical properties. While these rings are more electron-accepting than thiophene, allowing for the tuning of opto-electronic properties, their geometric similarities to thiophene and the precedent of oxadiazole- and thiadiazole-based mesogens in the literature [67,68,69,70,71] suggested that these compounds would be compatible with the formation of smectic phases similar to those of **Th_3_(*n*)**.

Our initial efforts focused on a comparison of the opto-electronic properties and self-assembly of **Th-Oxd-Th(10)** and **Th-Thd-Th(10)** to those of **Th_3_(10)**. Based on the promising liquid crystalline properties of **Th-Thd-Th(10)**, we extended our study to **Th-Thd-Th(*n*)** for (*n* = 4, 6, 8, 12) to more fully examine this series. To our knowledge, this represents the first systematic examination of either of these systems [72]. 

## 2. Results and Discussion

### 2.1. Synthesis

Our initial studies focused on the *n* = 10 derivatives because the parent terthiophene system **Th_3_(10)** was reported to form stable SmC and SmF LC phases [50]. **Th_3_(10)** was prepared in order to confirm the literature reports and to facilitate direct comparison to the newly synthesized thiadiazole and oxadiazole analogues. Terthiophene was obtained by Kumada coupling of 2,5-dibromothiophene and 2-bromothiophene [73]. Subsequent di-alkylation through treatment with n-butyl lithium, potassium t-butoxide and 1-bromodecane gave **Th_3_(10)** [48]. 

**Scheme 1:**
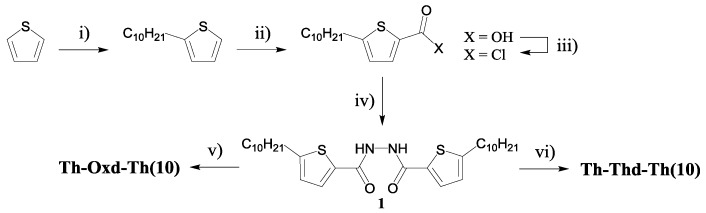
Synthetic route to **Th-Oxd-Th(10)** and **Th-Thd-Th(10)**. (**i**) (1) n-BuLi, THF, −78 °C; (2) C_10_H_21_Br, RT, 81%; (**ii**) (1) n-BuLi, THF, −78 °C; (2) CO_2_, RT, 69%; (**iii**) SOCl_2_, reflux; (**iv**) H_2_NNH_2_·H_2_O, Et_3_N, NMP, RT, 37%; (**v**) SOCl_2_, reflux, 64%; (**vi**) Lawesson’s reagent, toluene, reflux, 51%.

The terthiophene analogues incorporating oxadiazole and thiadiazole were synthesized according to Scheme 1. 2-Decylthiophene was prepared from thiophene according to literature methods [74]. A carboxylic acid group was installed in the 5-position by treatment with n-butyl lithium, followed by carbon dioxide [75]; this acid was then treated with thionyl chloride to produce the acid chloride in situ, two equivalents of which were condensed in the presence of hydrazine hydrate and triethylamine to give **1** [76]. The ring-closing of **1** in the presence thionyl chloride produced **Th-Oxd-Th(10)** [76]. Alternatively, treatment of **1** with Lawesson’s reagent produced **Th-Thd-Th(10)** [76].

**Scheme 2:**
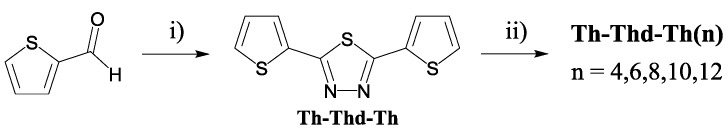
Synthetic route used to prepare the complete series of **Th-Thd-Th(*n*)** derivatives (*n* = 4, 6, 8, 12); (**i**) H_2_NNH_2_·H_2_O, sulfur, propanol, 150 °C, high pressure, 88%; (**ii**) (1) n-BuLi, t-BuOK, THF, −78 °C; (2) C_10_H_21_Br, RT, 29%–69%.

The alternative 2-step route shown in Scheme 2 was adopted for the synthesis of the remaining **Th-Thd-Th(*n*)** derivatives. The **Th-Thd-Th** parent compound was prepared in a one-pot reaction of 2-thiophenecarboxaldehyde, hydrazine hydrate, and sulfur in a high pressure vessel according to literature reports [77]. Di-alkylation of this intermediate by deprotonation with n-butyl lithium and potassium t-butoxide, followed by the appropriate 1-bromoalkane afforded the final target compounds.

### 2.2. Phase Behaviour

The liquid crystal and solid-state self-assembly of all compounds were investigated by differential scanning calorimetry (DSC), polarized optical microscopy (POM), variable temperature powder x-ray diffraction (VT-pXRD), and single crystal x-ray diffraction (SC-XRD), the results of which are summarized in Table 1. The complete data is available in Figures S1-25 and Tables S1-4 in the Supplementary Materials. In all cases, this phase behaviour was observed upon repeated heating/cooling cycles. TGA analysis (see Supplementary Materials) indicated that no decomposition occurs for any of compound studied below 250 °C. 

**Figure 2:**
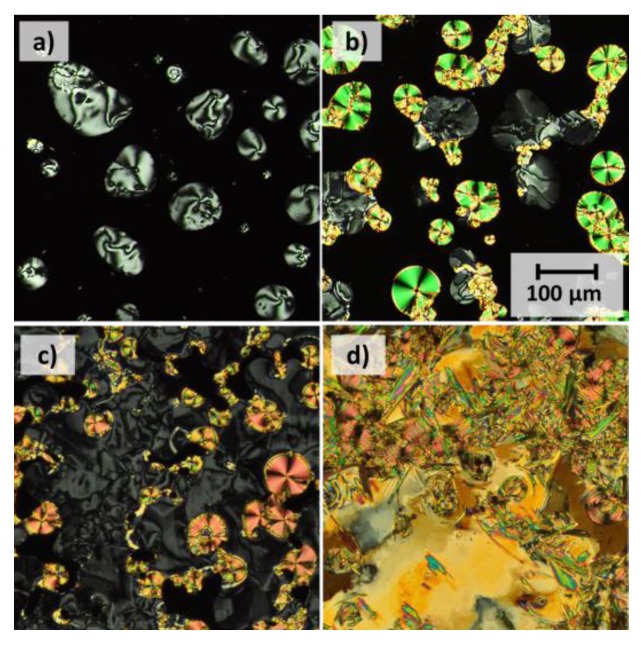
POM of **Th_3_(10)** showing schlieren (**a**) and focal-conic (**b**) textures of the SmC phase at 93.5 °C, modified textures of the SmF phase at 91.5 °C (**c**), and changes upon cooling to the crystalline phase at 65.0 °C (**d**).

**Th_3_(10)** was reported to form a narrow SmC phase from 92–95 °C, a SmF phase from 71–92 °C, and a lamellar crystalline solid at lower temperatures [50]. Our results are in agreement with these reports. POM images obtained around the clearing point show the emergence of schlieren and focal-conic fan textures (Figure 2a,b, respectively) from the isotropic liquid (dark regions), consistent with a SmC phase. We were unable to corroborate this assignment by XRD due to the narrow temperature range of this phase. 

Further cooling leads to a second fluid phase that exhibits POM textures (Figure 2c) resembling those of the overlying SmC phase, indicating that similar ordering is maintained. The XRD pattern (Figure S6) exhibits a sharp low-angle peak, assigned to d_001_ of a lamellar phase. The layer spacing (36.0 Å) is slightly smaller than the calculated molecular length (39.1 Å), which suggests a tilted phase, but could also result from interdigitation of the alkyl chains. The peak in the wide-angle region is sharper than that of typical fluid smectic phases such as SmA and SmC, consistent with an ordered lamellar phase such as SmF or SmI [78], both of which are common underlying phases for the SmC phase. Although we cannot rule out the formation of orthogonal phases based on the available data, our assignment of SmC and SmF phases is consistent with previous reports [50,51,52,53,54,55].

The transition into the next phase upon cooling is accompanied by dramatic changes in the POM, with the schlieren regions becoming bright and the fan textures distorted by striations (Figure 2d). The lack of fluidity under mechanical shearing confirms that this phase is a solid. No further changes are observed by POM upon cooling to room temperature. The room temperature XRD of this compound consists of a low-angle d_001_ peak along with higher order diffractions demonstrating that layered order is maintained; multiple sharp, intense peaks in the wide-angle region indicate increased intra-layer ordering (Figure S6).

In contrast, **Th-Oxd-Th(10)** was not liquid crystalline. The POM images of the phase immediately below the isotropic liquid display needle-like crystallites that do not shear (Figure S1). The XRD of this phase exhibits numerous sharp, low intensity peaks (Figure S7), consistent with a crystalline solid phase. 

**Figure 3:**
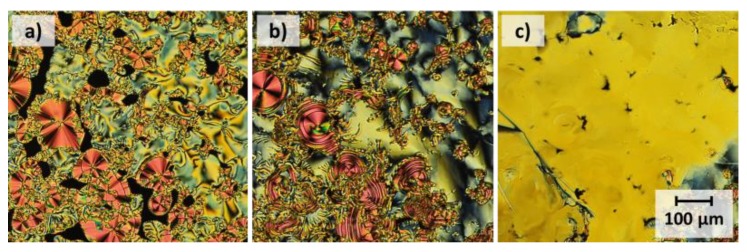
POM of **Th-Thd-Th(10)** showing schlieren and focal-conic textures of the SmC phase at 125.0 °C (**a**), modified textures of the CrJ phase at 91.0 °C (**b**), and changes upon cooling to the crystalline phase at 79.0 °C (**c**).

**Figure 4:**
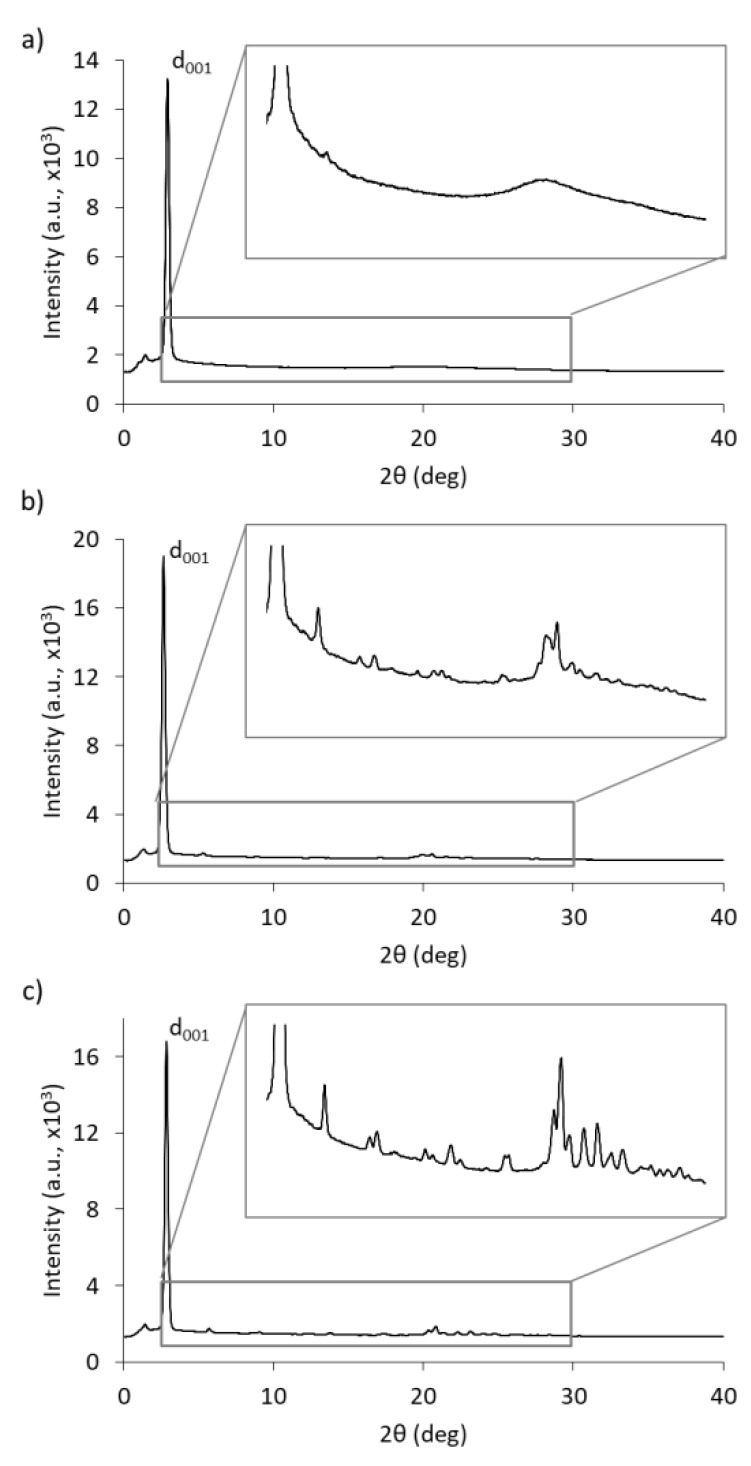
XRD of **Th-Thd-Th(10)** at 126 °C, in the SmC phase (**a**); at 88 °C, in the CrJ phase (**b**); and at 25 °C, in the crystalline phase (**c**).

The DSC of **Th-Thd-Th(10)** contains three peaks that are observed on repeated heating and cooling cycles (Figure S17). POM images of the high temperature phase obtained on cooling from the isotropic liquid are shown in Figure 3a. As observed for **Th_3_(10)**, the coexistence of these focal-conic fan and schlieren textures is characteristic of a SmC LC phase. This assignment was confirmed by XRD (Figure 4a). In the small angle region, a sharp d_001_ peak and a low intensity d_002_ diffraction can be identified, indicative of lamellar packing with a d-spacing of 30.1 Å. This layer spacing is considerably shorter than the calculated molecular length of 40.0 Å, suggesting that the molecules are tilted within the layers, although interdigitation may also account for a portion of this discrepancy. In the wide angle region, the broad, low intensity peak at a d-spacing of 4.37 Å is ascribed to the alkyl halo commonly observed for disordered smectic phases.

Upon further cooling below the transition at 91 °C, the POM textures develop striations across the fan-shaped regions, and the schlieren patterns blur into a mosaic texture (Figure 3b). These changes are commonly associated with the formation of more highly ordered smectic phases [47,48,79,80], which is further supported by the XRD data (Figure 4b). The lamellar d_001_, d_002_ and d_003_ peaks become more pronounced, signifying an increase in long-range layer ordering, and the layer spacing increases to 33.3 Å, from 30.1 Å in the SmC phase. In the wide angle region, several sharp peaks appear, associated with an increase in intra-layer ordering. This XRD pattern is more consistent with an ordered lamellar soft crystal G or J phase than SmF/I phases (see above). Single crystal studies discussed below indicate that the orientation of the molecules tilt towards the apex of the hexagonal intra-layer packing, and thus this phase was assigned as a CrJ phase.

Further cooling of this sample below 82 °C results in the loss of the preceding POM textures to give relatively featureless domains that are maintained down to room temperature (Figure 3c). The XRD of this phase is shown in Figure 4c. The sharp low-angle d_001_, d_002_, and d_003_ peaks demonstrate that the lamellar order is maintained. The pattern in the wide-angle region becomes even more complex with higher intensity peaks corresponding to increased intra-layer order, leading to the assignment of this room temperature phase as a highly ordered lamellar crystalline phase (Cr).

Prompted by the observation of LC properties of **Th-Thd-Th(10)**, we extended our study to derivatives with varying terminal chain lengths (*n*) for comparison to the literature **Th_3_(*n*)** series. The phase transitions of all compounds, as determined by DSC (Figures S14–18), are listed in Table 1. The POM and XRD results are shown in Figures S2–5 and Figures S8–11 respectively.

The shortest chain length derivative, terminated in butyl groups, is not liquid crystalline, but exhibits several polymorphic crystalline states. POM images of **Th-Thd-Th(4)** obtained on cooling from the isotropic liquid display a rigid, needle-like crystalline texture, and the XRD consists of numerous sharp, low intensity peaks.

At intermediate chain lengths, LC behaviour similar to that of **Th-Thd-Th(10)** is observed. The derivatives with *n* = 6 and 8 exhibit a SmC phase immediately below the isotropic liquid, as evidenced by characteristic schlieren and fan-shaped textures in the POM and XRD patterns consisting of a sharp low-angle d_001_ peak and a broad, low intensity alkyl halo in the wide-angle region. At the next phase transition, the POM images transform into mosaic and striated fan textures and the XRD shows higher order d_00*n*_ diffractions in the low-angle region and additional sharp peaks at wider angles. This phase was identified as a CrJ phase, due to the similarities to observations for **Th-Thd-Th(10)**. In the case of **Th-Thd-Th(6)**, this phase is maintained down to room temperature, whereas **Th-Thd-Th(8)** exhibits an additional room temperature lamellar crystalline phase similar to that of **Th-Thd-Th(10)**.

POM and XRD studies indicate that **Th-Thd-Th(12)** also forms SmC and CrJ phases. However, the DSC of this compound indicates the presence of an additional intermediate phase. The minimal changes in the POM textures and the low enthalpy change of the associated DSC peak suggest that this intermediate phase is similar to the preceding SmC phase. This is supported by the corresponding XRD pattern, which consists of a sharp d_001_ peak in the low-angle region and a peak of increased intensity and sharpness compared to the SmC phase at wider angles. These results, which are similar to those described for **Th_3_(10)** above, are consistent with SmF/I phases. This phase is tentatively assigned as a SmI LC, based on our more detailed analysis of **Th-Thd-Th(10)**. Like **Th-Thd-Th(8)** and **Th-Thd-Th(10)**, this dodecyl derivative exhibits a room temperature lamellar phase; the XRD patterns and POM textures of this phase remained unchanged for over a week, suggesting that it is thermodynamically stable. Similar results were also observed for the room temperature phases of the *n* = 6, 8, and 10 derivatives.

The persistence length of the layer ordering can be estimated based on the Debye–Scherrer equation applied to the d_001_ peak in the XRD. The resulting correlation lengths of **Th_3_(10)** in the SmF and Cr phases are 7 and 8 layers (26 and 27 nm), respectively. Those of **Th-Thd-Th(10)** in the SmC, CrJ, and Cr phases are estimated to be between 10–11 layers (31–34 nm), suggesting that the thiadiazole derivatives tend to exhibit more long range lamellar order than their terthiophene analogues.

**Figure 5:**
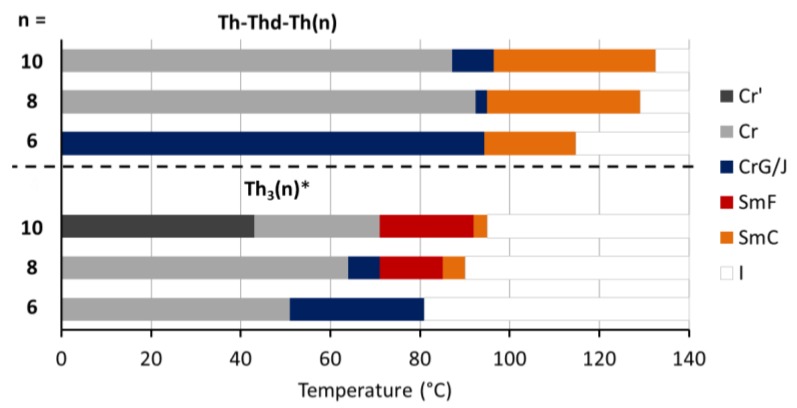
Phase behaviour of **Th-Thd-Th(*n*)** (this work) and **Th_3_(*n*)** (*literature) [74].

A comparison between the phase behavior of **Th-Thd-Th(*n*)** derivatives and their **Th_3_(*n*)** analogues [50] (*n* = 6, 8, 10) is shown in Figure 5. **Th_3_(4)** and **Th_3_(12)** were excluded from this analysis since reliable phase assignments are not available for either compound. Both series show a propensity to form layered structures, with SmC phases observed at higher temperatures in most cases, and lamellar crystalline phases formed at room temperature. With the exception of **Th_3_(10)**, compounds in both series exhibit soft crystalline G/J phases. The **Th_3_(10)** and **Th_3_(8)** derivatives exhibit an intermediate SmF phase; this phase is not observed for the **Th-Thd-Th(*n*)** derivatives shown, but a similarly ordered SmI phase emerges at longer chain length (**Th-Thd-Th(12)**, see above). Both series exhibit a tendency towards increased phase diversity at longer chain lengths. Liquid crystallinity is not observed for the shortest members of either series (**Th-Thd-Th(4)** and **Th_3_(*n*)** for *n* ≤ 6).

The **Th-Thd-Th(*n*)** compounds exhibit SmC phases at higher temperatures and over broader ranges than the corresponding **Th_3_(*n*)** derivatives. For example, **Th-Thd-Th(8)** forms a SmC phase over a 34 °C range (95–129 °C), whereas the SmC phase of **Th_3_(8)** is stable over only a 5 °C range (85–90 °C). As film processing from well-oriented but fluid states (e.g., SmA/C) is advantageous for obtaining uniformly aligned samples [37,58,59] and thermal annealing at elevated temperatures has been shown to improve film morphology [31,48,81], the increased SmC phase stabilities of the thiadiazole derivatives make them attractive candidates for device fabrication.

The highly ordered crystalline lamellar phases of **Th-Thd-Th(*n*)** are also stable to higher temperatures than those of **Th_3_(*n*)**. For example, the Cr phase of **Th-Thd-Th(8)** is maintained up to 92 °C, whereas **Th_3_(8)** melts into a liquid crystal at 71 °C. Charge carrier mobility tends to increase in more highly ordered phases [56,57,58], and phase transitions lead to discontinuity in performance [82,83]. Higher Cr-Sm transition temperatures such as those found for **Th-Thd-Th(*n*)** could therefore facilitate a wider range of operating temperatures and lower the chance of performance degradation due to thermally-induced transitions.

**Figure 6:**
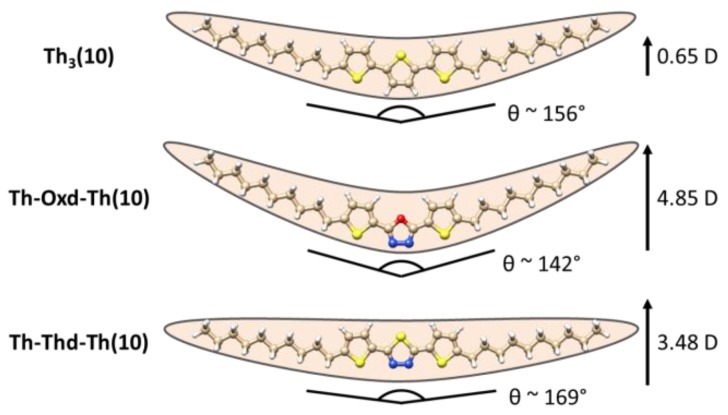
The optimized structures of the compounds studied, showing differences in bend angle, θ, and transverse dipole moment. See Supplementary Materials for calculation details.

The pronounced differences in phase behaviour between these series in general, and between **Th_3_(10)**, **Th-Thd-Th(10)**, and **Th-Oxd-Th(10)** in particular, can be rationalized based on changes in molecular shape and electronics. The density functional theory (DFT) optimized structures, determined using B3LYP/6-31G*, (Figure 6) indicate that the bend angle of the molecules, which is largely dictated by the bond angles at the 2- and 5-positions of the central heterocycle are 156°, 142°, and 169° for **Th_3_(10)**, **Th-Oxd-Th(10)**, and **Th-Thd-Th(10)**, respectively. Molecular shape is a key determinant for liquid crystallinity, with more linear structures promoting these phases [84,85,86,87]. Therefore, the larger angles in **Th_3_(10)** and **Th-Thd-Th(10)** favor LC phases, whereas the more bent structure of **Th-Oxd-Th(10)** would be detrimental for mesophase formation. This is consistent with previous literature suggesting that thiadiazole derivatives have a greater tendency to exhibit smectic mesophases than oxadiazole analogues [88,89,90,91]. 

The transverse dipole moments, i.e., those perpendicular to the long axis of the molecule, are 0.65 D, 4.85 D, and 3.48 D for **Th_3_(10)**, **Th-Oxd-Th(10)**, and **Th-Thd-Th(10)**, respectively. Because liquid crystalline self-assembly is strongly influenced by dipole–dipole interactions [85,92,93] the substantially larger dipole of **Th-Thd-Th(10)** may stabilize its LC phases relative to those of **Th_3_(10)**. More broadly, the elevated transition temperatures of **Th-Thd-Th(*n*)** compared to the corresponding terthiophenes **Th_3_(*n*)** can be attributed to a combination of the higher transverse dipole moments and more linear structures of the former series.

**Figure 7:**
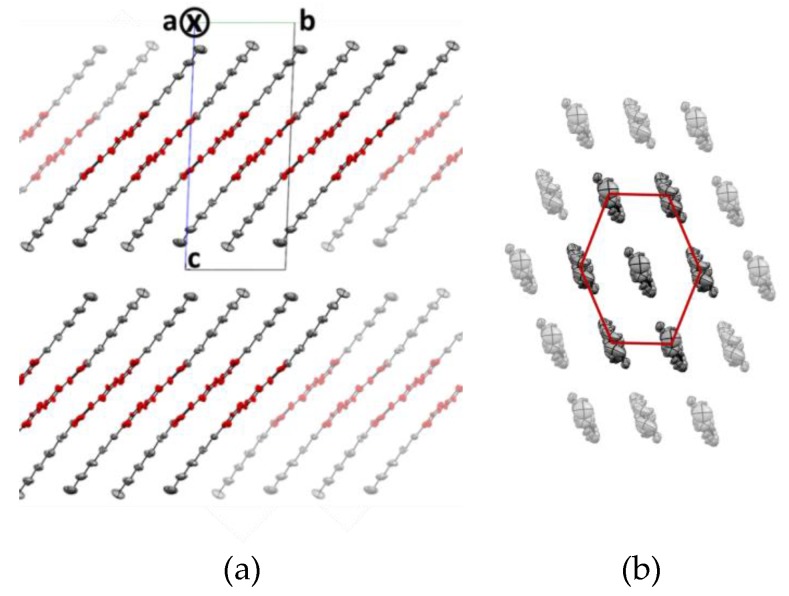
Crystal structure of **Th-Thd-Th(10)** showing lamellar order (**a**) and pseudo-hexagonal intra-layer packing (**b**). Legend: Alkyl chains in grey, aromatic core in red, hydrogens omitted for clarity.

In an effort to gain more insight into the solid-state structures of the phases, crystal growth via slow evaporation from a variety of solvents was explored. Suitable single crystals of **Th-Thd-Th**, **Th-Thd-Th(4)** and **Th-Thd-Th(10)** were obtained from acetonitrile or tetrahydrofuran and analyzed by XRD. The single crystal structure of **Th-Thd-Th(10)** demonstrates lamellar order with significant tilt angle (~32°, Figure 7a and Figure S19), as well as pseudo-hexagonal packing within each layer (Figure 7b), consistent with the phase assignments made by POM and XRD above. The single crystal structure of **Th-Thd-Th(4)** also shows lamellar order (Figure S21), with one butyl chain extending parallel to the core and the other approximately orthogonal. In contrast, both the decyl chains of **Th-Thd-Th(10)** extend along the direction of the core. This difference in the orientation of the alkyl chains provides a clue as to why the latter is liquid crystalline but the former is non-mesogenic.

The lamellar order in the crystal structure of **Th-Thd-Th(4)** is clearly evident in a low angle d_001_ peak in the corresponding simulated powder diffraction pattern (Figure S25). This peak is not observed in the experimental powder XRD (Figure S11), which is attributed to distinct polymorphs resulting from the different conditions under which the samples were prepared and measured. The simulated and experimental powder XRD patterns of **Th-Thd-Th(10)** are in good agreement (Figure S25) with some variation in the relative intensities and positions of the wide angle peaks, which likely can be ascribed to the temperature differences between the measurements. 

The crystal structures obtained for the thiadiazole derivatives can also be compared to literature reports of their terthiophene analogues. The crystal structure of **Th_3_(8)** reported by Lercher et al. [94] (Figure S23) also exhibits lamellar order with pseudo-hexagonal intra-layer packing, in line with our POM and XRD results. Consistent with the DFT models, the crystal structures show that **Th_3_(8)** is more bent than **Th-Thd-Th(10)**, with angles between the bonds at the 2 and 5 positions of the central heterocycle of 147° and 157°, respectively (Figure S20). The crystal structures differ with respect to the correlation of molecular orientation from one layer to the next. In **Th-Thd-Th(10)**, each successive layer has the same tilt direction (synclinic), whereas alternating layers exhibit opposing (anticlinic) tilt directions in the structure of **Th_3_(8)**.

**Figure 8:**
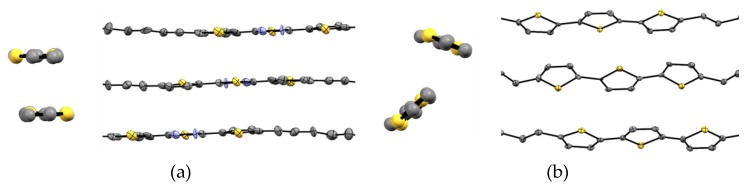
Packing of adjacent molecules in **Th-Thd-Th(10)** (**a**) and **Th_3_(8)** (**b**) as viewed along the long axis of the molecule (left) and the short axis of the molecule (right). Legend: sulphur in yellow, nitrogen in light blue, carbon in grey, hydrogens omitted for clarity.

Another distinction in the packing motif of the two compounds becomes evident upon closer inspection of the relative orientation of adjacent molecules within a layer. Figure 8a shows that for **Th-Thd-Th(10)** two molecules pack with their aromatic cores in a face-to-face fashion, with an anti-parallel arrangement. In contrast, molecules of **Th_3_(8)** orient in a herringbone configuration, with an approximate 55° angle between the planes of the molecules (Figure 8b). An increased tendency for face-to-face packing is also found in the crystal structures of the unsubstituted **Th-Thd-Th** core (Figure S22) compared to the analogous terthiophene **Th_3_** (Figure S24) [95]. The latter assumes a herringbone structure with molecules close to 90° to one another, while the former exhibits a modified herringbone-type configuration composed of co-facially packed pairs of molecules, with adjacent pairs arranged in a herringbone structure. **Th-Thd-Th(4)** shows a similar co-facial arrangement of adjacent molecules.

The stronger tendency of the thiadiazole derivatives to adopt face-to-face packing can be rationalized based on dipolar interactions. As already noted, DFT calculations indicate that **Th-Thd-Th(10)** has a much larger transverse dipole moment than **Th_3_(10)**, which favours the antiparallel stacked structures observed for the former. Similar effects have been noted in the self-assembly of discotic mesogens [92,93]. As a result, **Th-Thd-Th(10)** displays close intermolecular contacts, with a distance of 3.5 Å between the aromatic cores. These differences in the packing present a potential advantage for organic semiconductor applications since charge carrier transport is strongly dependent on close contacts and π–π overlap between adjacent molecules [18,96,97,98,99,100]. The stronger interactions within the layers of the thiadiazole analogues may also cause the increased transition temperatures of their LC and solid-state phases, as discussed above.

### 2.3. Molecular Opto-Electronic Properties

**Figure 9:**
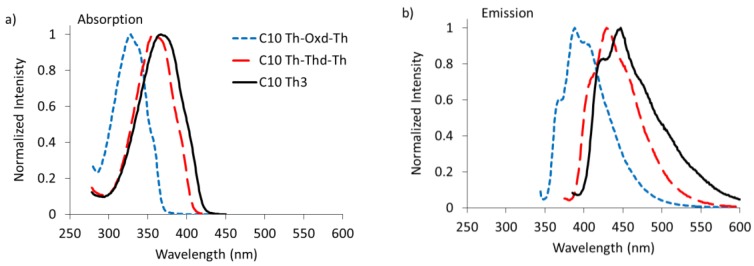
Absorption (**a**) and emission (**b**) spectra of **Th_3_(10)**, **Th-Oxd-Th(10)** and **Th-Thd-Th(10)** in CHCl_3_.

The UV-vis absorption spectra of **Th_3_(10)**, **Th-Thd-Th(10)**, and **Th-Oxd-Th(10)** in chloroform solutions (4 × 10^−5^ M) are shown in Figure 9a, with the data summarized in Table 2. The line shape and absorptivity values were independent of concentration (Figures S26-28). All three compounds feature a single strong absorption between 300 and 400 nm, with extinction coefficients greater than 25,000 M^−1^·cm^−1^. In all cases, these peaks are relatively broad and lack obvious fine vibrational structure, indicating the conformational flexibility of the ground state in solution, which has previously been demonstrated for oligothiophene systems [101,102]. The absorption band is blue-shifted on going from **Th_3_(10)** (λ_max_ = 367 nm) to **Th-Thd-Th(10)** (λ_max_ = 359 nm) to **Th-Oxd-Th(10)** (λ_max_ = 328 nm). Time-dependent density functional theory (TD-DFT) calculations reproduce the trend and shape of the experimental spectra, with absorption maxima of 383 nm (*f* = 1.17), 354 nm (*f* = 1.11), and 323 nm (*f* = 1.07) for **Th_3_(10)**, **Th-Thd-Th(10)**, and **Th-Oxd-Th(10)**, respectively. The TD-DFT results indicate that the observed UV-vis absorptions correspond to S_0_ to S_1_/HOMO-LUMO transitions from the π to π* orbitals. The optical band gaps calculated from the absorption onset were estimated to be 2.93, 3.04, and 3.35 eV for **Th_3_(10)**, **Th-Thd-Th(10)**, and **Th-Oxd-Th(10)**, respectively.

The emission spectra of **Th_3_(10)**, **Th-Thd-Th(10)** and **Th-Oxd-Th(10)** measured in chloroform (10^−6^–10^−5^ M) are shown in Figure 9b, with the data summarized in Table 2. The excitation spectra recorded match the UV-vis absorption spectra of the respective compounds (Figure S29). The emission spectra were recorded at the wavelength of maximum excitation intensity ($\lambda_{ex}^{max}$ = 340, 369, and 380 nm for **Th-Oxd-Th(10)**, **Th-Thd-Th(10)**, and **Th_3_(10)** respectively). All three compounds exhibit strong emission between 350–550 nm, with moderate 45–65 nm Stokes shifts. In contrast to the absorption spectra, the emission spectra display defined vibronic structure that is ascribed to the quinoidal character of the excited state, as previously reported for thiophene oligomers [101,102]. The fluorescence quantum yield (Φ_f_) of **Th_3_(10)**, determined relative to a quinine sulfate standard, was 0.56. Although this is higher than previously reported values, which range from 0.02 to 0.24, significant disparities in Φ_f_ under different experimental conditions are common in the literature [101,102,103]. **Th-Oxd-Th(10)** and **Th-Thd-Th(10)** exhibit similar fluorescence quantum yields of 0.48 and 0.49, respectively. These spectroscopic studies indicate that both the thiadiazole and oxadiazole derivatives exhibit strong absorption and emission, comparable to the parent terthiophene compound.

**Figure 10:**
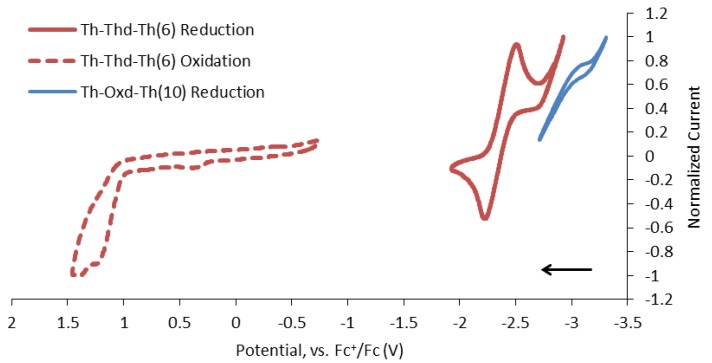
Cyclic voltammograms of 1.0 mM solutions showing reduction of Th-Thd-Th(6) (red) and Th-Oxd-Th(10) (blue) in THF with 0.1 M TBAP supporting electrolyte and oxidation of Th-Thd-Th(6) (red, dotted) in MeCN with 0.1 M TBAF supporting electrolyte.

The cyclic voltammograms of select compounds are shown in Figure 10, with the data summarized in Table 3. **Th-Thd-Th(6)** exhibits a quasi-reversible wave at −2.41 V, which we assign to a stable one-electron reduction process, as well as an irreversible oxidation at 1.18 V. Based on these reduction and oxidation waves measured relative to ferrocene, the HOMO and LUMO energy levels of **Th-Thd-Th(6)** were estimated as −5.98 and −2.39 eV, respectively, giving an electronic band gap of 3.59 eV. The corresponding theoretical values from DFT calculations (HOMO = −5.54, LUMO = −1.90 eV, band gap = 3.64 eV) are in reasonable agreement with the experimental numbers; the deviations are within the range reported for systems such as **Th_3_(6)** (see Table 3). The discrepancy between the electronic band gap (3.59 eV) and the optical gap (3.04 eV) yield an approximate exciton binding energy of 0.55 eV [104].

**Th-Oxd-Th(10)** displays a wave at −3.11 V which gives a corresponding LUMO energy of −1.69 eV, consistent with the DFT predicted value of −1.62 eV. The experimental reduction wave is irreversible, indicating that the reduced form of this compound is unstable. The oxidation wave of this compound could not be recorded due to a combination of low solubility and limitations imposed by the potential windows of the solvents.

The experimental and theoretical results show good agreement with the observed trend in the band gap, which was found to increase from **Th_3_(*n*)** to **Th-Thd-Th(*n*)** to **Th-Oxd-Th(*n*)**. In principle, we had anticipated the introduction of electron-poor thiadiazole and oxadiazole acceptors to decrease the band gap by lowering the LUMO level. However, experimentally, the reduction potential was found to increase successively from **Th_3_(*n*)** to **Th-Thd-Th(*n*)** to **Th-Oxd-Th(*n*)**. This is likely a consequence of decreased conjugation between the aromatic rings upon introduction of the thiadiazole or oxadiazole heterocycles, as has previously been demonstrated for mixed-ring oligomers [101,105,106]. We do not expect appreciable differences in these values for other homologs in this series since the length of terminal alkyl chains has a minimal impact on the opto-electronic properties [107,108], and DFT shows the electron distribution of the HOMO and LUMO molecular orbitals is concentrated on the aromatic rings (Figure S37).

## 3. Conclusions

In the present work, we examined the effects of replacing the central thiophene of liquid crystalline terthiophenes with either an oxadiazole or thiadiazole ring. This study revealed the pronounced impact of the central heterocycle on the LC properties of these systems. **Th_3_(10)** forms SmC, SmF, as well as room temperature lamellar crystalline phases. **Th-Oxd-Th(10)** does not display any LC phases but only polymorphic crystalline states. **Th-Thd-Th(10)** forms SmC, CrJ, and room temperature lamellar crystalline phases. All three classes of compound displayed strong absorption in the ultraviolet region and strong emission in the visible region, with band gaps between 3–3.5 eV.

We postulate that the pronounced differences in LC and solid-state self-assembly arise primarily due to the variation in bend angle and transverse dipole moment among the compounds, as corroborated by single crystal structures and DFT modelling studies. Compared to **Th_3_(*n*)**, the **Th-Thd-Th(*n*)** analogs exhibit SmC phases at higher temperatures and over broader ranges, maintain highly ordered lamellar phases to higher temperatures, possess longer correlation lengths of the lamellar order in the smectic phases, and display an increased tendency for molecules to assume face-to-face packing. In light of these differences, **Th-Thd-Th(*n*)** demonstrates potential as an organic semiconductor. Future studies will examine the performance of these materials in thin-film transistor (TFT) devices.

## 4. Experimental Part

### 4.1. Materials and Methods

All solvents used were reagent grade. 1-Bromooctane was purchased from TCI America. Triethylamine was purchased from Anachemia. All other reagents were purchased from Sigma-Aldrich. All reagents were used as received without further purification. Column chromatography was performed on silica gel 60 (230–400 mesh) purchased from Silicyle Inc. CDCl_3_ was obtained from Cambridge Isotope Laboratories Inc. 

400 MHz ^1^H NMR spectra were obtained on a Bruker AMX-400 400 MHz NMR spectrometer. 500 MHz ^1^H and 125 MHz ^13^C NMR spectra were obtained on a Varian AS500 Unity Inova 500 MHz NMR spectrometer. High resolution mass spectrometry was carried out on a Bruker micrOTOF II LC/MS (electrospray ionization, ESI^+^) by Nonka Sevova at Notre Dame Mass Spectrometry and Proteomics facility. Phase transition temperatures and enthalpies were determined using differential scanning calorimetry (DSC) on a TA Instruments DSC Q2000 equipped with a TA Instruments Refrigerated Cooling System 90, heating and cooling at a rate of 10 °C min^−1^. Thermogravimetric analysis was carried out on a Shimadzu TGA-50 with a heating rate of 2 °C per minute. Polarised optical microscopy was carried out using an Olympus BX50 microscope equipped with a Linkam LTS350 heating stage. X-ray scattering experiments were conducted using a Rigaku R-Axis Rapid diffractometer equipped with an in-house built temperature controller [109]. Single crystal x-ray diffraction data was collected on a Bruker Smart instrument equipped with an APEX II CCD area detector fixed at a distance from the crystal and a Cu Kα fine focus sealed tube (λ = 1.54178 Å) operated at 1.5 kW (45 kV, 0.65 mA), filtered with a graphite monochromator. The temperature was regulated using an Oxford Cryosystems Cryostream. UV-vis absorption spectroscopy was performed using a Varian Cary 300 Bio Spectrometer. Fluorescence measurements were performed using a Photon Technologies International (PTI) Quantamaster spectrofluorometer. Cyclic voltammetry scans were performed on a PAR-263A potentiometer with a glassy carbon working electrode, platinum counter electrode and silver wire reference electrode using 1.0 mM analyte solutions, 0.1 M supporting electrolyte, and Fc/Fc^+^ internal reference. Unconstrained DFT geometry optimization of all molecules was carried out at the B3LYP/6-31G* [110,111,112,113,114,115] level in Gaussian 1 [116], and time-dependent (TD-DFT) [117,118] calculations were performed using CAM-B3LYP/TZVP [119,120,121,122,123] in Gaussian 16 [116]. Further method details are given in the Supplementary Materials.

**Table 3:** Summary of the electrochemical properties of **Th-Oxd-Th(10)** and **Th-Thd-Th(6)** along with literature data for **Th_3_(6)**.

Compound	Oxidation (V)	Reduction (V)	Energy Levels (eV) [calc.]		Band Gap (eV)	
	*E*_1_^1/2^	*E*_1_^1/2^	HOMO ^a^	LUMO ^b^	*E*_g_^CV c^	*E*_g_^calc. d^
**Th-Oxd-Th(10)**	–	−3.11	[−5.59]	−1.69 [−1.62]	–	3.97
**Th-Thd-Th(6)**	1.18	−2.41	−5.98 [−5.54]	−2.39 [−1.90]	3.59	3.64
Th_3_(6)*	1.51	−2.14	−5.92 [−4.87]	−2.70 [−1.52]	3.22	3.35

*Literature reported properties [111]; ^a^ Calculated based on Fc HOMO = 4.8 eV, HOMO = −(*E*_1_^1/2^(ox) + 4.8); ^b^ Calculated based on Fc HOMO = 4.8 eV, LUMO = −(*E*_1_^1/2^(red) + 4.8); ^c^ Electrochemical band gap, *E*_g_^CV^ = *E_1_^1/2^(ox) – E_1_^1/2^(red);* ^d^ Theoretical band gap based on DFT (B3LYP/6-31G*) computations, *E*_g_^calc^ = *E_LUMO_ – E_HOMO_.*

### 4.2. Synthesis

#### 4.2.1. Precursors and Literature Compounds

The hydrazide derivative 1 was prepared by adapted literature procedures according to Scheme 1 above [76]. **Th_3_(10)** was prepared via modified literature reactions [73]. The **Th-Thd-Th** core was prepared under literature conditions as shown in Scheme 2 above [77]. Synthetic details for each of these compounds are given in the Supplementary Materials.

#### 4.2.2. **Th-Oxd-Th(10)**

In a flame-dried 10 mL three-neck round bottom flask, hydrazide derivative 1 (0.12 g, 0.23 mmol) was dissolved in thionyl chloride (5.0 mL, excess). After two hours of reflux under N_2_, the solution was cooled to room temperature. Remaining thionyl chloride was removed by rotary evaporation followed by high vacuum overnight. The crude was dissolved in chloroform (30 mL) and washed with water (2 × 30 mL), saturated sodium bicarbonate (30 mL), saturated sodium thiosulfate (30 mL), brine (30 mL) and then dried over magnesium sulfate. Further purification was done by column chromatography on silica treated with 2% triethylamine using 10% ethyl acetate in hexanes as the eluent. Recrystallization from ethanol afforded the product as a yellow solid (64% yield). Analytical data can be found in the Supplementary Materials. 

#### 4.2.3. **Th-Thd-Th(10)**

In a flame-dried 25 mL three-neck round bottom flask, hydrazide derivative 1 (0.40 g, 0.75 mmol) and 2,4-Bis(4-methoxyphenyl)-2,4-dithioxo-1,3,2,4-dithiadiphosphetane (Lawesson reagent, 0.38 g, 1.25 equivalents) were dissolved in toluene (15 mL). After 24 h of reflux under N_2_, the solution was cooled to room temperature. Toluene was removed by rotary evaporation followed by high vacuum overnight. The crude was dissolved in chloroform (30 mL) and washed with water (2 × 30 mL), saturated sodium bicarbonate (30 mL), saturated sodium thiosulfate (30 mL), brine (30 mL) and then dried over magnesium sulfate. Further purification was done by column chromatography on silica treated with 2% triethylamine using 10% ethyl acetate in hexanes as the eluent. Recrystallization from ethanol afforded the product as an orange solid (51% yield). Analytical data can be found in the Supplementary Materials. 

#### 4.2.4. General procedure for **Th-Thd-Th(*n*)** Derivatives

A flame-dried 50 mL three-neck round bottom flask was charged with **Th-Thd-Th** core (0.25 g, 1.0 mmol) and cycled between high vacuum and N_2_ atmosphere three times. Dry THF (25 mL) was added by cannula and the solution was cooled to around −78 °C prior to the drop-wise addition of n-butyllithium solution (2.5 M, 2.5 equivalents). After stirring for 30 min, the appropriate alkyl bromide, C*_n_*H_2*n*+1_Br, (4.0 equivalents) was added in one portion. This mixture was warmed to room temperature and stirred overnight. The reaction was quenched with 10% HCl (25 mL) and extracted with diethyl ether (3 × 30 mL). The combined organic fractions were washed with saturated sodium thiosulfate (2 × 80 mL), then water (100 mL) and dried over magnesium sulfate. Further purification was done by column chromatography on silica using a gradient from 2% to 10% of ethyl acetate in hexanes as the eluent. Recrystallization from hexanes afforded the product as a light orange solid (29%–69% yield). Analytical data can be found in the Supplementary Materials. 

## Figures and Tables

**Table 1:** Phase behaviour of the compounds studied.

Compound	Phase^a^	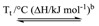	Phase^a^	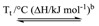	Phase^a^	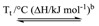	Phase^a^	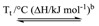	Phase^a^
**Th_3_(10)**	Cr’	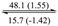	Cr	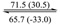	SmF	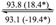	SmC	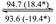	I
**Th-Oxd-Th(10)**			Cr”	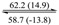	Cr’	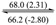	Cr	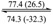	I
**Th-Thd-Th(4)**			Cr”	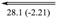	Cr’	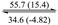	Cr	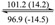	I
**Th-Thd-Th(6)**					CrJ	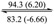	SmC	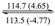	I
**Th-Thd-Th(8)**			Cr	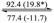	CrJ	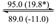	SmC	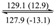	I
**Th-Thd-Th(10)**			Cr	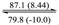	CrJ	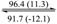	SmC	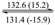	I
**Th-Thd-Th(12)**	Cr	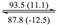	CrJ	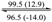	SmI	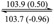	SmC	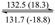	I

^a^ as identified by POM and XRD, Cr = crystalline, Sm = smectic, I = isotropic, see discussion for details; ^b^ transition temperatures and enthalpies determined by DSC at a scan rate = 10 °C/min, upon heating/cooling on top/bottom respectively; * combined enthalpy from overlapping peaks.

**Table 2:** Summary of the optical properties of the compounds studied.

Compound	$\lambda_{abs}^{max}$ (nm)	ε (M^−1^·cm^−1^)	*E*_g_^opt a^ (eV) [calc.]	$\lambda_{ex}^{max}$ (nm)	$\lambda_{em}^{max}$ (nm)	Φ_f_^b^
**Th-Oxd-Th(10)**	328	28200	3.35 [3.26]	340	387	0.48
**Th-Thd-Th(10)**	359	26800	3.04 [2.94]	369	430	0.49
**Th_3_(10)**	367	25900	2.93 [2.67]	380	443	0.56

^a^Optical bandgap, estimated by *E*_g_ = *h*c/$\lambda_{abs}^{onset}$, calc. value obtained from TD-DFT (CAM-B3LYP/TZVP/pcm); ^b^ Relative to quinine sulfate standard (Φ_f_ = 0.54) in 0.1 M H_2_SO_4._

## Supplementary Materials

Additional Figures S1-38 and Tables S1-10 are available online at https://www.mdpi.com/1996-1944/12/14/2314/s1.
