# Supplementary material for: Heterocycle Effects on the Liquid Crystallinity of Terthiophene Analogues
Source: Materials (Basel). 2019 Jul 19;12(14):2314. doi: 10.3390/ma12142314 (PMC6678929; doi:10.3390/ma12142314)
Supplement: Supplementary file 1 [file materials-12-02314-s001.pdf]

materials

Supporting information

Heterocycle Effects on the Liquid Crystallinity of Terthiophene Analogues

David F. Ester [1], Declan McKearney [1], Khrystyna Herasymchuk [1] and Vance E. Williams [1,*]

[1] Department of Chemistry, Simon Fraser University, 8888 University Drive, Burnaby, BC V5A 1S6 Canada; vancew@sfu.ca

1. Synthesis and Identification

All solvents used were reagent grade. 1-Bromooctane was purchased from TCI America. Triethylamine was purchased from Anachemia. All other reagents were purchased from Sigma-Aldrich. All reagents were used as received without further purification. Column chromatography was performed on silica gel 60 (230-400 mesh) purchased from Silicyle Inc. $CDCl_3$ was obtained from Cambridge Isotope Laboratories Inc.

400 MHz ^{1}H NMR spectra were run on a Bruker AMX-400 400 MHz NMR spectrometer. 500 MHz ^{1}H NMR spectra were run on a Varian AS500 Unity Inova 500 MHz spectrometer. High resolution mass spectrometry was carried out on a Bruker micrOTOF II LC/MS (ESI$^+$) by Nonka Sevova at Notre Dame Mass Spectrometry and Proteomics facility.

The final target compounds were prepared according to the procedures described in the experimental section of the paper. The synthetic procedures for any precursors and literature compounds are outlined below. The percent yield and detailed ^{1}H and ^{13}C NMR and mass spec data for all compounds are given below.

1.1. Precursors and literature compounds

2-Decylthiophene: In an oven-dried 100 mL 3-neck round bottom flask, thiophene (1.0 g, 11.9 mmol) in dry THF was cooled to -78 °C under a nitrogen atmosphere. n-Butyllithium (0.95 eq., 2.5 M solution in hexanes) was added drop-wise. The mixture was stirred at 0 °C for 60 minutes. Once cooled back to -78 °C, 1-bromodecane (0.90 eq.) was added slowly. The reaction was allowed to warm to room temperature and stirred overnight. It was then quenched with water, extracted with diethyl ether (3x50 mL), and the combined organic fractions were dried over magnesium sulfate. The crude was purified by vacuum distillation to give the product as a yellow oil (81 % yield). ^{1}H NMR (500 MHz, Chloroform-*d*) δ 7.18 (dd, *J* = 5.1, 1.1 Hz, 1H), 7.00 (dd, *J* = 5.1, 3.4 Hz, 1H), 6.87 (d, *J* = 3.2 Hz, 1H), 2.93 (t, *J* = 7.7 Hz, 2H), 1.79 (p, *J* = 7.4 Hz, 2H), 1.53 – 1.33 (m, 16H), 1.02 (t, *J* = 6.8 Hz, 3H) ppm.

5-Decyl-2-thiophenecarboxylic acid: In an oven-dried 100 mL 3-neck round bottom flask, 2-decylthiophene (1.0 g, 4.46 mmol) in dry diethyl ether was cooled to -78 °C under a nitrogen atmosphere. n-Butyllithium (1.2 eq., 2.5 M solution in hexanes) was added drop-wise. The mixture was stirred at 0 °C for 60 minutes. Once cooled back to -78 °C, carbon dioxide (excess) in the form of dry ice chunks was added. The reaction was allowed to warm to room temperature and stirred overnight. The precipitate was filtered and washed with diethyl ether then 10% hydrochloric acid and dried under high vacuum overnight to give the product as a white solid (69 % yield). ^{1}H NMR (500 MHz, Chloroform-*d*) δ 7.70 (d, *J* = 3.6 Hz, 1H), 6.80 (d, *J* = 3.5 Hz, 1H), 4.65 (s, 1H), 2.83 (t, *J* = 7.6 Hz, 2H), 1.69 (p, *J* = 7.5 Hz, 2H), 1.43 – 1.18 (m, 15H), 0.88 (t, *J* = 6.8 Hz, 3H) ppm.

Hydrazide derivative **1**: In an oven-dried 25 mL 3-neck round bottom flask, 5-decyl-2-thiophenecarboxylic acid (1.0 g, 3.52 mmol) was refluxed in thionyl chloride (5 mL, excess) under a nitrogen atmosphere for 2 hours. After cooling to room temperature, remaining thionyl chloride was removed via high vacuum overnight to generate 5-decyl-2-thiophenecarbonyl chloride in situ. This –oyl chloride was added to an oven-dried 50 mL 3-neck round bottom flask containing dry NMP. Triethylamine (3 mL, excess) was added followed by the drop-wise addition of hydrazine hydrate (0.7 eq.) at 0 °C. The mixture was warmed to room temperature and stirred overnight. The resulting

precipitate was collected by filtration. This crude product was purified by column chromatography on silica treated with 2% trimethylamine using a gradient from 0 to 50 % of ethyl acetate in hexanes as the eluent. The pure product was isolated as a white powder (37 % yield). ^{1}H NMR (500 MHz, Chloroform-*d*) δ 9.04 (s, 2H), 7.52 (d, *J* = 3.7 Hz, 2H), 6.76 (d, *J* = 3.7 Hz, 2H), 2.81 (t, *J* = 7.6 Hz, 4H), 1.67 (p, *J* = 7.5 Hz, 4H), 1.26 (s, 28H), 0.88 (t, *J* = 6.9 Hz, 6H) ppm.

α-Terthienyl, **Th**$_3$: Magnesium turnings (0.90 g, 37.0 mmol) were added to a 100 mL 3-neck round bottom flask, which was then sealed, purged with N_2, and flame dried. Added dry diethyl ether (50 mL) and cooled to 0 °C, followed by slow addition of 2-bromothiophene (3.26 mL, 33.7 mmol). Solution was warmed to room temperature and then refluxed for 2 hours to generate the Grignard solution. An oven-dried 250 mL 3-neck round bottom flask was charged with [1,3-bis(diphenylphosphino)propane] dichloronickel(II) (catalytic, ~2%) and cycled between N_2 and vacuum three times. Dry diethyl ether (150 mL) and then 2,5-dibromothiophene (1.71 mL, 15.3 mmol) were added and the solution was cooled to 0 °C. The Grignard solution prepared in situ was then added dropwise, the reaction was warmed to room temperature and then refluxed for 2 hours. After cooling to room temperature, the reaction was quenched by slow addition of 10% hydrochloric acid, the organic was separated out, and the aqueous was further extracted with diethyl ether (3x75 mL). The combined organic fractions were dried over magnesium sulfate. The crude was purified by column chromatography on silica using 5% ethyl acetate in hexanes as the eluent to give the final product (57 % yield). ^{1}H NMR (500 MHz, Chloroform-*d*) δ 7.23 (dd, *J* = 5.1, 1.2 Hz, 2H), 7.18 (dd, *J* = 3.6, 1.2 Hz, 2H), 7.09 (s, 2H), 7.03 (dd, *J* = 5.1, 3.6 Hz, 2H) ppm.

Th$_3$**(10)**: A flame-dried 50 mL three-neck round bottom flask was charged with **Th**$_3$ (0.15 g, 0.60 mmol) and cycled between high vacuum and N_2 atmosphere three times. Dry THF (25 mL) was added by cannula and the solution was cooled to around -78 °C prior to the drop-wise addition of n-butyllithium solution (2.5 M, 2.5 equivalents). After stirring for 30 minutes, potassium t-butoxide (1.0 M solution, 2.5 equivalents) was added and the solution was stirred for another 30 minutes. Then 1-decylbromide (2.2 equivalents) was added drop-wise. This mixture was warmed to room temperature and stirred overnight. The reaction was quenched with water (40 mL) and extracted with hexanes (3x30 mL). The combined organic fractions were dried over magnesium sulfate. Further purification was done by column chromatography on silica using hexanes as the eluent. Recrystallization from ethanol afforded the product as a light yellow solid (49 % yield). ^{1}H NMR (500 MHz, Chloroform-*d*) δ 6.96 (t, *J* = 3.3 Hz, 4H), 6.67 (d, *J* = 3.4 Hz, 2H), 2.78 (t, *J* = 7.6 Hz, 4H), 1.67 (p, *J* = 7.4 Hz, 4H), 1.42 – 1.21 (m, 28H), 0.92 – 0.84 (m, 6H) ppm. ^{13}C NMR (101 MHz, Chloroform-*d*) δ 145.56, 136.29, 134.79, 124.92, 123.60, 123.31, 32.06, 31.76, 30.35, 29.76, 29.71, 29.52, 29.48, 29.24, 22.84, 14.27 ppm.

Th-Thd-Th *core*: In a 150 mL high pressure vessel, 2-thiophenecarboxaldehyde (1.5 g, 13.4 mmol), sulfur (1.5 equivalents), and hydrazine hydrate (4.0 equivalents) were combined in propanol (10 mL). The mixture was allowed to react for 4 hours at 150 °C under high pressure. Once cooled to room temperature, the precipitate was collected by vacuum filtration. The crude was then dissolved in dichloromethane (150 mL), washed with saturated sodium sulfide (2x120 mL), water (1x150 mL), and then dried over magnesium sulfate. Recrystallization from ethanol afforded the final product as a light orange solid (88 % yield). ^{1}H NMR (500 MHz, Chloroform-*d*) δ 7.56 (dd, *J* = 3.7, 1.1 Hz, 2H), 7.50 (dd, *J* = 5.1, 1.1 Hz, 2H), 7.13 (dd, *J* = 5.0, 3.7 Hz, 2H) ppm. ^{13}C NMR (126 MHz, Chloroform-*d*) δ 161.18, 132.35, 129.65, 129.55, 128.14 ppm.

1.2. Oxadiazole derivative

Th-Oxd-Th(10): 64% yield. ^{1}H NMR (500 MHz, Chloroform-*d*) δ 7.60 (d, *J* = 3.7 Hz, 2H), 6.84 (dd, *J* = 3.7, 1.0 Hz, 2H), 2.87 (t, *J* = 7.6 Hz, 4H), 1.72 (p, *J* = 7.6 Hz, 4H), 1.42 – 1.23 (m, 30H), 0.88 (t, *J* = 6.9 Hz, 6H) ppm. ^{13}C NMR (126 MHz, Chloroform-*d*) δ 160.25, 151.93, 129.83, 125.48, 122.39, 32.04, 31.61, 30.40, 29.72, 29.67, 29.46, 29.17, 22.83, 14.27 ppm. HRMS (ESI$^+$ of m + H$^+$): m/z calcd for $C_{30}H_{47}N_2OS_2$: 515.3085, found: 515.3124.

1.3. Thiadiazole derivatives

Th-Thd-Th(4): 62% yield. ^{1}H NMR (500 MHz, Chloroform-*d*) δ 7.35 (d, *J* = 3.7 Hz, 2H), 6.79 (dd, *J* = 3.7, 1.0 Hz, 2H), 2.89 – 2.81 (m, 4H), 1.75 – 1.66 (m, 4H), 1.48 – 1.36 (m, 4H), 0.95 (t, *J* = 7.4 Hz, 6H) ppm. ^{13}C NMR (126 MHz, Chloroform-*d*) δ 160.96, 150.97, 129.74, 129.53, 125.30, 33.68, 30.15, 22.28,13.96 ppm. HRMS (ESI$^+$ of m + H$^+$): m/z calcd for $C_{18}H_{23}N_2S_3$: 363.0979, found: 363.1018.

Th-Thd-Th(6): 29% yield. ^{1}H NMR (500 MHz, Chloroform-*d*) δ 7.35 (d, *J* = 3.7 Hz, 2H), 6.79 (dd, *J* = 3.7, 1.0 Hz, 2H), 2.85 (t, *J* = 7.6 Hz, 4H), 1.71 (p, *J* = 7.6 Hz, 4H), 1.43 – 1.29 (m, 12H), 0.92 – 0.87 (m, 6H) ppm. ^{13}C NMR (126 MHz, Chloroform-*d*) δ 160.97, 151.04, 129.73, 129.54, 125.29, 31.57, 30.47, 28.86, 22.70, 14.24, 14.19 ppm. HRMS (ESI$^+$ of m + H$^+$): m/z calcd for $C_{22}H_{31}N_2S_3$: 419.1605, found: 419.1644.

Th-Thd-Th(8): 30% yield. ^{1}H NMR (500 MHz, Chloroform-*d*) δ 7.35 (d, *J* = 3.7 Hz, 2H), 6.79 (d, *J* = 3.7 Hz, 2H), 2.84 (t, *J* = 7.6 Hz, 4H), 1.71 (p, *J* = 7.6 Hz, 4H), 1.43 – 1.21 (m, 20H), 0.88 (t, *J* = 6.9 Hz, 6H) ppm. ^{13}C NMR (126 MHz, Chloroform-*d*) δ 160.97, 151.03, 129.73, 129.52, 125.28, 31.98, 31.61, 30.46, 29.37, 29.20, 22.80, 14.27, 14.23 ppm. HRMS (ESI$^+$ of m + H$^+$): m/z calcd for $C_{26}H_{39}N_2S_3$: 475.2231, found: 475.2270.

Th-Thd-Th(10): 51% yield. ^{1}H NMR (500 MHz, Chloroform-*d*) δ 7.35 (d, *J* = 3.7 Hz, 2H), 6.79 (d, *J* = 3.7 Hz, 2H), 2.84 (t, *J* = 7.6 Hz, 4H), 1.71 (p, *J* = 7.5 Hz, 4H), 1.43 – 1.22 (m, 28H), 0.88 (t, *J* = 6.9 Hz, 6H) ppm. ^{13}C NMR (126 MHz, Chloroform-*d*) δ 160.97, 151.04, 129.74, 129.52, 125.29, 32.05, 31.61, 30.48, 29.73, 29.68, 29.47, 29.20, 22.84, 14.27 ppm. HRMS (ESI$^+$ of m + H$^+$): m/z calcd for $C_{30}H_{47}N_2S_3$: 531.2857, found: 531.2896.

Th-Thd-Th(12): 69% yield. ^{1}H NMR (500 MHz, Chloroform-*d*) δ 7.35 (d, *J* = 3.6 Hz, 2H), 6.79 (d, *J* = 3.7 Hz, 2H), 2.84 (t, *J* = 7.6 Hz, 4H), 1.71 (p, *J* = 7.6 Hz, 4H), 1.41 – 1.22 (m, 36H), 0.88 (t, *J* = 6.9 Hz, 6H) ppm. ^{13}C NMR (126 MHz, Chloroform-*d*) δ 160.96, 151.03, 129.74, 129.51, 125.29, 32.07, 31.61, 30.47, 29.78, 29.68, 29.50, 29.46, 29.48, 29.19, 22.85, 14.27 ppm. HRMS (ESI$^+$ of m + H$^+$): m/z calcd for $C_{34}H_{55}N_2S_3$: 587.3483, found: 587.3522.

2. Polarized Optical Microscopy (POM)

Polarized optical microscopy was carried out using an Olympus BX50 microscope equipped with a Linkam LTS350 heating stage. All images shown are of a size of ca. 920 x 1400 μm.

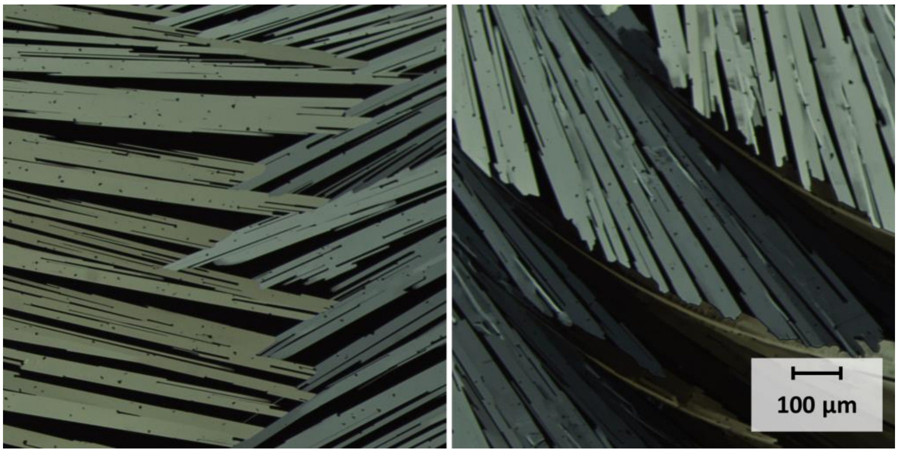

Figure S1. POM of **Th-Oxd-Th(10)** showing needle-like textures of the crystalline state at 72.0 °C.

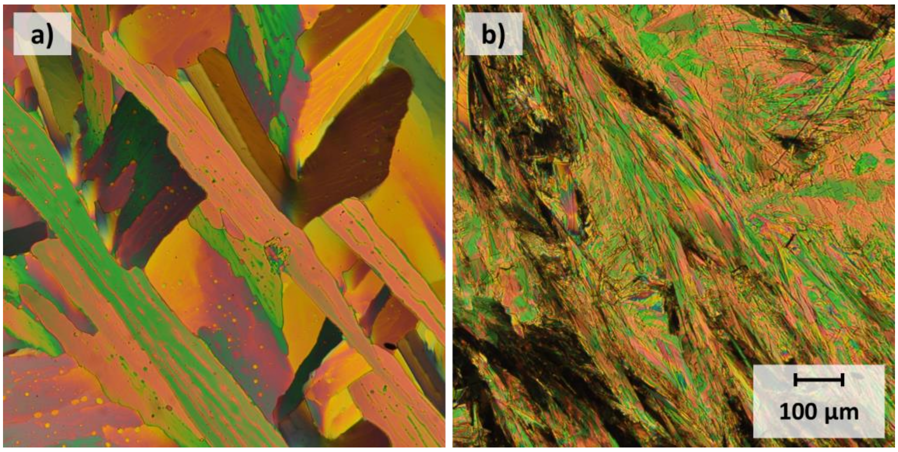

Figure S2. POM of **Th-Thd-Th(4)** showing needle-like textures of the crystalline state at 94.0 °C (a) and at 25.0 °C (b).

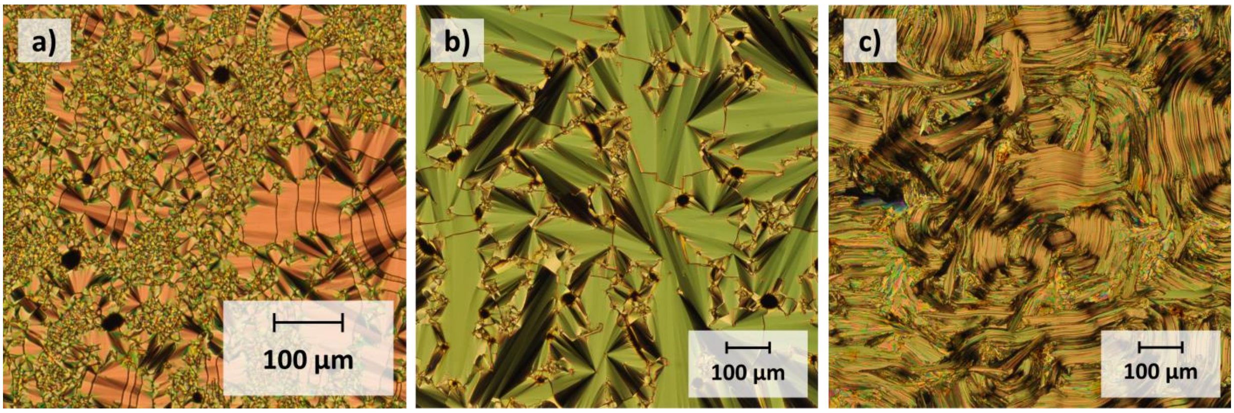

Figure S3. POM of **Th-Thd-Th(6)** showing schlieren and focal-conic textures of the SmC phase at 111.0 °C (a and b) and striations across focal-conic textures of the CrJ phase at 25.0 °C (c).

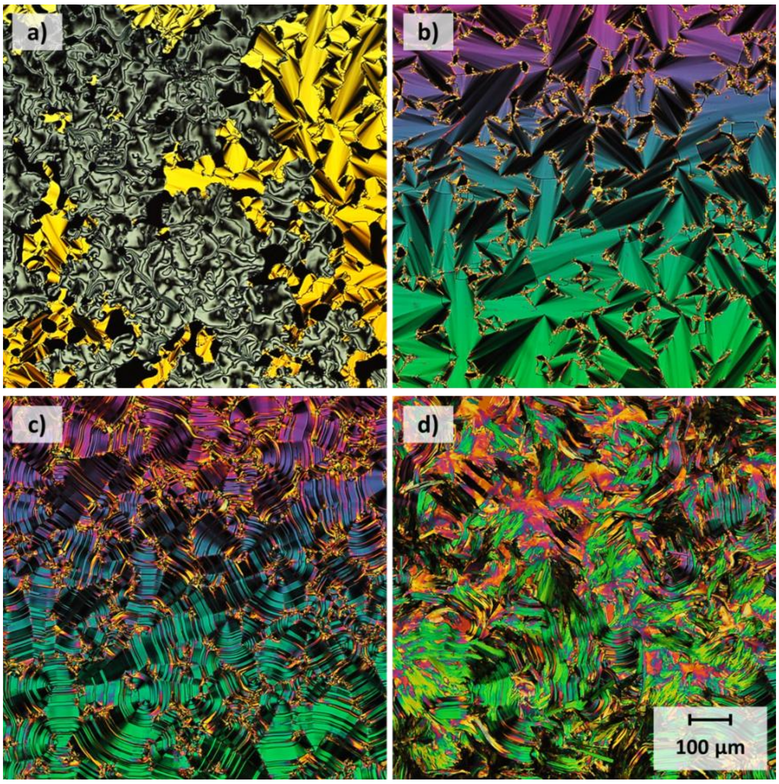

Figure S4. POM of **Th-Thd-Th(8)** showing schlieren and focal-conic textures of the SmC phase at 125.0 °C (a and b), striations across focal-conic textures of the CrJ phase at 88.0 °C (c), and changes upon cooling to the crystalline phase at 74.0 °C (d).

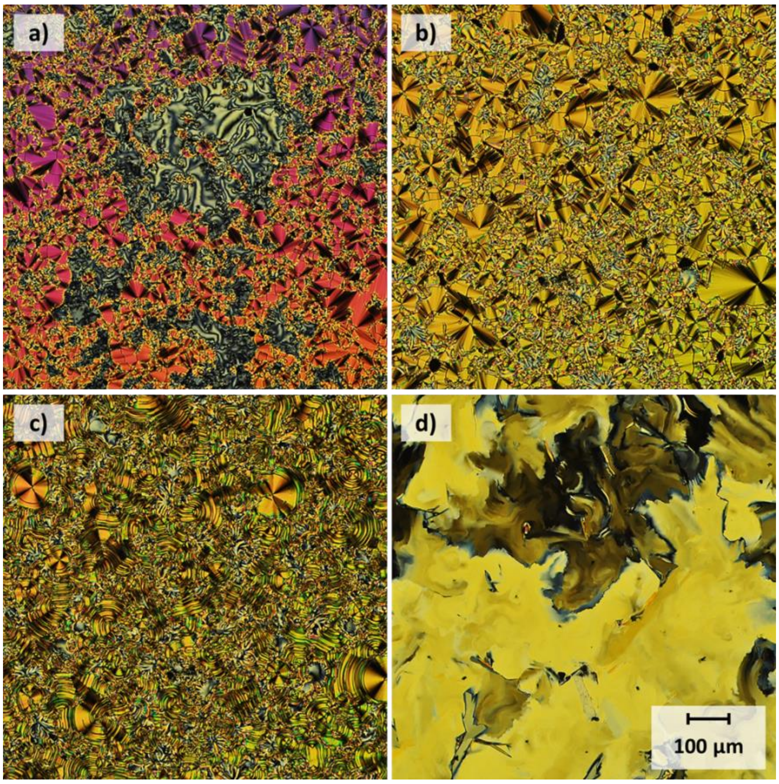

Figure S5. POM of **Th-Thd-Th(12)** showing schlieren and focal-conic textures of the SmC phase at 131.0 °C (a and b respectively), striations across focal-conic textures of the CrJ phase at 96.0 °C (c), and changes upon cooling to the crystalline phase at 87.0 °C (d).

3. Variable Temperature Powder X-ray Diffraction (VT-XRD)

X-ray scattering experiments were conducted using a Rigaku R-Axis Rapid diffractometer equipped with an in-house built temperature controller. [1]

Table 1. XRD parameters of the phases of **Th$_3$(10)** and **Th-Thd-Th(n)** (n = 6,8,10,12).

Compound (molecular length^a, nm)	Temperature (°C)	Phase	Layer spacing^b (nm)	Correlation length^c (nm)	# of layers	Tilt angle, θ (°)^d
**Th$_3$(10)** (3.91)	91	SmF	3.60	26	7	23
	25	Cr	3.85	27	8	10
**Th-Thd-Th(6)** (2.95)	110	SmC	2.26	31	13	40
	76	CrJ	2.28	36	16	39
	25	CrJ	2.17	38	17	43
**Th-Thd-Th(8)** (3.47)	124	SmC	2.65	25	9	40
	83	CrJ	2.93	31	11	32
	25	Cr	2.49	38	15	44
**Th-Thd-Th(10)** (4.00)	126	SmC	3.01	31	10	41
	88	CrJ	3.33	32	10	34
	25	Cr	3.10	34	11	39
**Th-Thd-Th(12)** (4.49)	127	SmC	3.38	29	9	41
	101	SmI	3.66	29	8	35
	95	CrJ	3.69	25	7	35
	25	Cr	3.38	29	9	41

^{a}calculated by DFT using B3LYP/6-31G*; ^{b}determined based on d_{001} peak; ^{c}calculated using Debye-Scherrer equation applied to d_{001} peak; ^{d}calculated from molecular length and layer spacing

Table S2. XRD data of **Th$_3$(10)** and **Th-Thd-Th(n)** (n=6,8,10,12).

Compound (molecular length^a, Å)	Temperature (°C)	Phase	d-spacings (Å)	Miller indices (hkl)
**Th$_3$(10)** (39.1)	91	SmF	36.0	001
			4.37	110
	25	Cr	38.5	001
			23.3	---
			19.6	002
			4.37	110
			3.78	---
			3.06	---
**Th-Thd-Th(6)** (29.5)	110	SmC	22.7	001
			4.37	alkyl halo
	76	CrJ	22.7	001
			11.4	002
			9.08	---

			7.22	003
			6.65	---
			6.24	---
			5.55	004
			4.82	---
			4.43	110
			3.62	---
			3.54	---
			3.27	---
			3.20	---
	25	CrJ′	21.7	001
			10.9	002
			7.12	003
			6.45	---
			5.78	---
			4.78	---
			4.34	110
			4.03	---
			3.55	---
			3.16	---
			3.02	---
**Th-Thd-Th(8)** (34.7)	124	SmC	26.5	001
			4.37	alkyl halo
	83	CrJ	29.3	001
			14.7	002
			9.83	003
			7.06	004
			6.73	---
			5.18	---
			4.87	---
			4.45	110
			4.29	---
			4.11	---
			3.81	---
			3.58	---
			3.30	---
	25	Cr	24.9	001
			12.5	002
			8.79	003
			5.78	---
			4.75	---
			4.32	110
			4.15	---

			3.54	---
			3.29	---
			3.14	---
**Th-Thd-Th(10)** (40.0)	126	SmC	30.1	001
			15.1	002
			4.37	alkyl halo
	88	CrJ	33.3	001
			16.7	002
			11.1	003
			9.94	---
			8.84	---
			7.59	---
			5.16	---
			4.29	110
			4.27	---
			4.07	---
			3.98	---
			3.80	---
	25	Cr	31.0	001
			15.4	002
			9.78	003
			7.25	---
			6.36	---
			5.04	---
			4.31	---
			4.22	110
			4.11	---
			3.94	---
			3.79	---
			3.65	---
			3.54	---
**Th-Thd-Th(12)** (44.9)	127	SmC	33.8	001
			17.1	002
			4.37	alkyl halo
	101	SmI	36.6	001
			18.5	002
			4.37	110
	95	CrJ	36.9	001
			18.8	002
			12.4	003
			9.98	---
			8.95	004

			5.13	---
			4.52	---
			4.33	110
			4.24	---
			4.02	---
			3.88	---
			3.74	---
			3.52	---
			33.8	001
			17.1	002
			11.8	003
			9.83	---
			8.61	004
			7.65	---
	25	Cr	5.07	---
			4.26	110
			4.17	---
			3.80	---
			3.67	---
			3.55	---
			3.44	---

[a]calculated by DFT using B3LYP/6-31G*

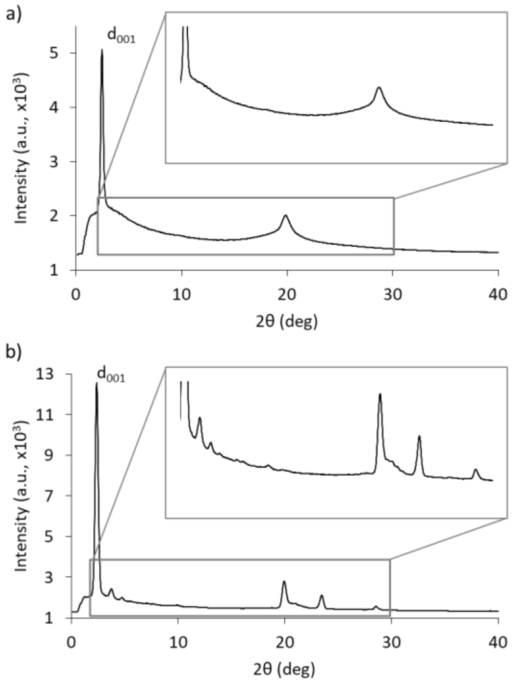

Figure S6. XRD of **Th**$_3$**(10)** at 91 °C, in the SmF phase (a) and at 25 °C, in the crystalline phase (b).

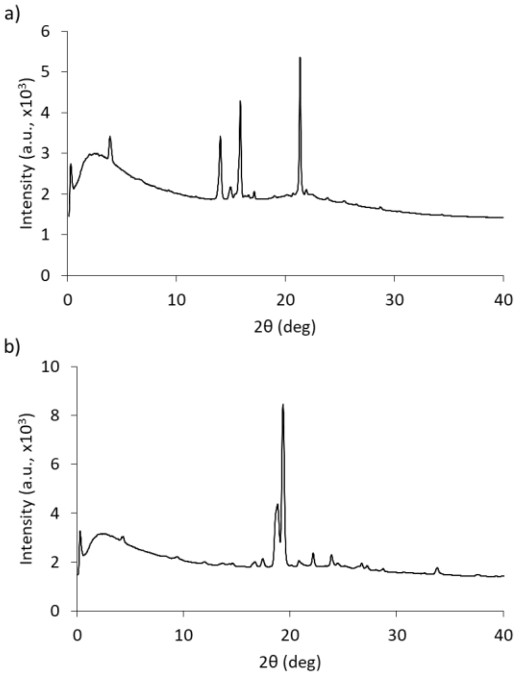

Figure S7. XRD of **Th-Oxd-Th(10)** at 70 °C, in the Cr phase (a) and at 25 °C, in the Cr″ phase (b).

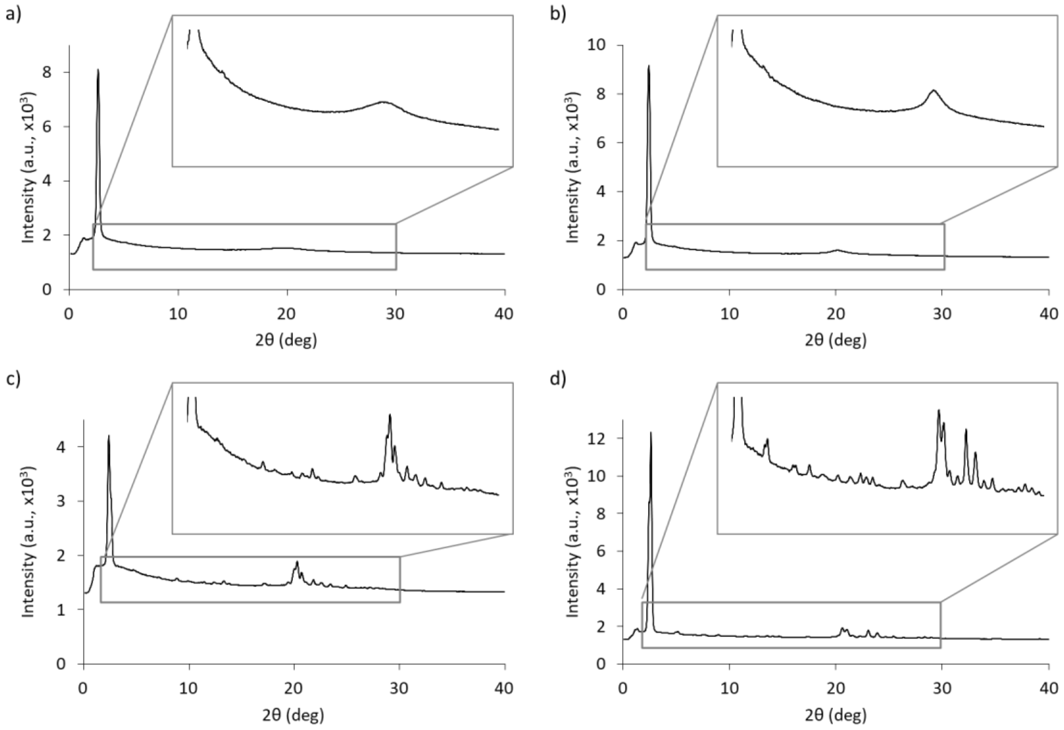

Figure S8. XRD of **Th-Thd-Th(12)** at 127 °C, in the SmC phase (a); at 101 °C, in the SmI phase (b); at 95 °C, in the CrJ phase (c); and at 25 °C, in the crystalline phase (d).

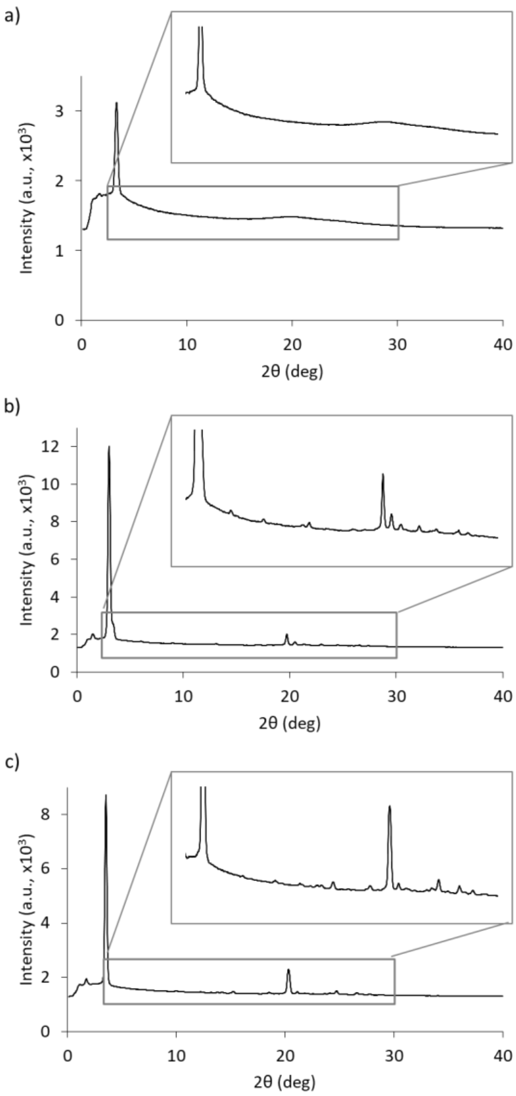

Figure S9. XRD of **Th-Thd-Th(8)** at 124 °C, in the SmC phase (a); at 83 °C, in the CrJ phase (b); and at 25 °C, in the crystalline phase (c).

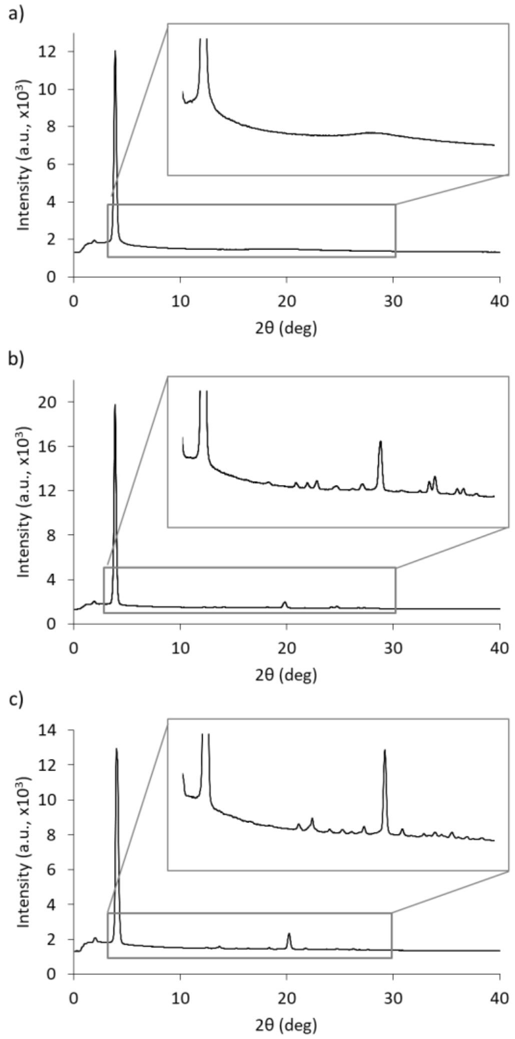

Figure S10. XRD of **Th-Thd-Th(6)** at 110 °C, in the SmC phase (a); at 76 °C, in the CrJ phase (b); and at 25 °C, in the crystalline phase (c).

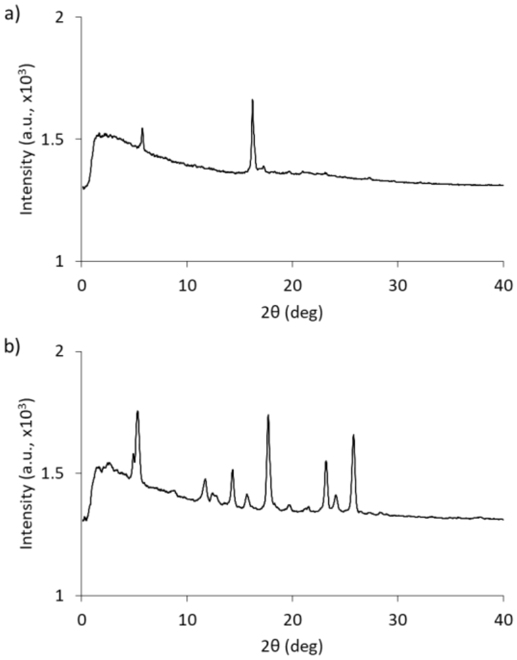

Figure S11. XRD of **Th-Thd-Th(4)** at 94 °C, in the Cr phase (a) and at 25 °C, in the Cr″ phase (b).

4. Differential Scanning Calorimetry (DSC)

Phase transition temperatures and enthalpies were determined using differential scanning calorimetry (DSC) on a TA Instruments DSC Q2000 equipped with a TA Instruments Refrigerated Cooling System 90, heating and cooling at a rate of 10˚C min^{-1}.

Table S3. Phase transition temperatures and enthalpies of **Th$_3$(n)** reported by Boucher et al. [2].

n	Transition Temperature/ °C [enthalpy/ kJ mol^{-1}]
**5**	**Cr** 46 [15.2] **CrG** 76 [13.6] **I**
**6**	**Cr** 51 [16.7] **CrG** 81 [16.4] **I**
**7**	**Cr** 52 [17.8] **CrG** 75 [3.8] **SmF** 82 [0.7] **SmC** 86 [7] **I**
**8**	**Cr** 64 [24.4] **CrG** 71 [3.3] **SmF** 85 [1.6] **SmC** 90 [7.3] **I**
**9**	**Cr** 66 [30.8] **SmF** 91 [1.8] **SmC** 94 [10.4] **I**
**10**	**Cr1** 43 [2.3] **Cr2** 71 [32.1] **SmF** 92 [2.7] **SmC** 95 [10.5] **I**

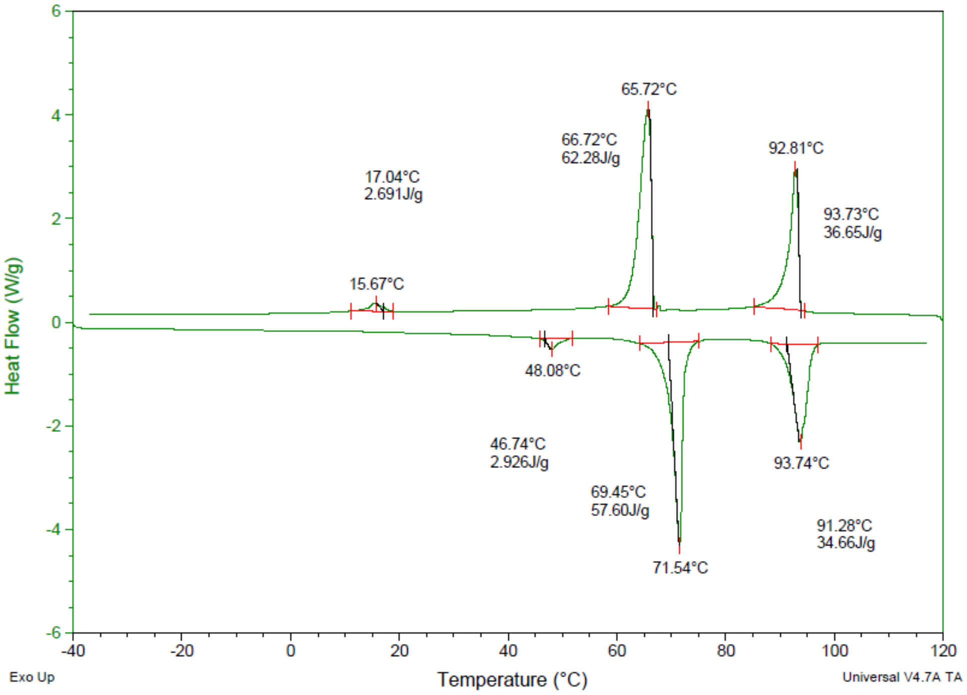

(a)

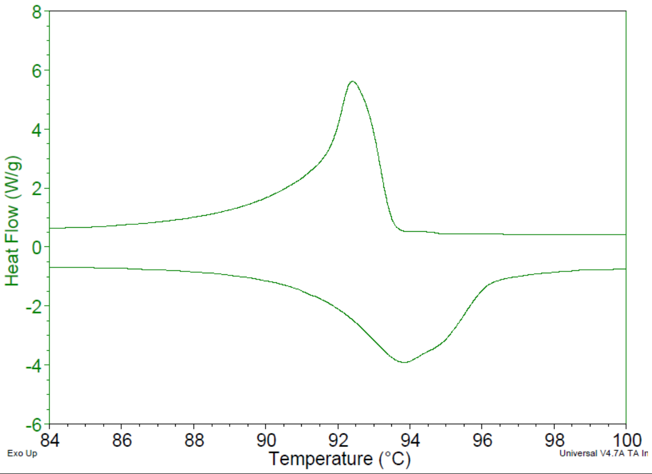

(b)

Figure S12. DSC thermogram of **Th$_3$(10)** (a), including zoomed in high temperature peak (b).

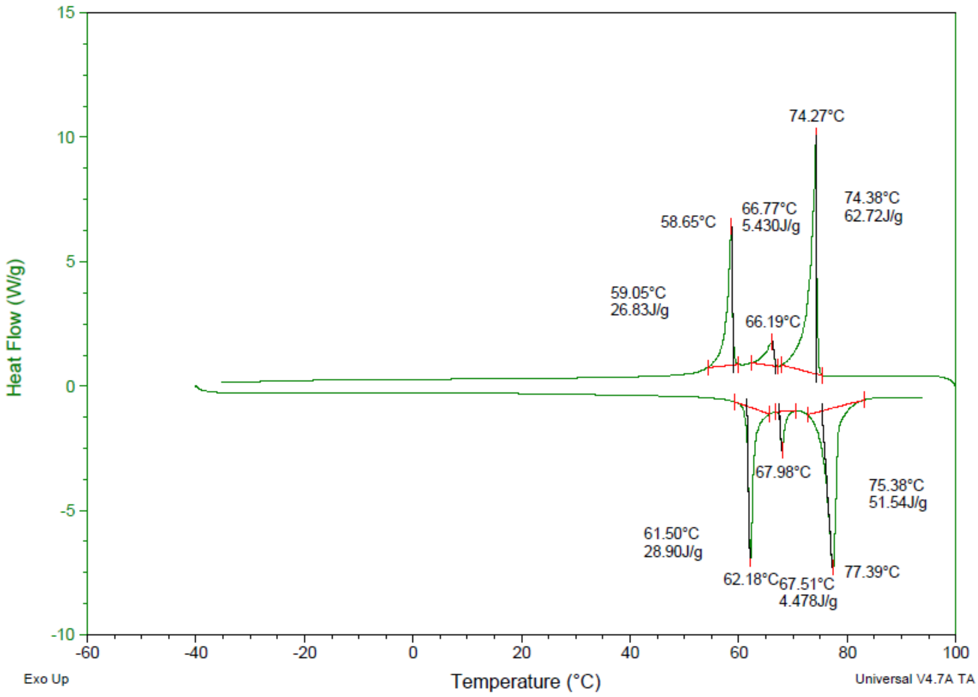

Figure S13. DSC thermogram of **Th-Oxd-Th(10)**.

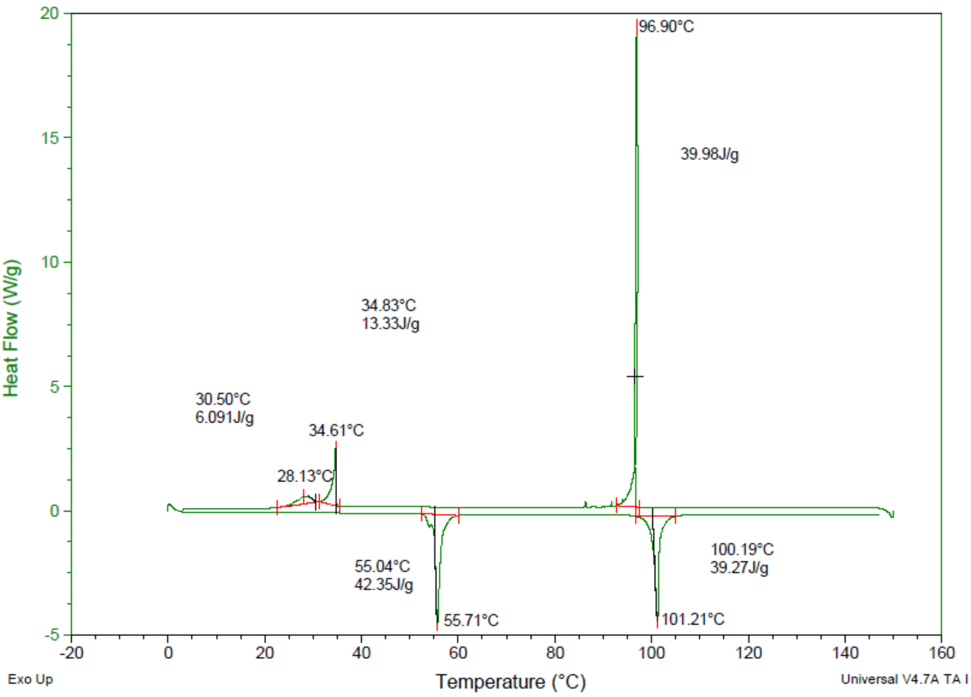

Figure S14. DSC thermogram of **Th-Thd-Th(4)**.

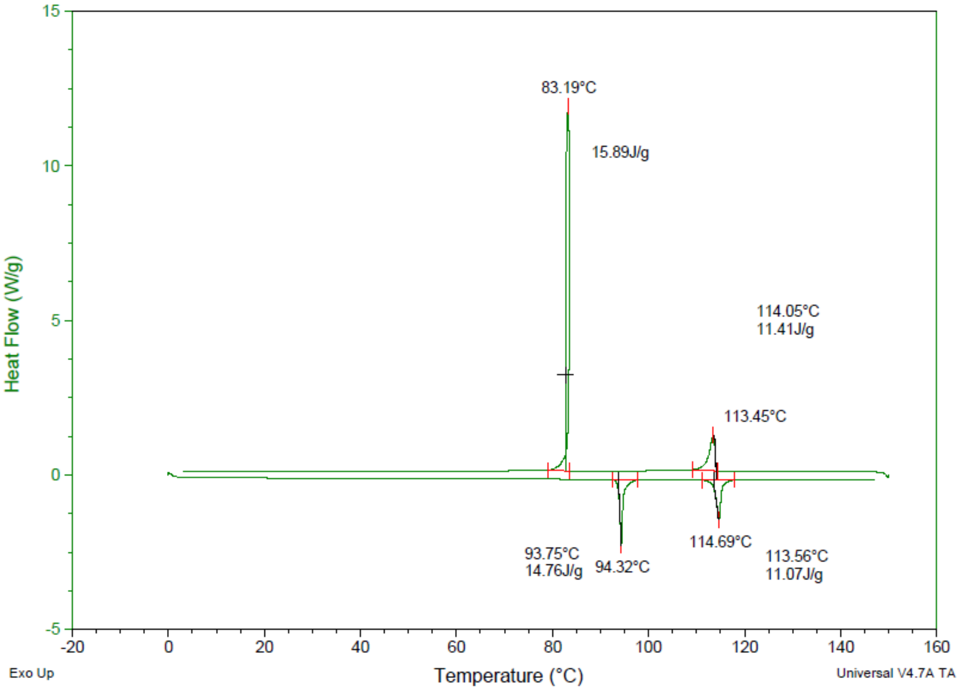

Figure S15. DSC thermogram of **Th-Thd-Th(6)**.

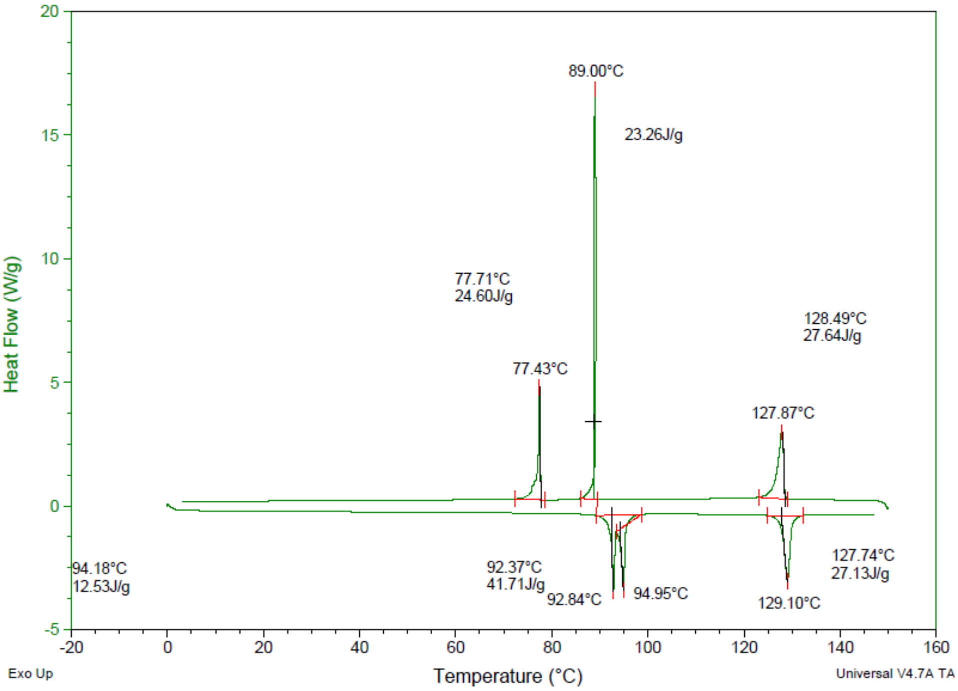

Figure S16. DSC thermogram of **Th-Thd-Th(8)**.

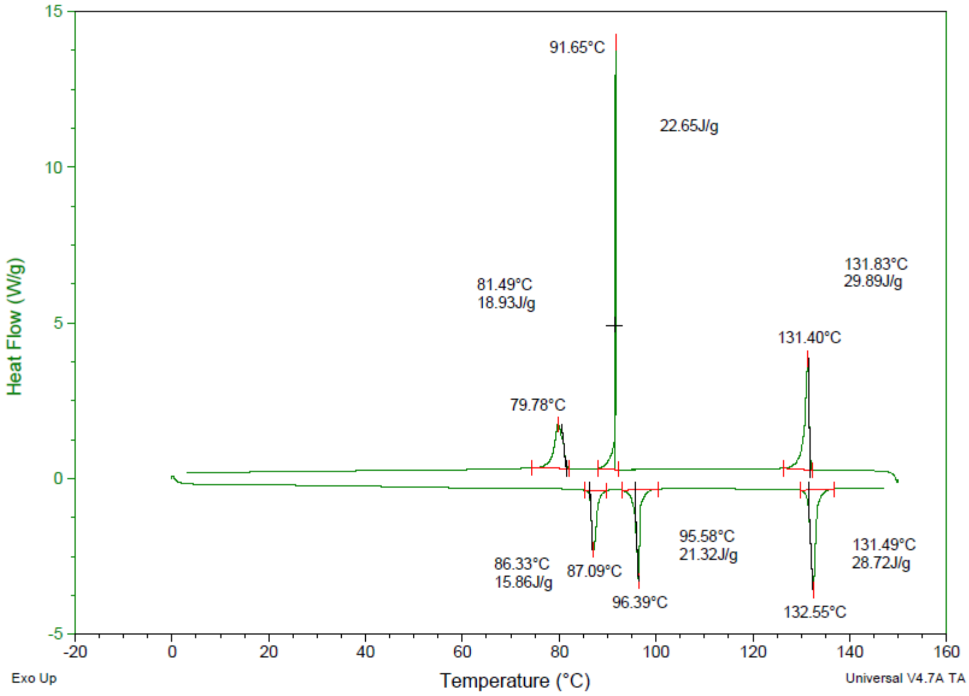

Figure S17. DSC thermogram of **Th-Thd-Th(10)**.

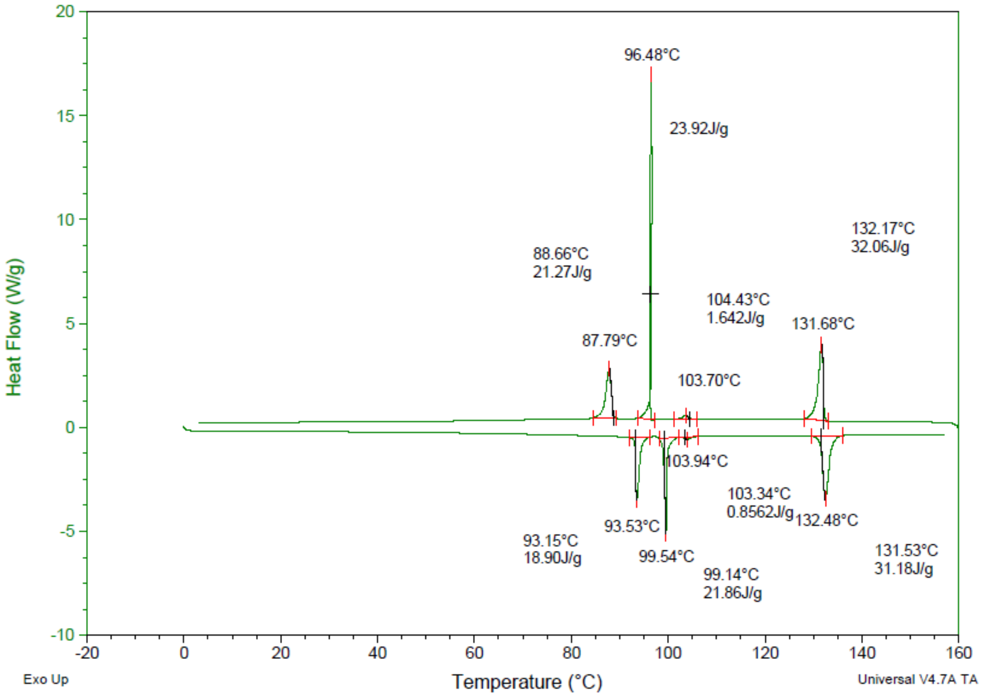

Figure S18. DSC thermogram of **Th-Thd-Th(12)**.

5. Thermogravimetric Analysis (TGA)

Thermogravimetric analysis was carried out on a Shimadzu TGA-50 with a heating rate of 2 °C per minute.

TGA of Th-Oxd-Th(10):

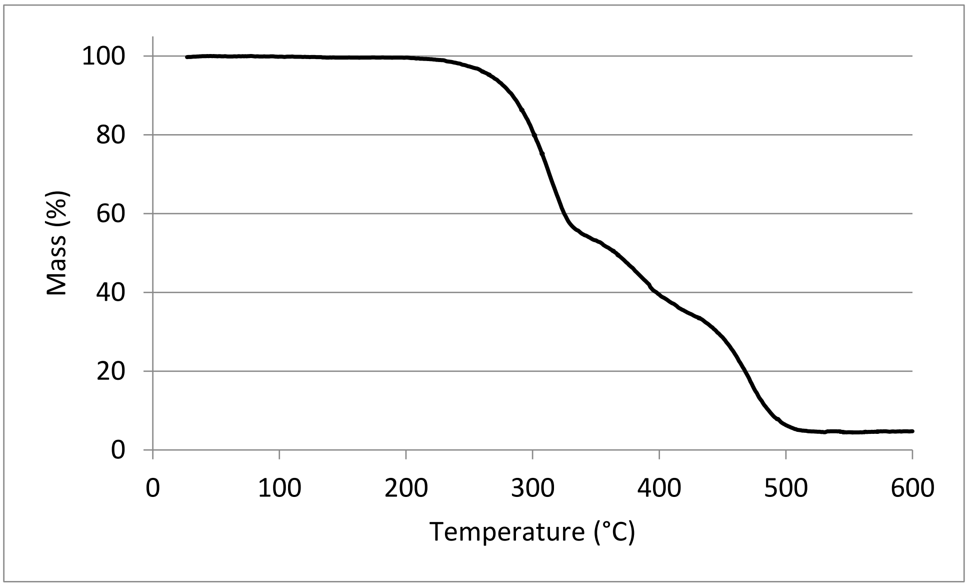

TGA of Th-Thd-Th(4):

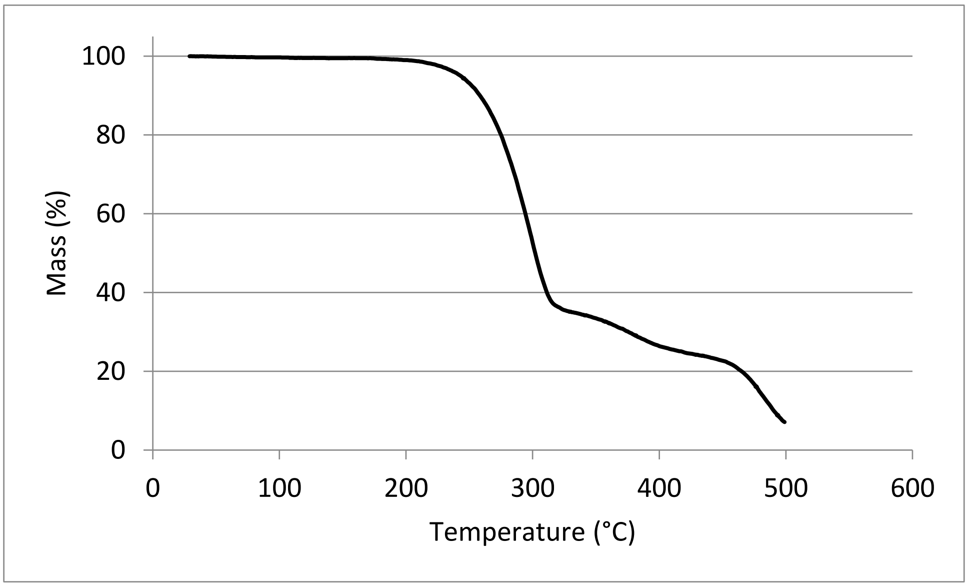

TGA of Th-Thd-Th(6):

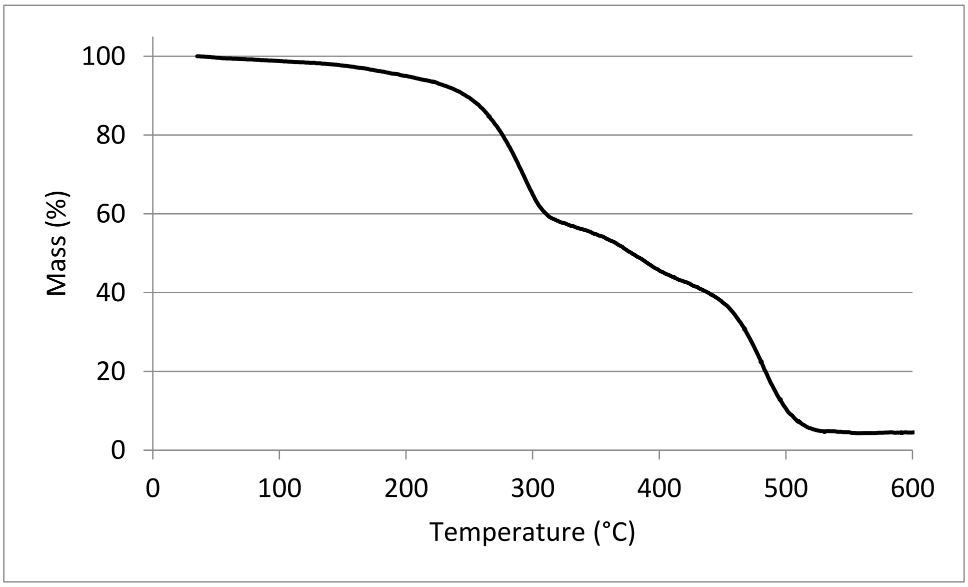

TGA of Th-Thd-Th(8):

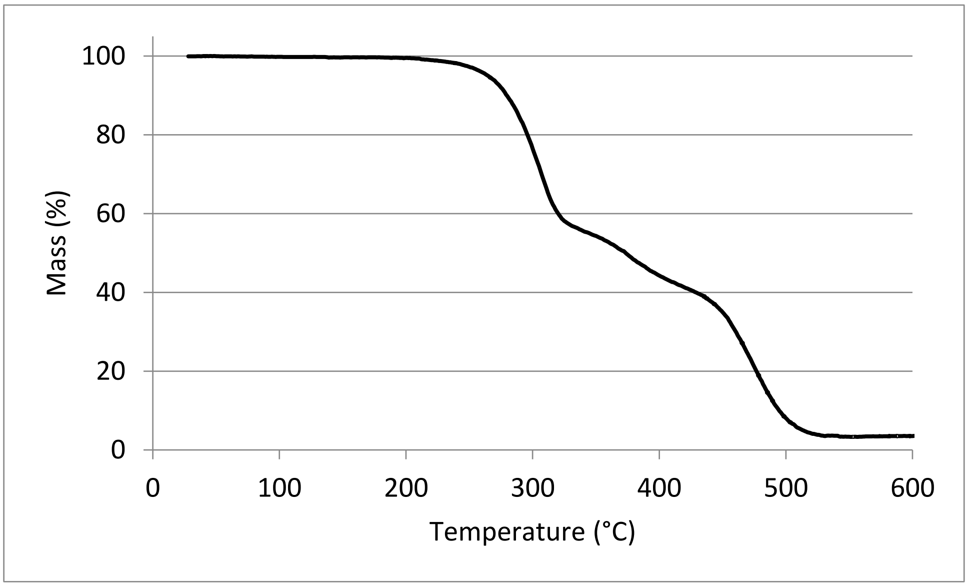

TGA of Th-Thd-Th(10):

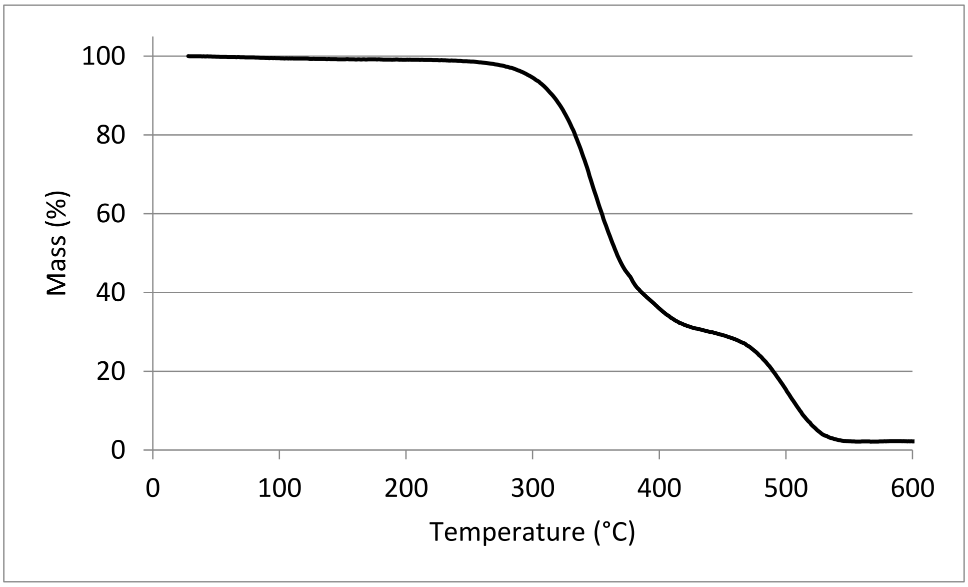

TGA of Th-Thd-Th(12):

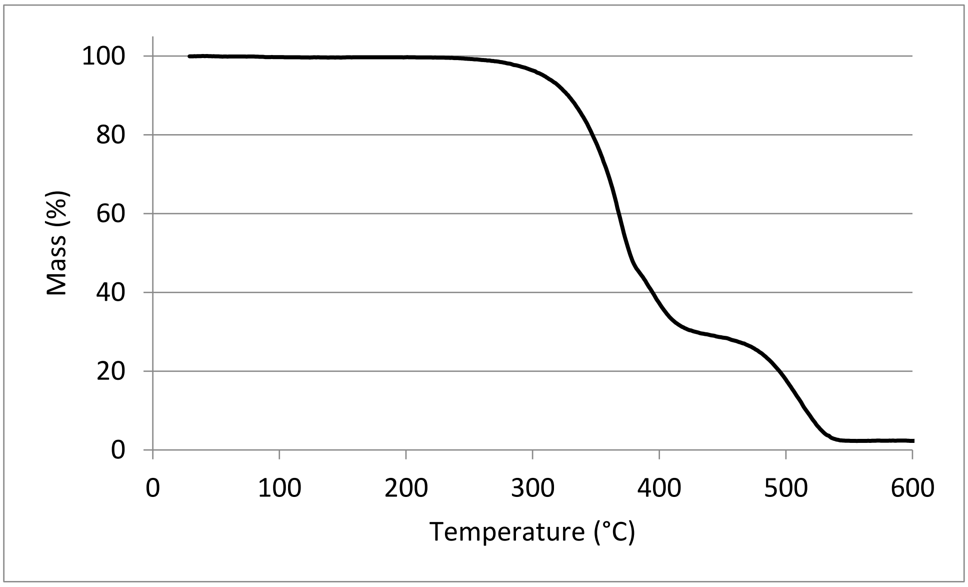

6. Single Crystal X-ray Diffraction

Suitable crystals (**Th-Thd-Th**, **Th-Thd-Th(4)** and **Th-Thd-Th(10)**) were coated in paratone oil, mounted on a MiTeGen Micro Mount, and transferred to the the X-ray diffractometer. Data was collected on a Bruker Smart instrument equipped with an APEX II CCD area detector fixed at a distance of 5.0 cm (**Th-Thd-Th**, **Th-Thd-Th(4)**) or 4.0 cm (**Th-Thd-Th(10)**), from the crystal and a Cu Kα fine focus sealed tube (λ = 1.54178 Å) operated at 1.5 kW (45 kV, 0.65 mA), filtered with a graphite monochromator. Data was collected at 150K (**Th-Thd-Th(4)**, **Th-Thd-Th(10)**) or 296K (**Th-Thd-Th**); the temperature was regulated using an Oxford Cryosystems Cryostream. Additional crystallographic information can be found in Table S4. All diffraction data were processed using the Bruker SAINT software package and were corrected for absorption effects using the multi-scan technique (SADABS [3] or TWINABS [4]). The structures were solved with direct methods (SIR92) and subsequent refinements were performed using SHELXL [5] and ShelXle. [6] Hydrogen atoms on carbon atoms were included at geometrically idealized positions (C–H bond distance 0.95Å) and were not refined. The isotropic thermal parameters of the hydrogen atoms were fixed at 1.2 times that of the preceding carbon atom. Diagrams were prepared using ORTEP-3 [7] and POV-RAY. [8] Thermal ellipsoids are shown at the 50% probability level.

Table S4. Crystallographic information table for **Th-Thd-Th**, **Th-Thd-Th(4)**, and **Th-Thd-Th(10)**.

**Compound**	**Th-Thd-Th**	**Th-Thd-Th(4)**	**Th-Thd-Th(10)**
Chemical Formula	$C_{10}H_6N_2S_3$	$C_{18}H_{22}N_2S_3$	$C_{30}H_{46}N_2S_3$
Formula Mass	250.35	362.55	530.87
a/Å	6.34810(10)	7.3146(4)	7.5511(11)
b/Å	9.63630(10)	7.6299(3)	9.6532(16)
c/Å	17.8284(2)	18.0456(9)	23.523(4)
α/°	90	85.306(4)	91.278(8)
β/°	94.9750(10)	84.800(4)	94.604(7)
γ/°	90	67.452(4)	100.468(9)
Unit cell volume/Å^3	1086.49(2)	925.09(8)	1679.5(5)
Temperature/K	159(2)	150(2)	150(2)
Space group	P21/c	P-1	P-1

Number of formula unit per cell/Z	4	2	2
Radiation type	Cu Kα	Cu Kα	Cu Kα
Absorption coefficient, μ/mm^{-1}	5.949	3.651	2.142
No. of reflections collected	5857	9288	17667
No. unique reflections	1922	3161	5526
R_{int}	0.0288	0.0693	0.0630
Final R_1 values (I>2σ(I))	0.0449	0.0867	0.2077
Final wR(F^2) values (I>2σ(I))	0.1299	0.2800	0.5868
Final R_1 values (all data)	0.0475	0.1088	0.2178
Final wR(F^2) (all data)	0.1331	0.2965	0.6060
Goodness of fit	1.080	1.161	3.232

All crystal structure figures below follow the legend: sulphur in yellow, nitrogen in light blue, carbon in grey, hydrogen in white.

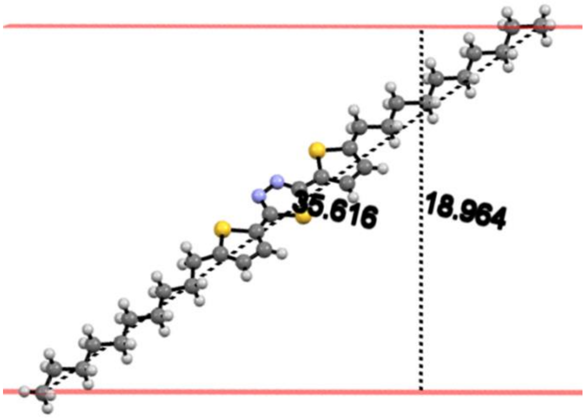

Figure S19. Estimation of the tilt angle in the room temperature smectic phase of **Th-Thd-Th(10)** based on the single crystal structure.

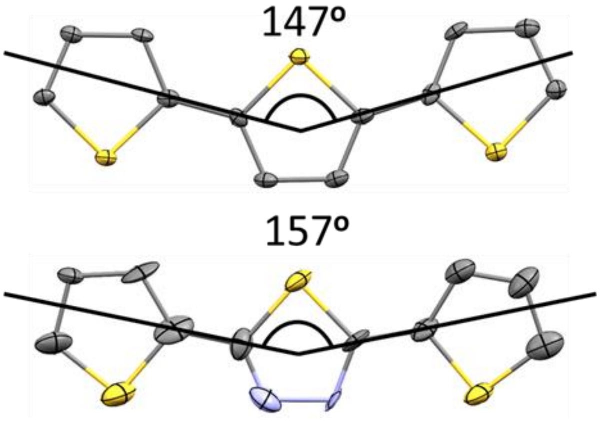

Figure S20. Bond angles at the 2 and 5 positions of the central heterocycle for **Th$_3$(8)**[9] and **Th-Thd-Th(10)**. Alkyl chains and hydrogens omitted for clarity.

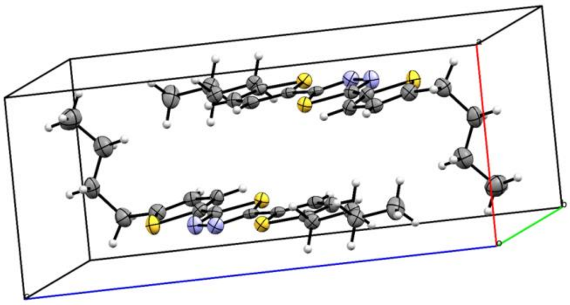

(a)

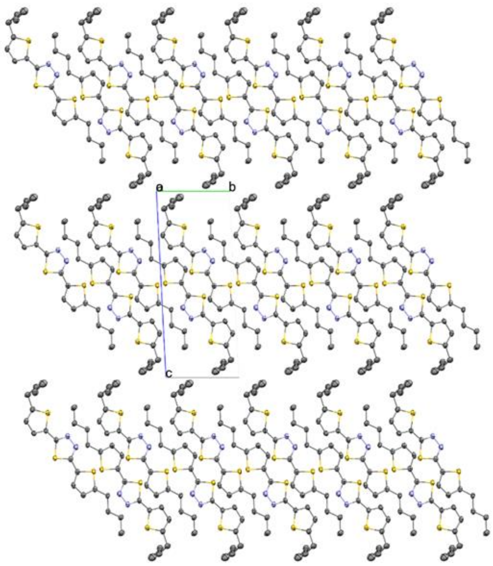

(b)

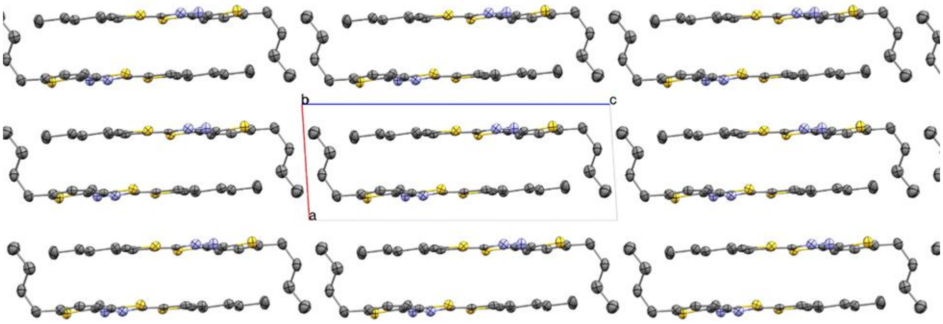

(c)

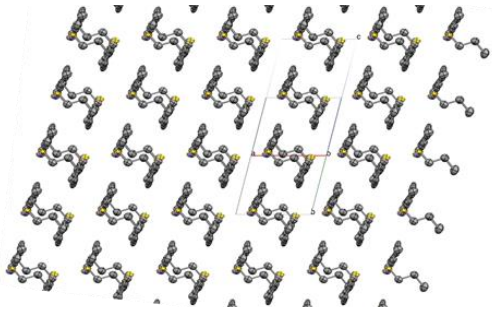

(d)

Figure S21. Single crystal structure of **Th-Thd-Th(4)** showing the unit cell (a), lamellar order looking down the a- or b- axis (b and c respectively), and intra-layer packing looking down the c-axis (d).

a)

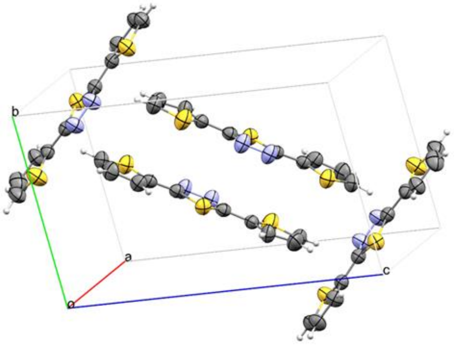

b)

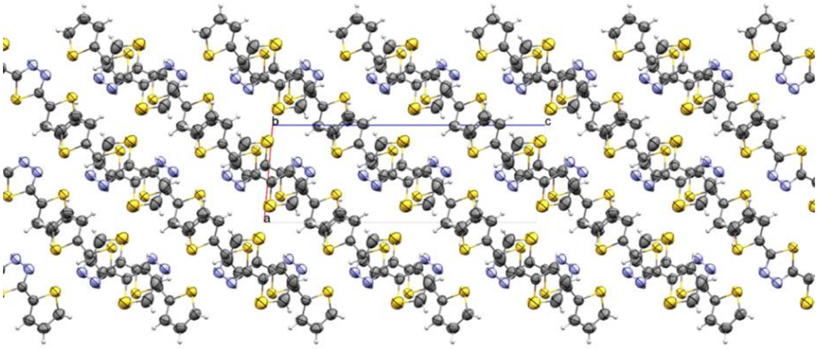

c)

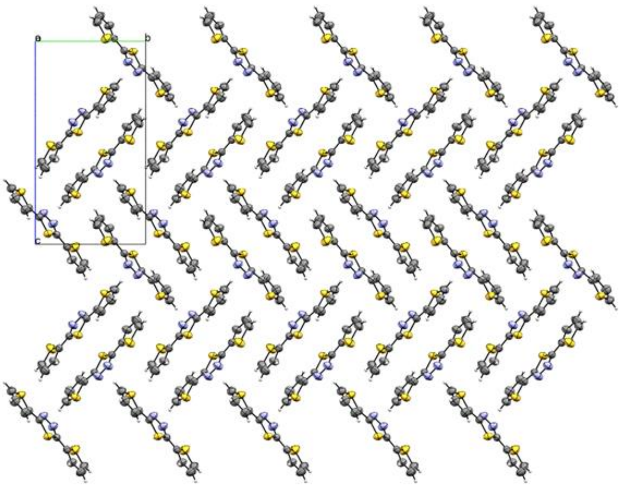

Figure S22. Single crystal structures of **Th-Thd-Th** showing the unit cell (a), order looking down the b-axis (b), and herringbone packing looking down the a-axis (c).

a)

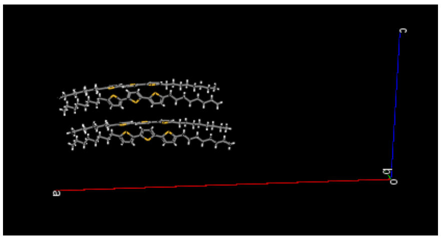

b)

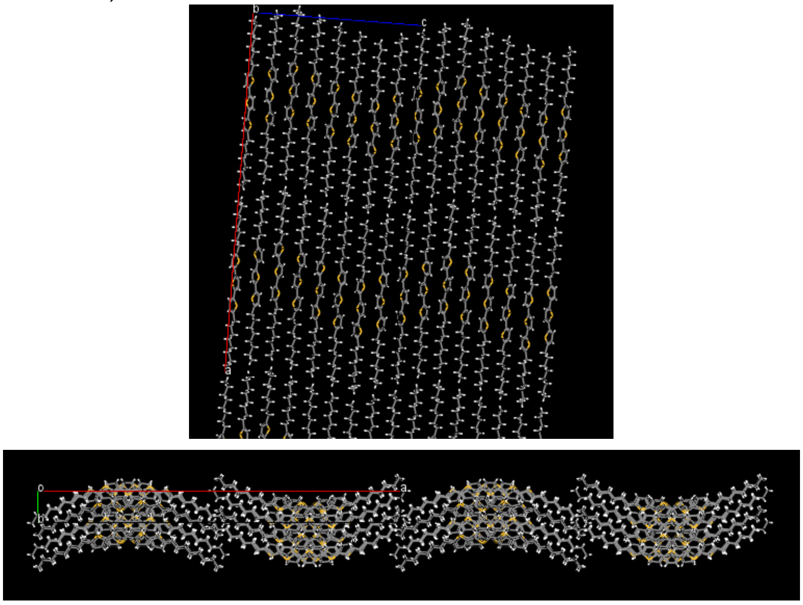

c)

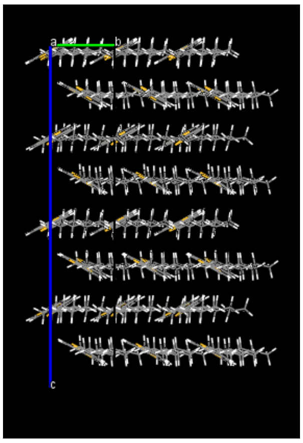

Figure S23. Literature single crystal structures of **Th$_3$(8)**[9] showing the unit cell (a), lamellar order (b), and intra-layer packing (c).

a)

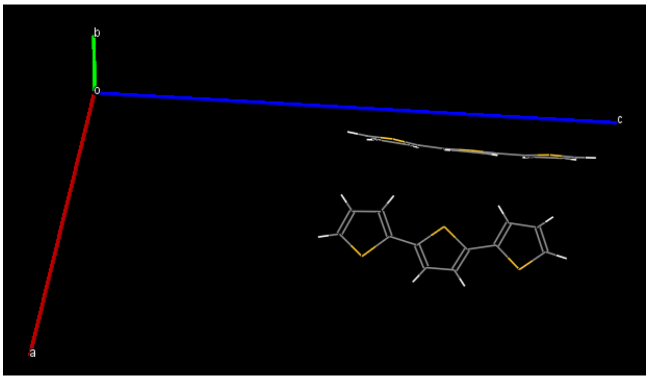

b)

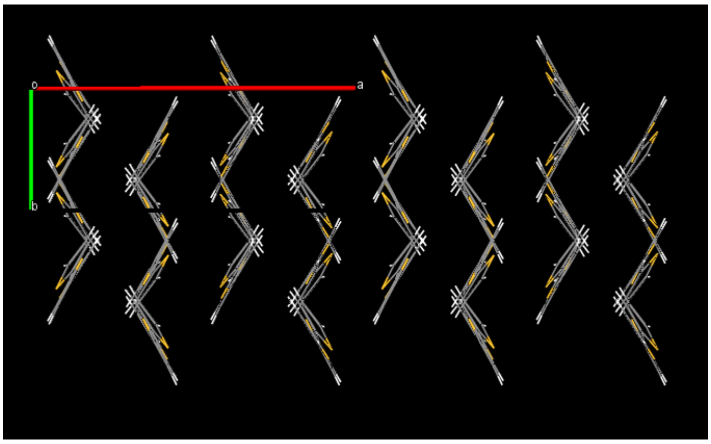

Figure S24. Literature single crystal structures of **Th**$_3$[10] showing the unit cell (a) and herringbone packing (b).

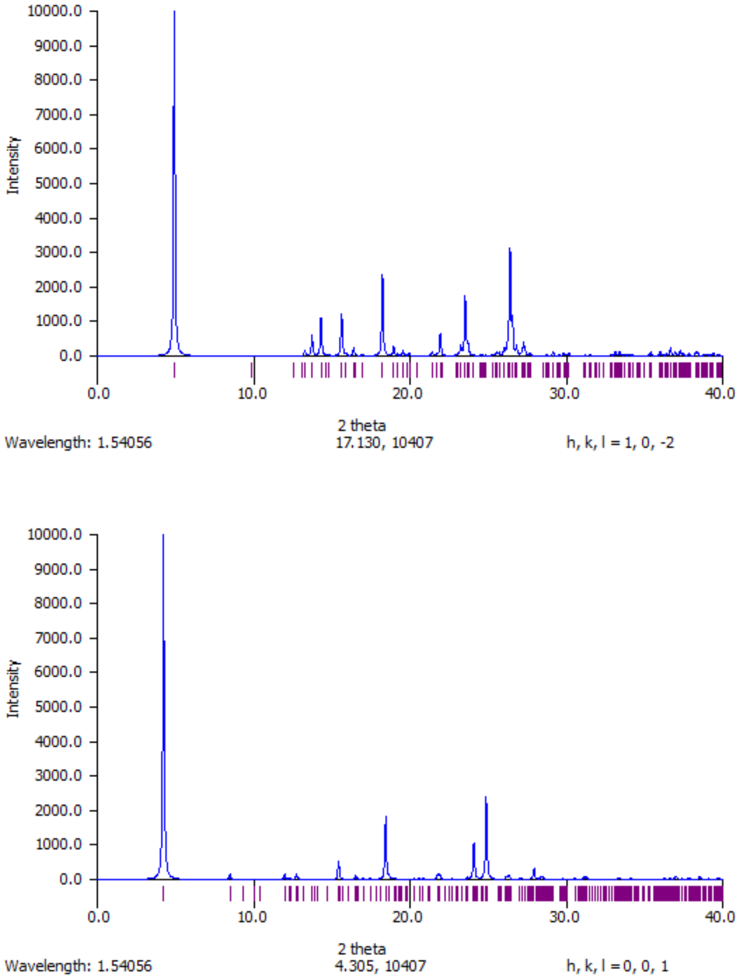

Figure S25. Simulated powder XRD patterns of **Th-Thd-Th(4)** (a) and **Th-Thd-Th(10)** (b) as determined based on the single crystal structure.

7. UV/vis Absorption Spectroscopy

Table S5. Summary of UV-Vis spectral properties.

Compound	λ_{abs}^{max} (nm)	ε (M^{-1}cm^{-1})	λ_{abs}^{onset} (nm)	E_g^{opt} (eV)[a]

**Th-Oxd-Th(10)**	328	28200	371	3.35
**Th-Thd-Th(10)**	359	26800	409	3.04
**Th₃(10)**	367	25900	423	2.93

[a]Optical bandgap, estimated by $E_g = hc/\lambda_{abs}^{onset}$

a)

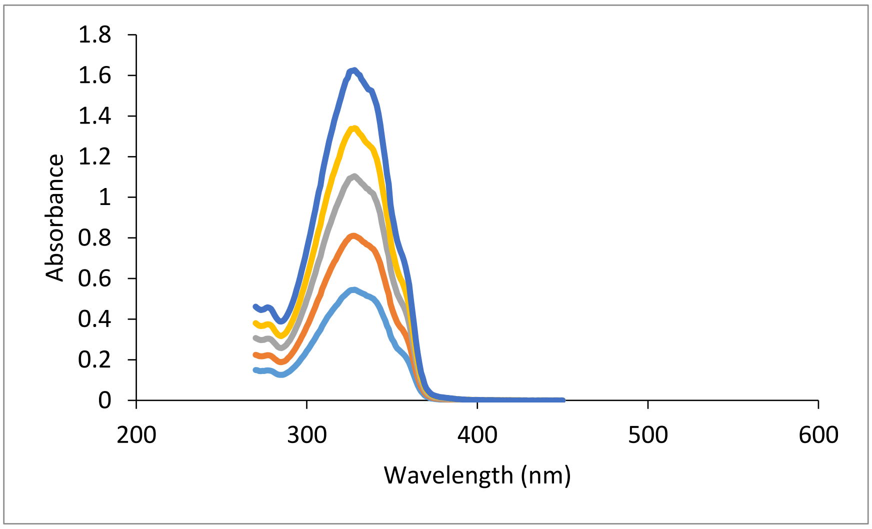

b)

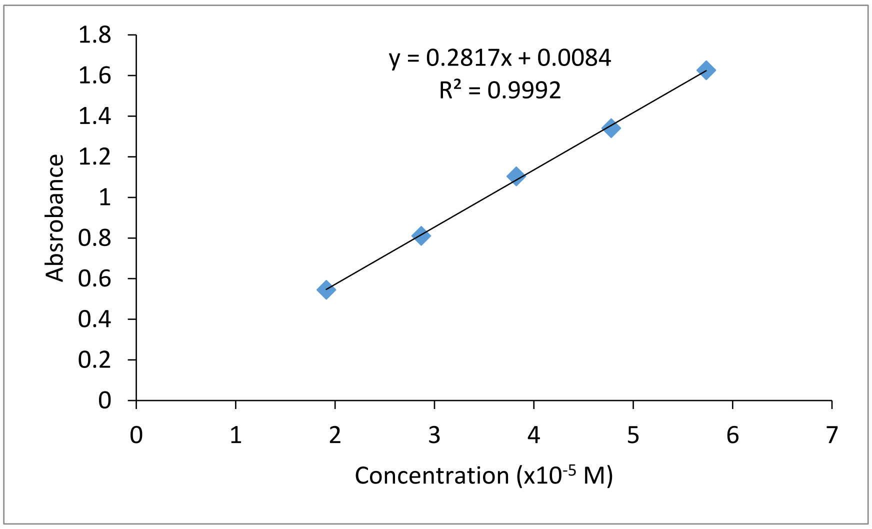

Figure S26. Absorption spectra of **Th-Oxd-Th(10)** in chloroform at various concentrations ranging from around 6x10^{-5} to 2x10^{-5} M (top to bottom) (a) and the resulting Beer-Lambert plot (b).

a)

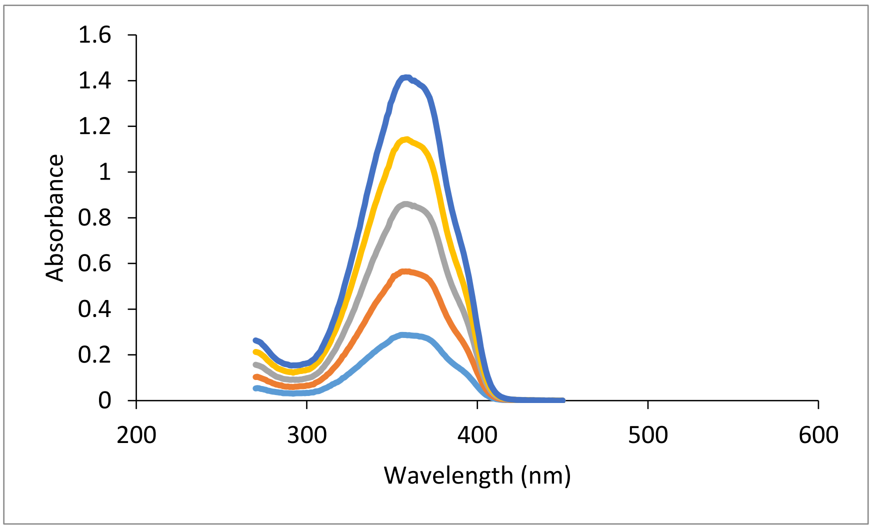

b)

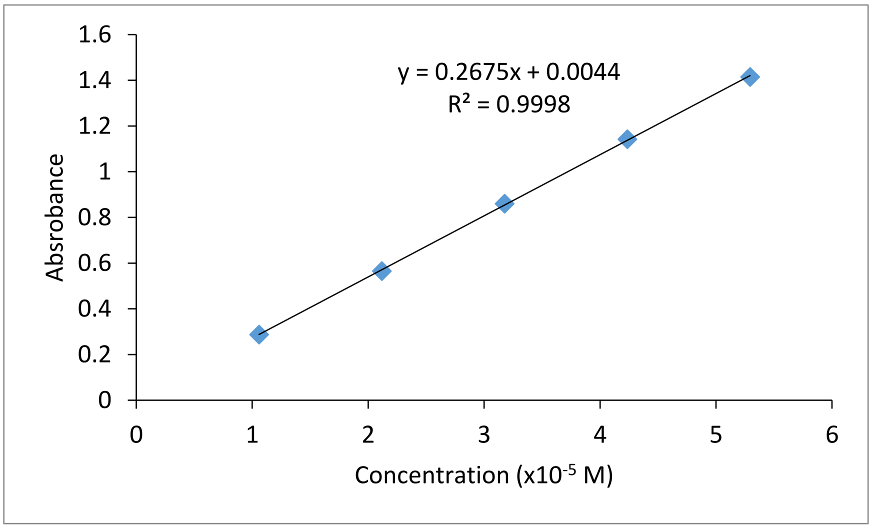

236 **Figure S27.** Absorption spectra of **Th-Thd-Th(10)** in chloroform at various concentrations ranging
237 from around 5 × 10^{-5} to 1 × 10^{-5} M (top to bottom) (a) and the resulting Beer-Lambert plot (b).

a)

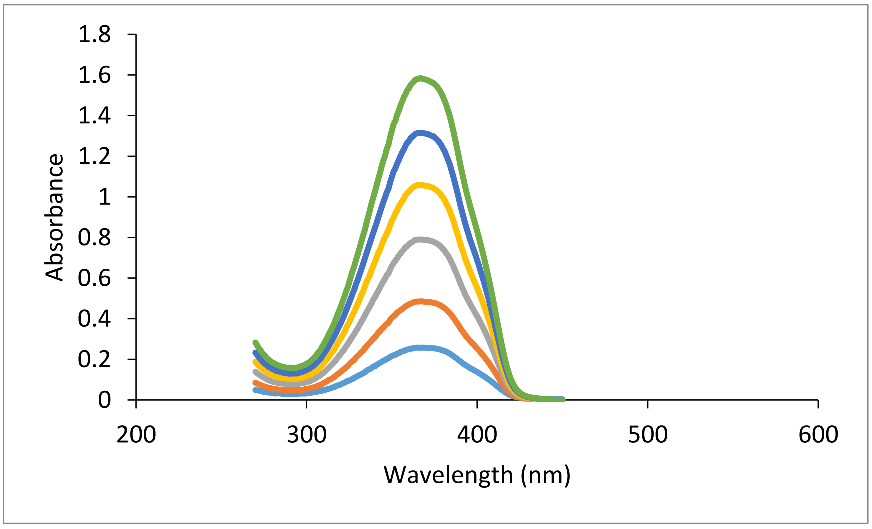

b)

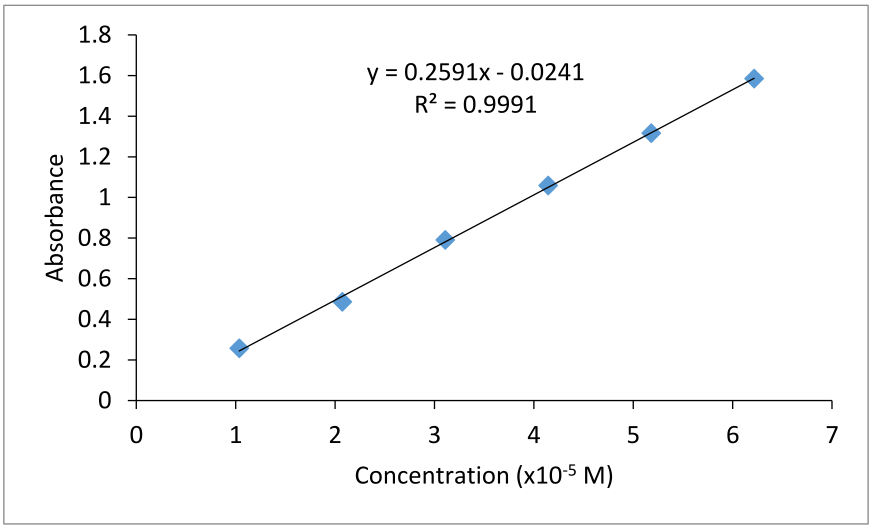

Figure S28. Absorption spectra of **Th_3(10)** in chloroform at various concentrations ranging from around 6 × 10^{-5} to 1 × 10^{-5} M (top to bottom) (a) and the resulting Beer-Lambert plot (b).

8. Fluorescence Excitation/Emission Spectroscopy

Table S6. Summary of fluorescence spectral properties.

**Compound**	λ_{ex}^{max} **(nm)**[a]	λ_{em}^{max} **(nm)**[b]	**Stokes shift (nm)**	Φ_f[c]
**Th-Oxd-Th(10)**	340	387	47	0.48
**Th-Thd-Th(10)**	369	430	61	0.49
**Th_3(10)**	380	443	63	0.56

[a] Observed at the corresponding λ_{em}^{max} ; [b] Measured at the respective λ_{ex}^{max} ; [c] Relative to quinine sulfate standard (Φ_f = 0.54) in 0.1 M H_2SO_4

Table S7. Detailed fluorescence data used for quantum yield determination.

**Compound**	**Concentration ($x10^{-6}$ M)**	**Maximum absorbance, A_{max}**	**Integrated emission intensity, I_{em}**	**I_{em}/A_{max} gradient**[a]	Φ_f[b]
**Th-Oxd-Th(10)**	0.96	552038	43751369	74.9	0.48
	1.91	898329	69736325		
	2.87	1235771	94299072		
	3.82	1567427	119646050		
	4.78	1915589	145914659		
**Th-Thd-Th(10)**	2.12	275332	21930717	76.4	0.49
	6.35	559616	43049313		

	8.47	696109	53070730		
	10.59	828772	63336304		
	12.70	983655	75462409		
	16.94	1258332	97024851		
	0.52	376360	38399486		
	0.62	423623	42422719		
**Th_3(10)**	0.73	526075	51875681	87.1	0.56
	0.83	599034	58557104		
	0.93	685502	64905104		
	6.65	622805	59563561		
	8.87	792582	76626907		
	11.09	985531	93600171		
Quinine sulfate	13.30	1151426	110217780	98.2	0.54
	15.52	1305979	126343550		
	17.74	1459479	142261748		

[a] Determined from slope of I_{em} vs. A_{max} at various concentrations, see plots below; [b] Calculated according to: $\Phi_{f,x} = \Phi_{f,ref}\ (I_{em,x}/A_{max,x})\ (A_{max,ref}/I_{em,ref})\ (\eta_x/\eta_{ref})^2$,[11] using the quinine sulfate standard ($\Phi_f = 0.54$)[12] in 0.1 M H_2SO_4, the gradients above determined from experiment, and $\eta_{chloroform} = 1.444$ and $\eta_{water} = 1.333$.

a)

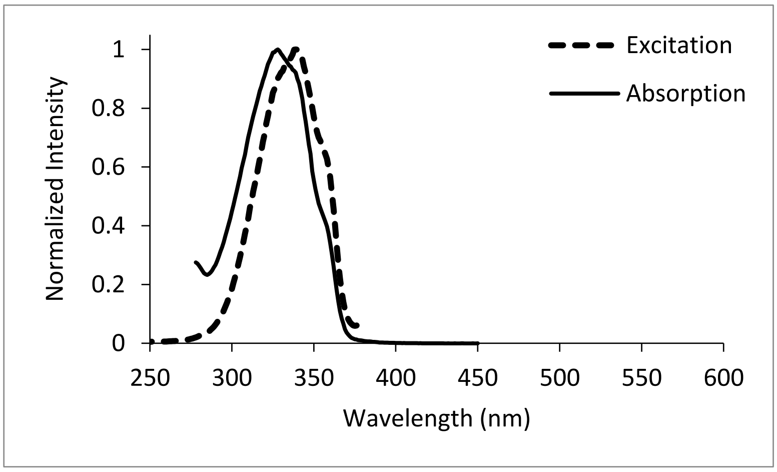

b)

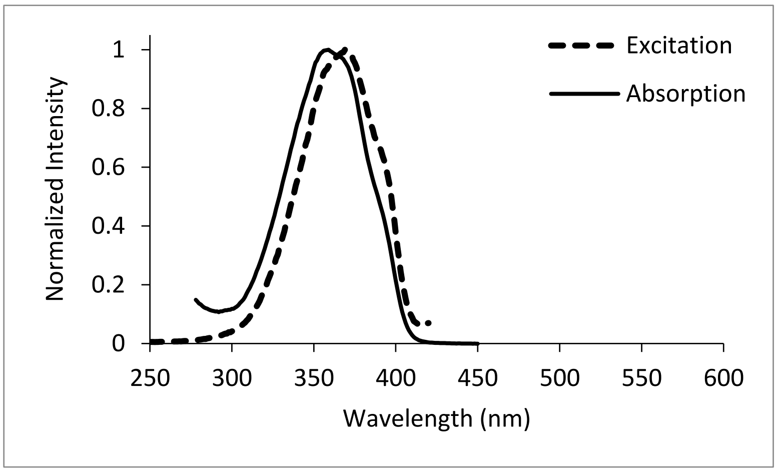

c)

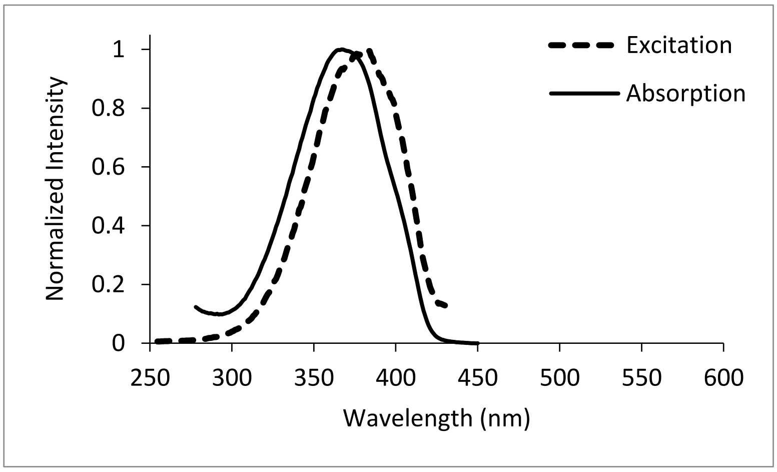

Figure S29. Overlay of UV/vis absorption (solid line) and fluorescence excitation (dashed line) spectra of **Th-Oxd-Th(10)** (a), **Th-Thd-Th(10)** (b), and **Th$_3$(10)** (c) in $CHCl_3$.

a) b)

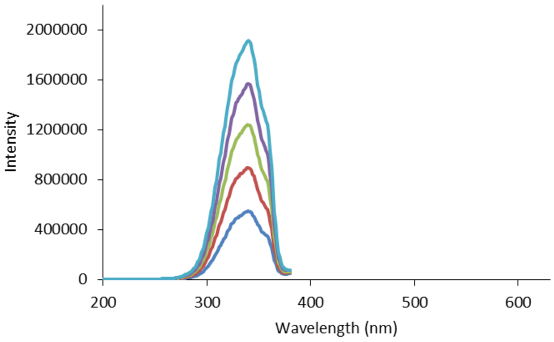

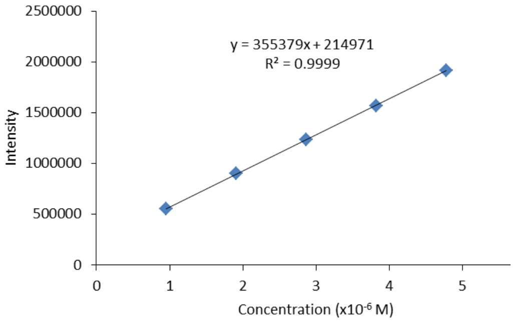

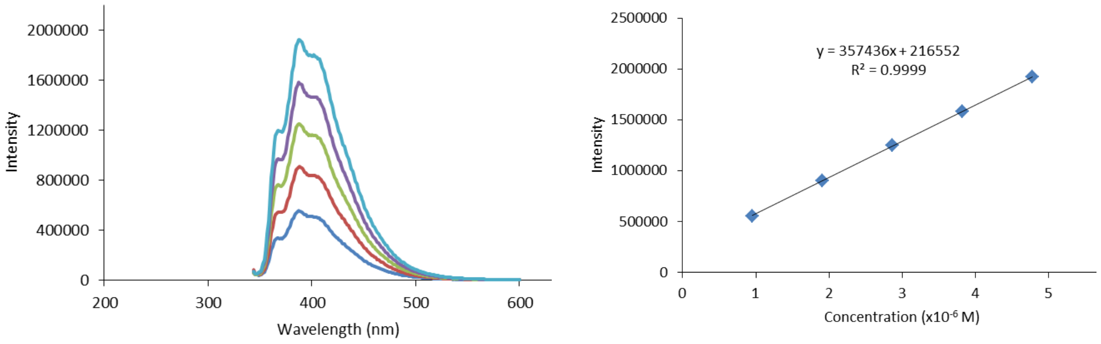

Figure S30. Fluorescence excitation (top) and emission (bottom) spectra of **Th-Oxd-Th(10)** in chloroform at various concentrations ranging from around 5 x 10^{-6} to 1x10^{-6} M (shown in descending order) (a) and the respective Beer-Lambert plots demonstrating the linear range (b).

a) b)

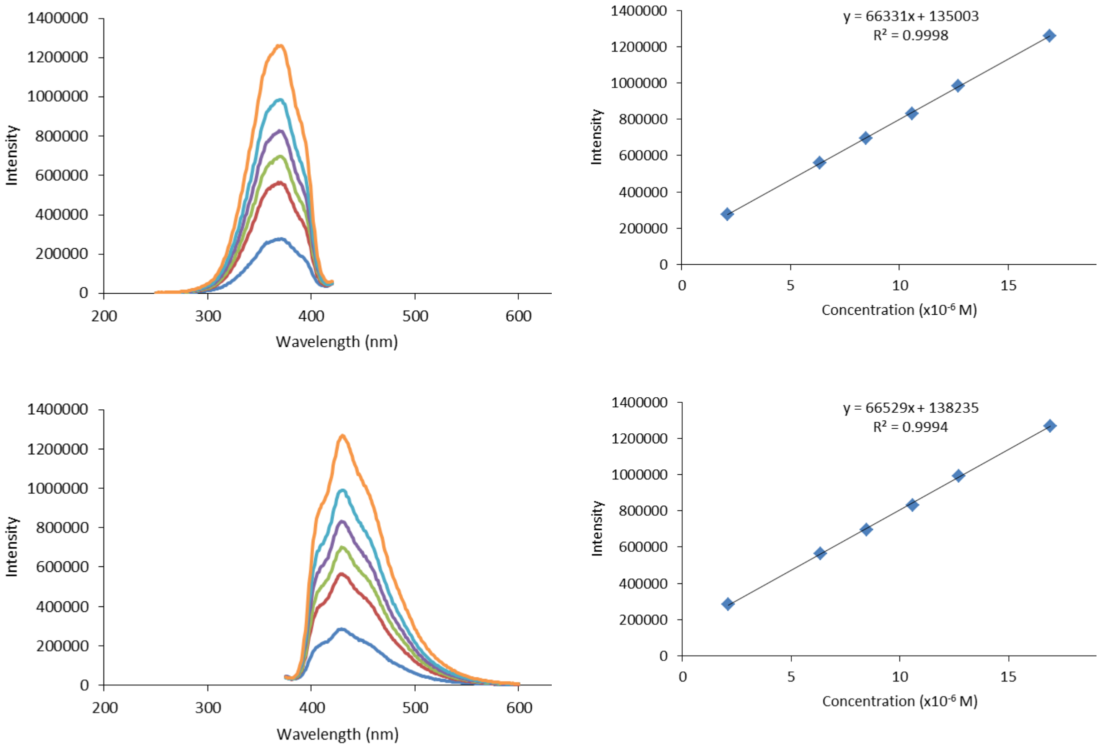

Figure S31. Fluorescence excitation (top) and emission (bottom) spectra of **Th-Thd-Th(10)** in chloroform at various concentrations ranging from around 16 × 10^{-6} to 2 × 10^{-6} M (shown in descending order) (a) and the respective Beer-Lambert plots demonstrating the linear range (b).

a) b)

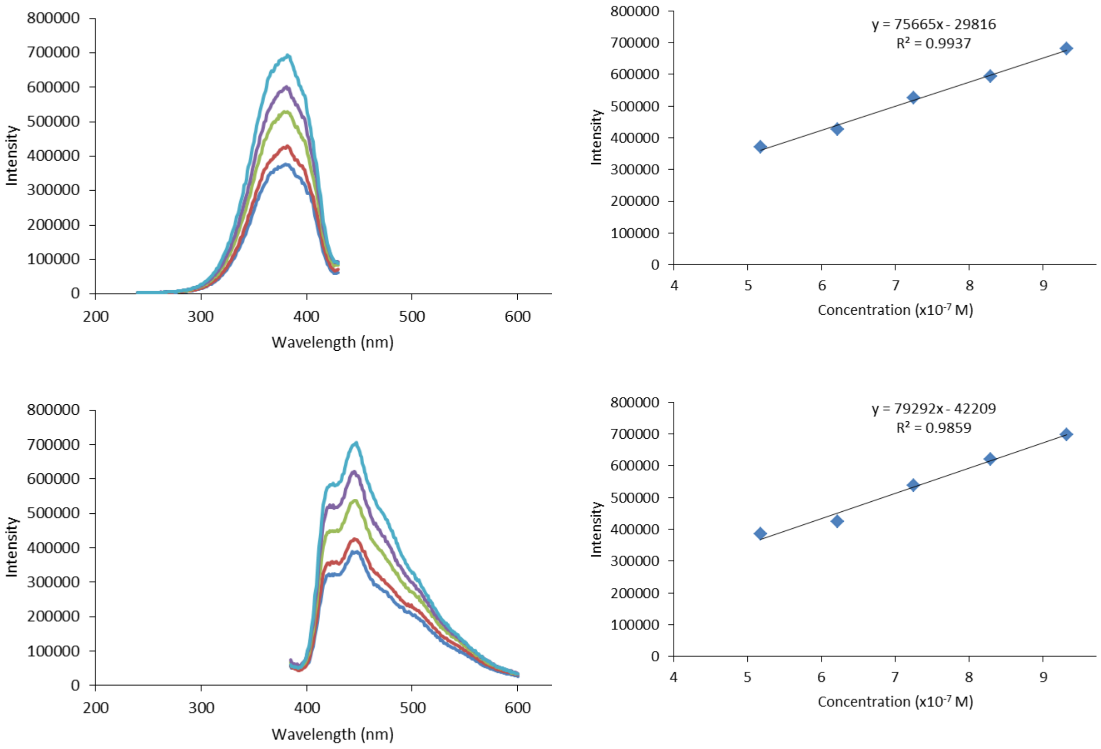

Figure S32. Fluorescence excitation (top) and emission (bottom) spectra of **Th3(10)** in chloroform at various concentrations ranging from around 9 × 10-7 to 5 × 10-7 M (shown in descending order) (a) and the respective Beer-Lambert plots demonstrating the linear range (b).

a) b)

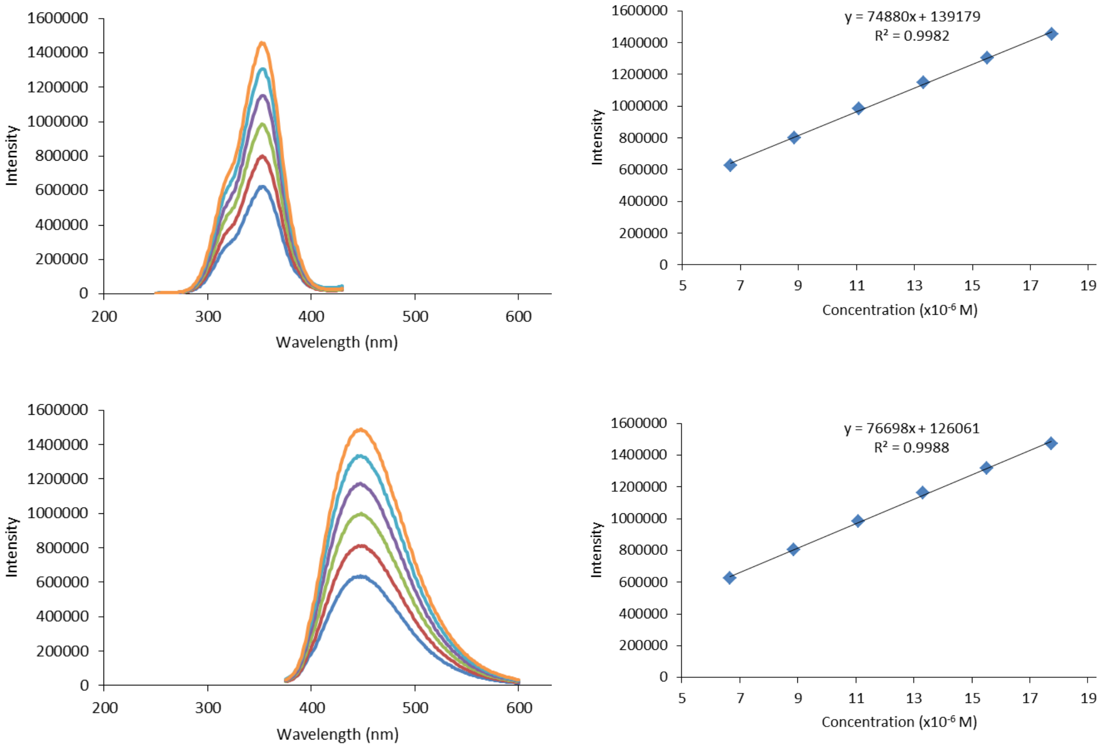

Figure S33. Fluorescence excitation (top) and emission (bottom) spectra of quinine sulfate in 0.1 M sulfuric acid at various concentrations ranging from around 17x10-6 to 6x10-6 M (shown in descending order) (a) and the respective Beer-Lambert plots demonstrating the linear range (b).

a)

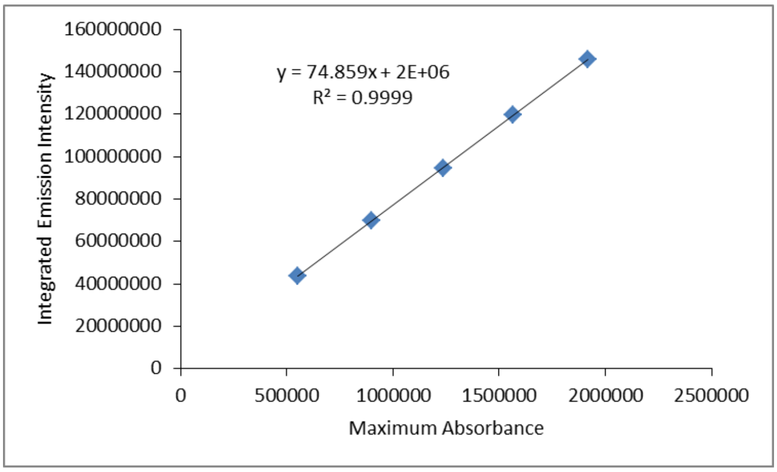
y = 74.859x + 2E+06
R² = 0.9999
Integrated Emission Intensity
Maximum Absorbance
0
20000000
40000000
60000000
80000000
100000000
120000000
140000000
160000000
0
500000
1000000
1500000
2000000
2500000

b)

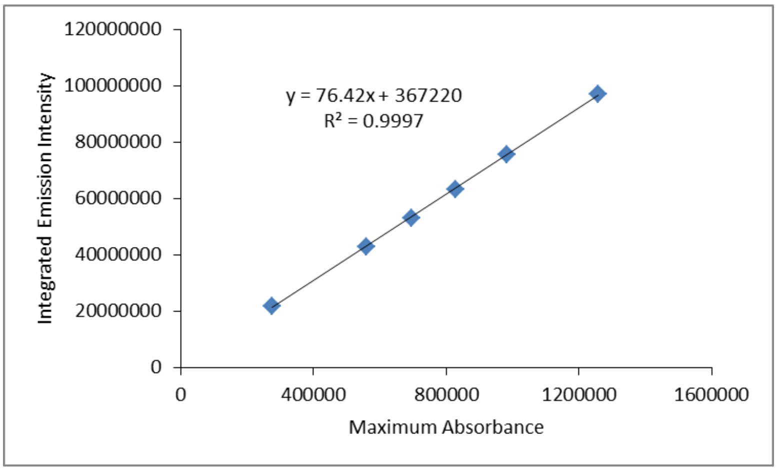
y = 76.42x + 367220
R² = 0.9997
Integrated Emission Intensity
Maximum Absorbance
0
20000000
40000000
60000000
80000000
100000000
120000000
0
400000
800000
1200000
1600000

c)

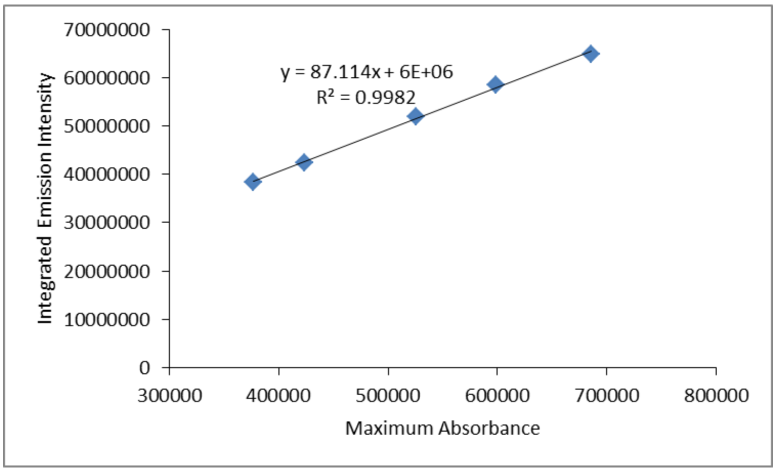
y = 87.114x + 6E+06
R² = 0.9982
Integrated Emission Intensity
Maximum Absorbance
0
10000000
20000000
30000000
40000000
50000000
60000000
70000000
300000
400000
500000
600000
700000
800000

d)

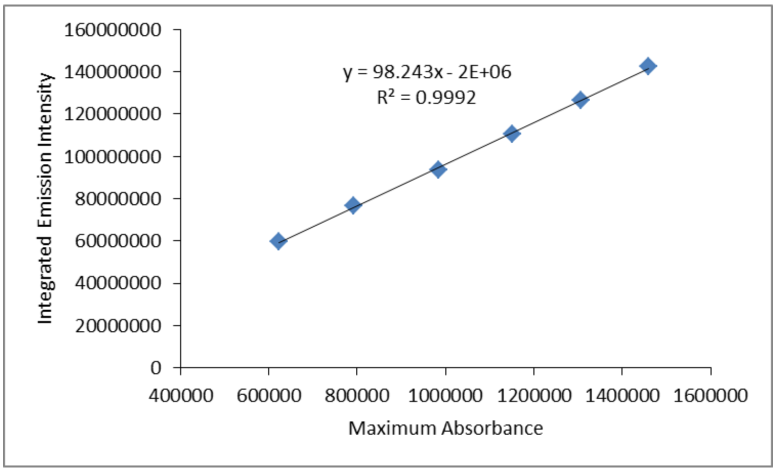

Figure S34. Integrated emission intensity vs. maximum absorbance plots created based on the fluorescence spectra at various concentrations shown above for **Th-Oxd-Th(10)** (a), **Th-Thd-Th(10)** (b), **Th$_3$(10)** (c), and quinine sulfate (d).

9. Cyclic Voltammetry (CV)

Cyclic voltammetry scans were performed on a PAR-263A potentiometer. All analytes were scanned as 1.0 mM solutions in THF (**Th-Ox-Th(10)** and **Th-Thd-Th(6)** reduction) or MeCN (**Th-Thd-Th(6)** oxidation) with a 0.1 M tetrabutylammonium perchlorate supporting electrolyte. The apparatus was a 20 ml glass cell equipped with a glassy carbon working electrode, platinum counter electrode and silver wire reference electrode. All scans were reference internally to a Fc/Fc$^+$ reversible redox couple.

Table S8. Summary of the raw CV data for select compounds.

Compound	Process/ solvent	Analyte (compound) E_a (V)	Analyte (compound) E_c (V)	Analyte (compound) $E_{1/2}$ (V)[a]	Ferrocene reference E_a (V)	Ferrocene reference E_c (V)	Ferrocene reference $E_{1/2}$ (V)
**Th-Oxd-Th(10)**	Reduction/ THF	n/a, irreversible		-2.20	1.29	0.53	0.91
**Th-Thd-Th(6)**	Reduction/ THF	-1.32	-1.64	-1.48	1.35	0.50	0.93
	Oxidation/ MeCN	n/a, irreversible		1.92	0.92	0.56	0.74

[a] Irreversible processes estimated based on middle of onset slope

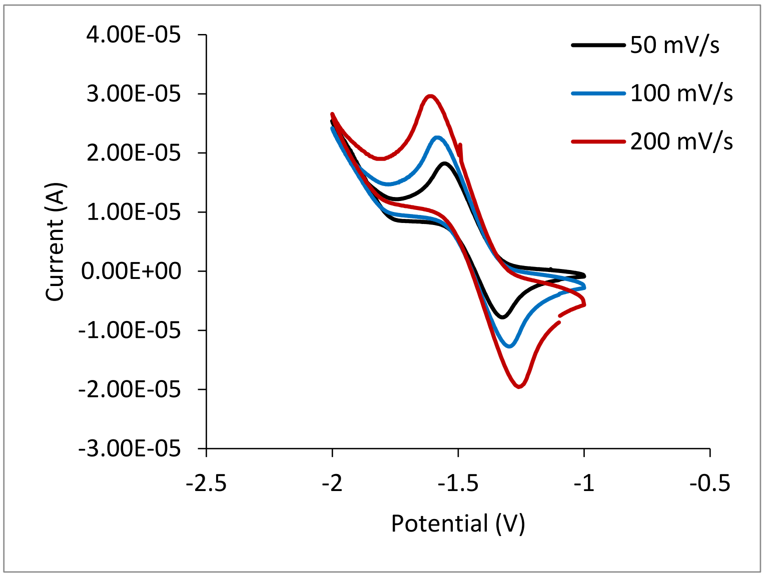

Figure S35. Unprocessed cyclic voltammograms showing the reversible reduction of **Th-Thd-Th(6)** at different scan rates.

a)

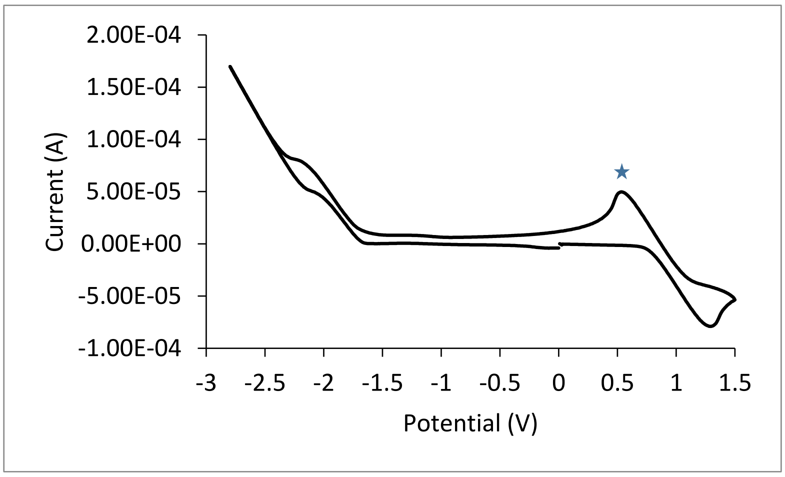

b)

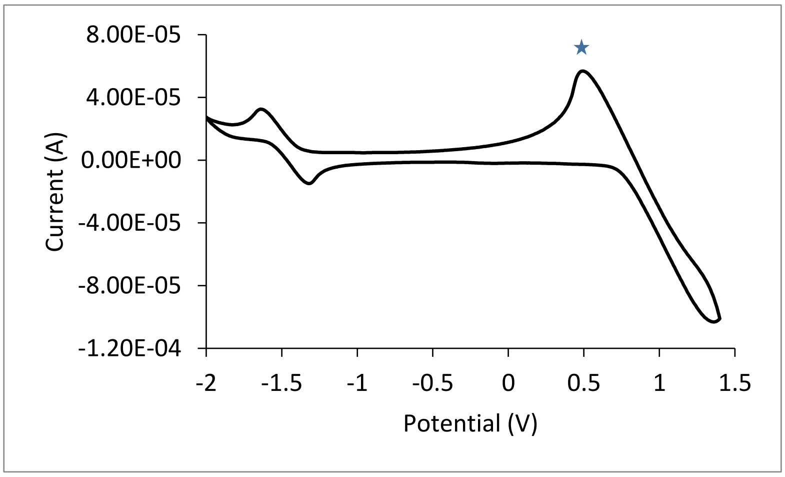

c)

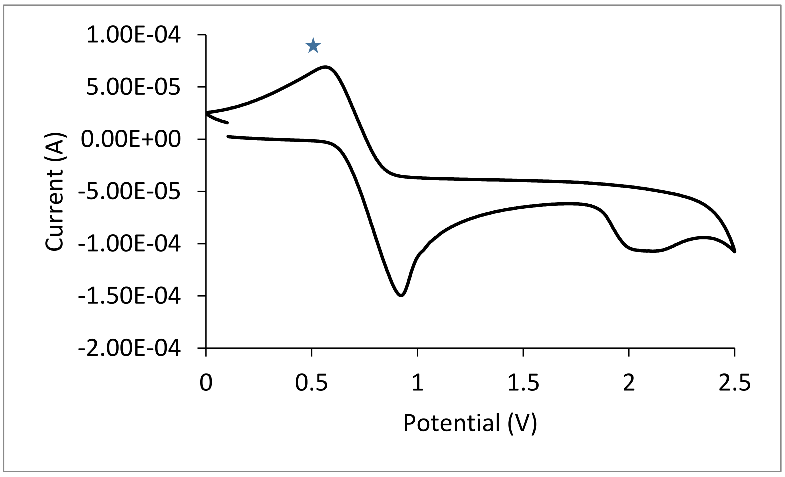

Figure S36. Unprocessed cyclic voltammograms showing the ferrocene internal standard signal (blue star) used for reference for **Th-Oxd-Th(10)** reduction in THF (a), **Th-Thd-Th(6)** reduction in THF (b), and **Th-Thd-Th(6)** oxidation in MeCN (c).

10. DFT and TD-DFT Calculations

Density functional theory (DFT) – DFT calculations were carried out using B3LYP functional[13,14] and 6-31G* basis set (C, H, N, O, S)[15–18] in Gaussian 16[19] to determine the optimized molecular structures (Figure 6) as well as the frontier molecular orbitals and respective HOMO/LUMO energy levels (Figure S36). All of these results were consistent with those obtained by different basis sets used during TD-DFT calculations (see following).

Time-dependent density functional theory (TD-DFT) – Geometry optimization calculations were completed using the Gaussian 16 program (Revision B.01)[19], the restricted B3LYP functional[13,14], the 6-31G* basis set (C, H, N, O, S), with a polarized continuum model (PCM) for $CHCl_3$ (dielectric ε = 4.9).[15–18] Frequency calculations at the same level of theory confirmed that the optimized structures were located at a minimum on the potential energy surface. Single-point calculations were done using restricted B3LYP functional, the TZVP basis set (C, H, N, O, S)[20,21], with a PCM for $CHCl_3$. The intensities of 30 lowest energy transitions using TD-DFT[22,23] calculations were performed using the CAM-B3LYP functional[24–26], the TZVP basis set (C, H, N, O, S), with a PCM for $CHCl_3$.

Table S9. Summary of predicted properties determined by DFT (B3LYP/6-31G*).

Compound	Gas phase			Solvated in THF		
	HOMO (eV)	LUMO (eV)	E_g^{calc} (eV)	HOMO (eV)	LUMO (eV)	E_g^{calc} (eV)
**Th_3(10)**	-4.88	-1.51	3.37	-5.05	-1.69	3.36
**Th-Oxd-Th(10)**	-5.59	-1.62	3.97	-5.77	-1.80	3.97
**Th-Thd-Th(10)**	-5.54	-1.90	3.64	-5.71	-2.08	3.62
**Th-Thd-Th(6)**	-5.54	-1.90	3.64	-5.71	-2.09	3.62

Table S10. Summary of predicted properties determined by TD-DFT (CAM-B3LYP/TZVP).

Compound	TD-DFT

	λ_{abs}^{max} (nm)	f[a]	E_g^{opt} (eV)[b]
**Th_3(10)**	383	1.15	2.67
**Th-Oxd-Th(10)**	323	1.16	3.26
**Th-Thd-Th(10)**	354	1.18	2.94

[a] Oscillator strength

[b] Optical bandgap, estimated by $E_g = hc/\lambda_{abs}^{onset}$

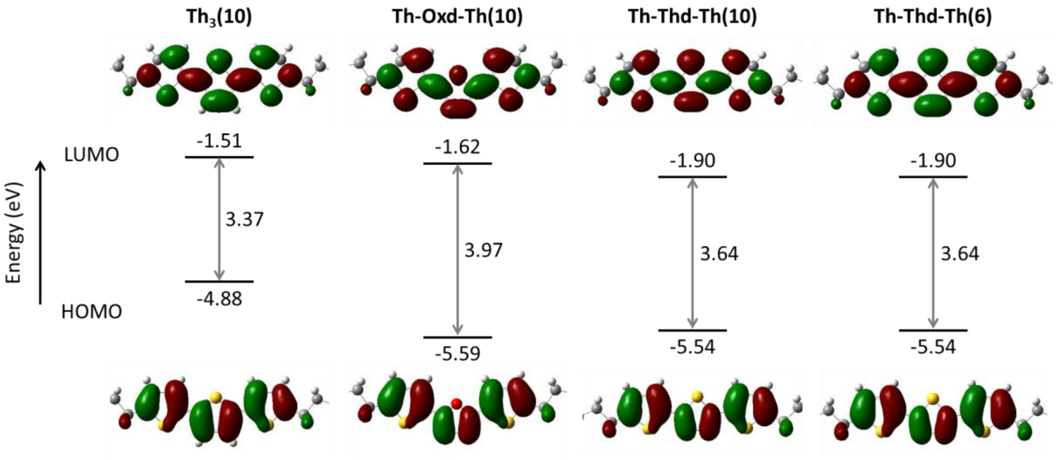

Figure S37. Frontier molecular orbitals of select compounds calculated by DFT at B3LYP/6-31G* level.

a)

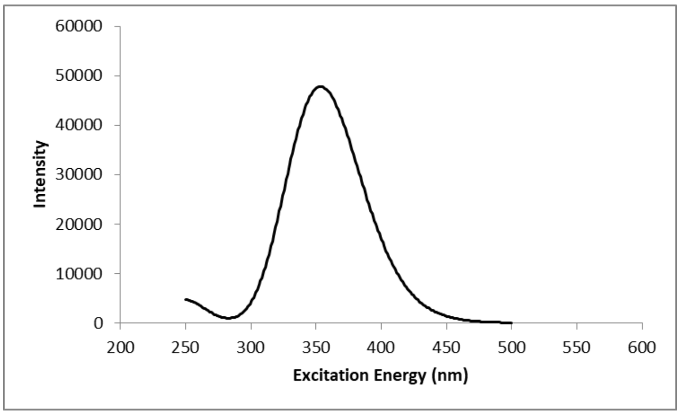

b)

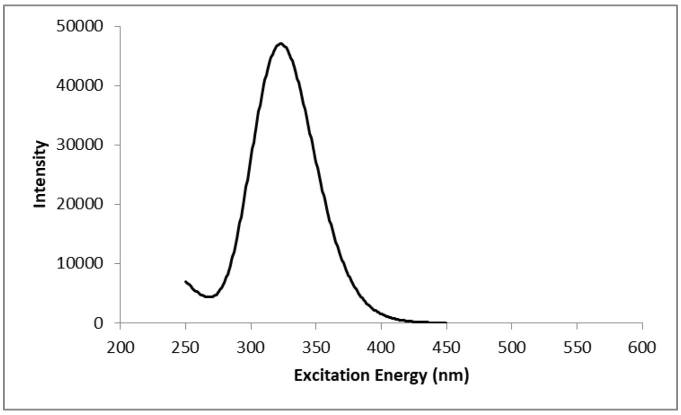

c)

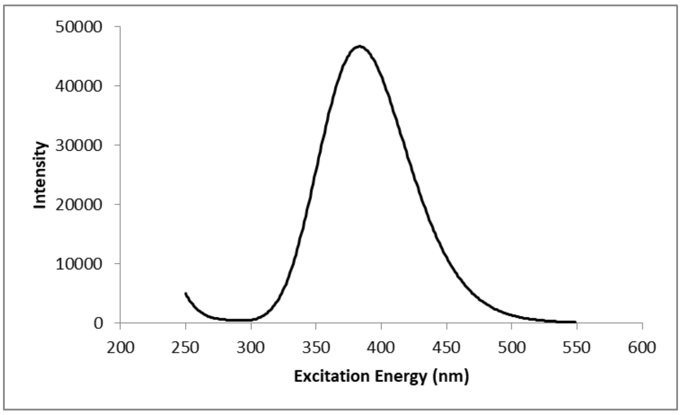

Figure S38. TD-DFT predicted UV/vis spectra in chloroform of **Th$_3$(10)** (a), **Th-Oxd-Th(10)** (b), and **Th-Thd-Th(10)** (c).

11. Complete List of Computational Data

Optimized coordinates (Å) for Th-Oxd-Th(10):

C	1.06861315	-2.08694106	0.00077733
C	-1.06861352	-2.08694115	0.00071126
C	2.39766853	-1.54132287	-0.00139990
C	2.77436144	-0.21785222	-0.00711548
S	3.79545595	-2.59381429	0.00380009
C	4.18572883	-0.04510711	-0.00774990
H	2.06185720	0.59918123	-0.01079215
C	4.88823187	-1.22612957	-0.00253064
H	4.66347744	0.92751470	-0.01198087
C	-2.39766880	-1.54132337	-0.00161983
C	-2.77436145	-0.21785256	-0.00731636
S	-3.79545651	-2.59381625	0.00318546
C	-4.18572881	-0.04510757	-0.00804950
H	-2.06185703	0.59918136	-0.01084878
C	-4.88823212	-1.22613025	-0.00291490
H	-4.66347720	0.92751460	-0.01222002
N	0.69495173	-3.33705542	0.00614971
N	-0.69495230	-3.33705639	0.00589109
C	6.37730125	-1.45485264	-0.00210701
H	6.64913616	-2.06181825	0.87321688

331	H	6.64960845	-2.06146371	-0.87757292
332	C	7.21222653	-0.16644298	-0.00169072
333	H	6.95424034	0.43684545	-0.88254785
334	H	6.95256581	0.43731071	0.87835320
335	C	8.72046191	-0.44679165	-0.00034091
336	H	8.97778842	-1.05622778	-0.87899553
337	H	8.97639213	-1.05525476	0.87939382
338	C	9.57263471	0.82886404	-0.00039303
339	H	9.31489655	1.43723484	-0.87997259
340	H	9.31352133	1.43825006	0.87807735
341	C	11.08204180	0.55434118	0.00093129
342	H	11.34045757	-0.05583227	-0.87730798
343	H	11.33914876	-0.05461481	0.88039787
344	C	11.93592221	1.82897310	0.00067000
345	H	11.67705214	2.43912377	0.87889034
346	H	11.67831709	2.43789854	-0.87877272
347	C	13.44553753	1.55547064	0.00193890
348	H	13.70423043	0.94509834	-0.87622063
349	H	13.70300327	0.94644163	0.88139028
350	C	14.30000861	2.82952366	0.00155407
351	H	14.04226918	3.44048647	0.87974964
352	H	14.04348258	3.43913280	-0.87793698
353	C	15.80965123	2.55653223	0.00280353
354	H	16.06751629	1.94662021	-0.87482891
355	H	16.06631717	1.94801871	0.88175698
356	C	-6.37730151	-1.45485311	-0.00253797
357	H	-6.64960136	-2.06129019	-0.87812652
358	H	-6.64914399	-2.06199258	0.87266316
359	C	-7.21222663	-0.16644339	-0.00187290
360	H	-6.95422436	0.43702490	-0.88260214
361	H	-6.95258163	0.43713048	0.87829899
362	C	-8.72046207	-0.44679200	-0.00060999
363	H	-8.97641050	-1.05543125	0.87899758
364	H	-8.97777064	-1.05605206	-0.87939196
365	C	-9.57263461	0.82886387	-0.00042410
366	H	-9.31487576	1.43741292	-0.87987429
367	H	-9.31354154	1.43807172	0.87817585
368	C	-11.08204175	0.55434112	0.00080914
369	H	-11.33916919	-0.05479501	0.88014500
370	H	-11.34043741	-0.05565229	-0.87756110
371	C	-11.93592184	1.82897329	0.00078963
372	H	-11.67829497	2.43808021	-0.87852102
373	H	-11.67707320	2.43894256	0.87914228
374	C	-13.44553726	1.55547096	0.00196491
375	H	-13.70302454	0.94625918	0.88128339
376	H	-13.70420900	0.94528133	-0.87632779
377	C	-14.30000797	2.82952426	0.00182434
378	H	-14.04345988	3.43931684	-0.87753308
379	H	-14.04229034	3.44030374	0.88015380
380	C	-15.80965070	2.55653298	0.00297909
381	H	-16.06633871	1.94783575	0.88179890
382	H	-16.06749411	1.94680457	-0.87478728

C	-16.65554604	3.83407879	0.00278355
H	-16.44637672	4.44739458	-0.88259487
H	-17.72753839	3.60505452	0.00362778
H	-16.44520612	4.44844191	0.88715775
C	16.65554688	3.83407775	0.00236187
H	17.72753913	3.60505337	0.00328081
H	16.44639984	4.44720832	-0.88315009
H	16.44518506	4.44862597	0.88660225
O	-0.00000010	-1.23211328	-0.00293686

Optimized coordinates (Å) for Th-Thd-Th(10):

C	-1.20806377	0.50845006	-0.08189367
C	1.20806231	0.50844896	-0.08186841
C	-2.62897218	0.26196466	-0.10378363
C	-3.30724199	-0.93120312	-0.21603580
S	-3.75383772	1.60215727	0.02841576
C	-4.72047485	-0.77714338	-0.19652581
H	-2.81147972	-1.89206474	-0.30921571
C	-5.13166814	0.52795398	-0.07024494
H	-5.40815518	-1.61115399	-0.27339070
C	2.62897093	0.26196222	-0.10372872
C	3.30724201	-0.93120506	-0.21597812
S	3.75383480	1.60215297	0.02850384
C	4.72047463	-0.77714681	-0.19643585
H	2.81148083	-1.89206528	-0.30917861
C	5.13166637	0.52794882	-0.07013243
H	5.40815598	-1.61115720	-0.27329419
N	-0.67872948	1.70492656	0.03120256
N	0.67872675	1.70492594	0.03121690
C	-6.52843051	1.08964312	-0.01520250
H	-6.63703425	1.69821823	0.89371850
H	-6.67075511	1.78587795	-0.85425675
C	-7.63742108	0.02837637	-0.04680507
H	-7.54715630	-0.56880960	-0.96422752
H	-7.50229530	-0.66611570	0.79336948
C	-9.03990065	0.64625750	0.02226512
H	-9.17222518	1.34284177	-0.81852838
H	-9.12561737	1.25118318	0.93680907
C	-10.16370184	-0.39780305	-0.00177323
H	-10.07753765	-1.00321688	-0.91622769
H	-10.02923954	-1.09455842	0.83884452
C	-11.56787885	0.21628248	0.06913067
H	-11.70294970	0.91089331	-0.77327290
H	-11.65144896	0.82523051	0.98159146
C	-12.69382056	-0.82574116	0.05176856
H	-12.55817735	-1.52007882	0.89440866
H	-12.61038804	-1.43514402	-0.86048329
C	-14.09796667	-0.21162192	0.12344441
H	-14.23434321	0.48104843	-0.72048688
H	-14.18006524	0.39991695	1.03443403
C	-15.22472807	-1.25259941	0.11041363
H	-15.08953979	-1.94519598	0.95476991

H	-15.14373892	-1.86493410	-0.80029356
C	-16.62884142	-0.63855282	0.18189774
H	-16.76479738	0.05219514	-0.66254269
H	-16.70983608	-0.02632273	1.09146857
C	6.52842797	1.08963542	-0.01504594
H	6.67075337	1.78593260	-0.85404804
H	6.63702916	1.69814343	0.89392059
C	7.63741992	0.02837253	-0.04672512
H	7.54715185	-0.56875040	-0.96418831
H	7.50229921	-0.66617767	0.79340221
C	9.03989870	0.64625129	0.02238311
H	9.12562012	1.25110739	0.93697263
H	9.17221608	1.34290006	-0.81835809
C	10.16370221	-0.39780474	-0.00174333
H	10.07753458	-1.00314607	-0.91624547
H	10.02924615	-1.09462729	0.83881974
C	11.56787801	0.21627877	0.06920174
H	11.65145398	0.82514851	0.98171422
H	11.70293960	0.91096257	-0.77314316
C	12.69382278	-0.82573984	0.05173967
H	12.61038518	-1.43506245	-0.86056533
H	12.55818835	-1.52015233	0.89431953
C	14.09796741	-0.21162234	0.12345875
H	14.18007275	0.39983248	1.03450416
H	14.23433321	0.48112666	-0.72040975
C	15.22473246	-1.25259453	0.11032012
H	15.14373686	-1.86484426	-0.80044361
H	15.08955482	-1.94527067	0.95461285
C	16.62884428	-0.63854945	0.18184824
H	16.70984597	-0.02640562	1.09147650
H	16.76478881	0.05227942	-0.66252784
S	-0.00000002	-0.77125293	-0.20301612
C	17.74810507	-1.68492171	0.17003676
H	17.71426080	-2.29059304	-0.74418072
H	18.73699733	-1.21476990	0.22174887
H	17.65926346	-2.36929533	1.02298639
C	-17.74809840	-1.68493049	0.17019756
H	-18.73699188	-1.21477756	0.22187505
H	-17.71426122	-2.29068893	-0.74396246
H	-17.65924526	-2.36922236	1.02321157

Optimized coordinates (Å) for $Th_3(10)$:

C	1.26837879	-1.21007656	0.00008198
C	0.70852443	-2.47104433	0.00033753
C	-0.70852454	-2.47104417	0.00033264
C	-1.26837862	-1.21007627	0.00007325
S	0.00000022	0.01108963	-0.00024612
H	1.30156813	-3.37954852	0.00055460
H	-1.30156847	-3.37954821	0.00053860
C	2.66280229	-0.82805285	0.00003919
C	3.22398045	0.42972758	-0.00007935
S	3.93527594	-2.04662194	0.00014213

485	C	4.64875931	0.42350348	-0.00008727
486	H	2.63410326	1.34072849	-0.00015208
487	C	5.20090459	-0.83106076	0.00002478
488	H	5.24307275	1.33020577	-0.00017238
489	C	-2.66280200	-0.82805223	0.00002622
490	C	-3.22397990	0.42972830	-0.00029443
491	S	-3.93527604	-2.04662098	0.00043851
492	C	-4.64875882	0.42350458	-0.00026313
493	H	-2.63410247	1.34072902	-0.00056956
494	C	-5.20090450	-0.83105947	0.00007857
495	H	-5.24307197	1.33020702	-0.00051052
496	C	6.64764594	-1.25032333	0.00005105
497	H	6.84287393	-1.88653100	0.87558288
498	H	6.84287901	-1.88662508	-0.87541167
499	C	-6.64764594	-1.25032175	0.00018201
500	H	-6.84289086	-1.88674621	-0.87518910
501	H	-6.84286218	-1.88640668	0.87580539
502	C	7.63992573	-0.07865094	-0.00001001
503	H	7.45912084	0.55298895	-0.88027122
504	H	7.45910740	0.55309150	0.88017475
505	C	-7.63992573	-0.07864935	-0.00002881
506	H	-7.45909269	0.55321695	0.88006408
507	H	-7.45913566	0.55286667	-0.88038190
508	C	-9.10082769	-0.54674119	0.00009762
509	H	-9.27876687	-1.18346403	-0.87896767
510	H	-9.27872242	-1.18312624	0.87941647
511	C	9.10082774	-0.54674268	0.00002772
512	H	9.27873888	-1.18324276	0.87925998
513	H	9.27875073	-1.18335047	-0.87912420
514	C	10.10788798	0.61059592	-0.00003658
515	H	9.92872375	1.24731056	-0.87907160
516	H	9.92870528	1.24742444	0.87891218
517	C	-10.10788803	0.61059728	-0.00009860
518	H	-9.92873544	1.24720246	-0.87921525
519	H	-9.92869396	1.24753527	0.87876850
520	C	-11.57042912	0.14715030	0.00002368
521	H	-11.74885435	-0.49003806	0.87900680
522	H	-11.74889954	-0.49036015	-0.87871669
523	C	11.57042906	0.14714916	0.00000916
524	H	11.74886742	-0.49014292	0.87891440
525	H	11.74888663	-0.49025751	-0.87880911
526	C	12.57923310	1.30306212	-0.00005519
527	H	12.40049099	1.94038510	-0.87892945
528	H	12.40046707	1.94050387	0.87872807
529	C	-12.57923331	1.30306307	-0.00016181
530	H	-12.40050068	1.94028629	-0.87911035
531	H	-12.40045802	1.94060463	0.87854715
532	C	-14.04195172	0.84012335	-0.00004244
533	H	-14.22049793	0.20274951	0.87889690
534	H	-14.22054301	0.20243838	-0.87874691
535	C	-15.05135891	1.99530071	-0.00022078
536	H	-14.87386773	2.63313839	-0.87918136

H	-14.87382402	2.63344742	0.87850664
C	-16.51415231	1.53281315	-0.00010308
H	-16.69196820	0.89604880	0.87832949
H	-16.69201296	0.89574338	-0.87830512
C	14.04195156	0.84012266	-0.00000369
H	14.22053285	0.20253421	-0.87878026
H	14.22050803	0.20265236	0.87886359
C	15.05135854	1.99530022	-0.00006701
H	14.87385921	2.63323209	-0.87895759
H	14.87383159	2.63335269	0.87873044
C	16.51415205	1.53281294	-0.00001206
H	16.69200450	0.89583600	-0.87828311
H	16.69197640	0.89595580	0.87835153
C	17.51529582	2.69276663	-0.00007501
H	17.38491046	3.32826236	0.88475111
H	18.54962514	2.32973201	-0.00003320
H	17.38493935	3.32814112	-0.88499243
C	-17.51529629	2.69276667	-0.00027913
H	-17.38494750	3.32804847	-0.88526422
H	-18.54962551	2.32973186	-0.00019049
H	-17.38490347	3.32835509	0.88447929

TD-DFT excitation energies and oscillator strengths for Th-Oxd-Th(10):

Excited State 1: Singlet-A 3.8397 eV 322.90 nm f = 1.1612 <S**2> = 0.000
139 ->142 0.16639
140 ->141 0.67844
This state for optimization and/or second-order correction.
Total Energy, E(TD-HF/TD-DFT) = -2151.57241331
Copying the excited state density for this state as the 1-particle RhoCI density.
Excited State 2: Singlet-A 4.7066 eV 263.42 nm f = 0.0585 <S**2> = 0.000
138 ->141 0.10602
139 ->141 0.39208
140 ->142 0.56073
Excited State 3: Singlet-A 5.1694 eV 239.84 nm f = 0.0007 <S**2> = 0.000
137 ->141 0.58692
138 ->142 -0.35150
Excited State 4: Singlet-A 5.1769 eV 239.50 nm f = 0.1757 <S**2> = 0.000
137 ->142 -0.34830
138 ->141 0.57678
139 ->141 -0.12485
Excited State 5: Singlet-A 5.3774 eV 230.57 nm f = 0.0001 <S**2>=0.000
133 ->142 0.12674
139 ->141 0.55159
140 ->142 -0.40040
Excited State 6: Singlet-A 5.6984 eV 217.58 nm f=0.0000 <S**2> = 0.000
134 ->141 0.64152
134 ->145 -0.14015
136 ->141 0.20251
Excited State 7: Singlet-A 5.9211 eV 209.39 nm f = 0.0347 <S**2> = 0.000
133 ->141 0.21742
139 ->142 0.61505
140 ->141 -0.13869

140 ->145 0.17856
Excited State 8: Singlet-A 5.9270 eV 209.19 nm f = 0.0000 <S**2> = 0.000
139 ->144 0.33420
140 ->143 0.59840
Excited State 9: Singlet-A 5.9946 eV 206.83 nm f = 0.0008 <S**2> = 0.000
139 ->143 0.37585
140 ->144 0.56656
Excited State 10: Singlet-A 6.2904 eV 197.10 nm f = 0.0067 <S**2>=0.000
116 ->141 -0.10331
118 ->141 -0.21384
122 ->141 -0.17124
134 ->142 0.54317
134 ->150 0.16147
134 ->167 -0.12540
136 ->142 0.17894
Excited State 11: Singlet-A 6.3596 eV 194.96 nm f = 0.0000 <S**2> = 0.000
137 ->143 0.48569
138 ->144 -0.45662
Excited State 12: Singlet-A 6.3618 eV 194.89 nm f = 0.0000 <S**2> = 0.000
134 ->142 -0.10948
137 ->144 -0.45141
138 ->143 0.47057
Excited State 13: Singlet-A 6.5740 eV 188.60 nm = 0.0144 <S**2> = 0.000
133 ->141 -0.16914
137 ->141 -0.30996
137 ->145 0.15244
138 ->142 -0.34737
139 ->150 -0.18597
140 ->145 0.42003
Excited State 14: Singlet-A 6.6269 eV 187.09 nm f=0.0002 <S**2>=0.000
137 ->142 0.50133
138 ->141 0.37664
138 ->145 -0.15985
139 ->145 0.15232
140 ->150 -0.19883
Excited State 15: Singlet-A 6.7394 eV 183.97 nm f=0.0251 <S**2>=0.000
137 ->141 0.20498
137 ->145 -0.10627
138 ->142 0.43762
139 ->142 -0.13396
139 ->150 -0.14540
140 ->145 0.44440
Excited State 16: Singlet-A 6.8924 eV 179.88 nm f=0.0019 <S**2>=0.000
115 ->142 0.11594
116 ->141 0.18119
118 ->141 0.40486
122 ->141 0.36915
134 ->142 0.28832
135 ->141 -0.10094
Excited State 17: Singlet-A 6.9960 eV 177.22 nm f = 0.3755 S**2>=0.000
113 ->141 -0.14081
137 ->142 0.28490

139 ->145 -0.28752
140 ->150 0.52557
Excited State 18: Singlet-A 7.0016 eV 177.08 nm f=0.0091 <S**2>=0.000
133 ->141 0.60542
138 ->142 -0.11100
139 ->142 -0.23165
140 ->141 0.10649
Excited State 19: Singlet-A 7.0156 eV 176.73 nm f=0.0000 <S**2>=0.000
110 ->142 0.10494
112 ->141 -0.10370
114 ->142 0.20899
115 ->141 0.36301
117 ->141 0.10405
121 ->141 0.31625
122 ->142 0.28720
135 ->142 -0.14145
136 ->141 -0.21074
Excited State 20: Singlet-A 7.0690 eV 175.39 nm f=0.0085 <S**2>=0.000
110 ->141 0.17679
114 ->141 0.37381
115 ->142 0.24180
116 ->141 -0.19952
118 ->141 -0.19438
121 ->142 0.21296
122 ->141 0.18842
122 ->145 0.10014
135 ->141 -0.21212
136 ->142 -0.14511
Excited State 21: Singlet-A 7.0864 eV 174.96 nm f=0.0000 <S**2>=0.000
139 ->144 -0.16326
140 ->147 -0.13963
140 ->149 0.13149
140 ->152 -0.21062
140 ->153 0.29669
140 ->154 0.32012
140 ->159 -0.27770
140 ->162 -0.23557
Excited State 22: Singlet-A 7.3232 eV 169.30 nm f=0.0000 <S**2>=0.000
139 ->147 -0.18742
139 ->149 0.16438
139 ->152 -0.14061
140 ->144 -0.12821
140 ->146 0.34304
140 ->148 -0.28060
140 ->151 0.21270
140 ->161 -0.16377
140 ->163 0.13354
140 ->169 -0.11490
140 ->170 0.15360
Excited State 23: Singlet-A 7.4056 eV 167.42 nm f=0.0000 <S**2>=0.000
139 ->144 0.14018
139 ->158 0.11313

139 ->163 -0.19155
140 ->153 0.50296
140 ->159 0.18105
140 ->162 0.22337
140 ->168 0.12670
Excited State 24: Singlet-A 7.4385 eV 166.68 nm f=0.0000 <S**2>=0.000
136 ->141 0.11977
139 ->146 -0.19282
139 ->148 0.16348
139 ->151 -0.13568
140 ->147 0.33618
140 ->149 -0.26475
140 ->152 0.16583
140 ->153 0.11341
140 ->154 0.10884
140 ->159 -0.11322
140 ->160 -0.15120
140 ->162 -0.14973
140 ->168 -0.11390
Excited State 25: Singlet-A 7.4553 eV 166.30 nm f=0.0009 <S**2>=0.000
139 ->143 0.16129
139 ->153 -0.11429
139 ->154 0.10306
139 ->159 -0.15927
139 ->162 -0.17987
139 ->168 -0.11816
140 ->146 -0.15137
140 ->148 0.12997
140 ->151 -0.13059
140 ->155 0.15112
140 ->158 -0.26395
140 ->163 0.37400
140 ->170 0.15430
Excited State 26: Singlet-A 7.5037 eV 165.23 nm f=0.0000 <S**2>=0.000
104 ->142 -0.13497
105 ->141 0.13491
107 ->141 0.17020
110 ->142 0.14865
112 ->141 -0.20770
114 ->142 0.13178
115 ->141 0.29385
121 ->141 -0.12739
134 ->141 -0.11645
135 ->142 0.15633
136 ->141 0.33751
140 ->147 -0.12163
140 ->153 -0.11847
Excited State 27: Singlet-A 7.5451 eV 164.32 nm f=0.0224 <S**2>=0.000
108 ->141 -0.13687
111 ->141 0.18370
113 ->141 0.16512
133 ->142 0.44961

137 ->150 0.21846
138 ->145 0.23926
139 ->141 -0.10379
140 ->167 0.18617
Excited State 28: Singlet-A 7.5572 eV 164.06 nm f=0.0000 <S**2>=0.000
114 ->142 -0.21459
116 ->142 0.25632
118 ->142 0.45963
118 ->150 0.13839
118 ->167 -0.10476
122 ->142 0.25208
Excited State 29: Singlet-A 7.5636 eV 163.92 nm f=0.0011 <S**2>=0.000
104 ->141 -0.20068
105 ->142 0.10349
107 ->142 0.12348
110 ->141 0.24180
112 ->142 -0.15071
114 ->141 0.23230
115 ->142 0.21505
118 ->141 0.11659
135 ->141 0.37713
136 ->142 0.17981
Excited State 30: Singlet-A 7.6332 eV 162.43 nm f=0.2315 <S**2>=0.000
137 ->145 0.46672
138 ->142 0.18205
138 ->150 0.40275
139 ->150 -0.12803
140 ->166 -0.13622

TD-DFT excitation energies and oscillator strengths for Th-Thd-Th(10):

Excited State 1: Singlet-A 3.5070 eV 353.53 nm f=1.1804 <S**2>=0.000
143 ->146 0.13462
144 ->145 0.68560
This state for optimization and/or second-order correction.
Total Energy, E(TD-HF/TD-DFT) = -2474.55713381
Copying the excited state density for this state as the 1-particle RhoCI density.
Excited State 2: Singlet-A 4.4743 eV 277.10 nm f=0.0001 <S**2>=0.000
143 ->145 0.54962
144 ->146 0.41215
Excited State 3: Singlet-A 4.7187 eV 262.75 nm f=0.0000 <S**2>=0.000
140 ->145 0.68229
140 ->148 0.13663
Excited State 4: Singlet-A 4.9413 eV 250.92 nm f=0.0964 <S**2>=0.000
141 ->146 -0.27999
142 ->145 0.60751
143 ->145 0.16448
Excited State 5: Singlet-A 4.9658 eV 249.67 nm f=0.0061 <S**2>=0.000
141 ->145 0.62700
142 ->146 -0.28821
Excited State 6: Singlet-A 5.1295 eV 241.71 nm f=0.0139 <S**2>=0.000
142 ->145 0.14946
143 ->145 -0.38413

143 ->148 -0.12713
144 ->146 0.53809
Excited State 7: Singlet-A 5.3658 eV 231.06 nm f=0.0006 <S**2>=0.000
136 ->147 -0.13272
144 ->147 0.67553
Excited State 8: Singlet-A 5.6407 eV 219.80 nm f=0.0013 <S**2>=0.000
133 ->145 0.52447
140 ->146 -0.40254
140 ->155 -0.12979
144 ->147 -0.10696
Excited State 9: Singlet-A 5.7387 eV 216.05 nm f=0.1112 <S**2>=0.000
136 ->145 0.21706
143 ->146 0.58147
144 ->148 -0.28253
Excited State 10: Singlet-A 5.8126 eV 213.30 nm f=0.0303 <S**2>=0.000
136 ->146 -0.10062
137 ->145 0.64223
140 ->147 0.16350
Excited State 11: Singlet-A 5.8558 eV 211.73 nm f=0.0002 <S**2>=0.000
143 ->147 -0.18070
143 ->150 0.26175
144 ->149 0.59726
Excited State 12: Singlet-A 6.0214 eV 205.91 nm f=0.0011 <S**2>=0.000
133 ->145 -0.14580
140 ->146 -0.11898
143 ->149 0.36392
144 ->150 0.50896
144 ->152 -0.15647
Excited State 13: Singlet-A 6.1099 eV 202.92 nm f=0.0038 <S**2>=0.000
133 ->145 0.39510
140 ->146 0.49361
140 ->155 0.14768
143 ->149 0.10918
144 ->150 0.14781
Excited State 14: Singlet-A 6.1768 eV 200.73 nm f=0.0000 <S**2>=0.000
137 ->147 0.38238
142 ->147 0.10202
143 ->147 0.53709
143 ->150 0.10905
144 ->149 0.13514
Excited State 15: Singlet-A 6.3335 eV 195.76 nm f=0.0067 <S**2>=0.000
136 ->145 0.30318
141 ->145 -0.20038
141 ->148 -0.16839
142 ->146 -0.28521
143 ->155 0.18149
144 ->148 0.43790
Excited State 16: Singlet-A 6.3823 eV 194.26 nm f=0.0002 <S**2>=0.000
141 ->147 0.15331
141 ->150 -0.40636
141 ->152 0.12986
142 ->149 0.47299

Excited State	17:	Singlet-A	6.3871 eV	194.12 nm	f=0.0000	<S**2>=0.000
141 ->149		0.47975				
142 ->147		0.13512				
142 ->150		-0.39778				
142 ->152		0.12746				
143 ->150		-0.10594				
Excited State	18:	Singlet-A	6.4570 eV	192.01 nm	f=0.0031	<S**2>=0.000
137 ->145		-0.10085				
141 ->146		0.50008				
142 ->145		0.30150				
142 ->148		0.21920				
143 ->148		0.17744				
144 ->155		0.19138				
Excited State	19:	Singlet-A	6.5242 eV	190.04 nm	f=0.0757	<S**2>=0.000
141 ->145		0.20347				
141 ->148		0.17401				
142 ->146		0.44590				
143 ->146		0.20349				
144 ->148		0.37967				
Excited State	20:	Singlet-A	6.6944 eV	185.21 nm	f=0.0010	<S**2>=0.000
136 ->145		0.55620				
137 ->146		-0.11522				
141 ->145		0.10055				
142 ->146		0.20532				
143 ->146		-0.24397				
144 ->145		0.11098				
144 ->148		-0.10053				
Excited State	21:	Singlet-A	6.8314 eV	181.49 nm	f=0.2001	<S**2>=0.000
140 ->147		-0.27549				
141 ->146		-0.26409				
143 ->148		0.29197				
144 ->153		-0.12434				
144 ->155		0.43394				
Excited State	22:	Singlet-A	6.8374 eV	181.33 nm	f=0.0000	<S**2>=0.000
117 ->146		-0.15989				
118 ->145		0.37991				
123 ->146		0.13395				
124 ->145		0.27591				
133 ->146		0.36296				
139 ->145		-0.17930				
Excited State	23:	Singlet-A	6.9028 eV	179.61 nm	f=0.0064	<S**2>=0.000
114 ->145		-0.14222				
117 ->145		-0.33787				
118 ->146		0.23649				
119 ->145		0.12307				
123 ->145		0.34030				
124 ->146		0.21139				
138 ->145		-0.26525				
139 ->146		-0.12211				
Excited State	24:	Singlet-A	6.9601 eV	178.14 nm	f=0.0000	<S**2>=0.000
137 ->147		-0.12830				
143 ->150		0.22079				

143 ->178		-0.11140				
144 ->156		0.14121				
144 ->158		0.47414				
144 ->163		-0.18195				
144 ->169		0.20726				
Excited State	25:	Singlet-A	7.0702 eV	175.36 nm	f=0.0000	<S**2>=0.000
111 ->145		-0.13921				
117 ->146		0.10414				
123 ->146		-0.17663				
124 ->145		-0.21380				
133 ->146		0.44611				
133 ->155		0.15483				
139 ->145		0.27099				
144 ->158		-0.12091				
Excited State	26:	Singlet-A	7.1966 eV	172.28 nm	f=0.0000	<S**2>=0.000
118 ->145		-0.12226				
137 ->147		-0.48892				
139 ->145		-0.11810				
143 ->147		0.32931				
144 ->158		-0.19148				
144 ->159		-0.10344				
Excited State	27:	Singlet-A	7.2334 eV	171.41 nm	f=0.0000	<S**2>=0.000
110 ->146		-0.10281				
111 ->145		-0.20097				
114 ->146		-0.13037				
115 ->145		-0.16591				
117 ->146		-0.17092				
118 ->145		0.35564				
133 ->146		-0.26648				
133 ->155		-0.10352				
137 ->147		-0.15400				
139 ->145		0.26259				
143 ->147		0.11741				
Excited State	28:	Singlet-A	7.2689 eV	170.57 nm	f=0.0132	<S**2>=0.000
133 ->147		-0.12287				
137 ->146		0.62523				
137 ->155		0.12744				
143 ->146		-0.11783				
144 ->148		-0.10865				
Excited State	29:	Singlet-A	7.2892 eV	170.09 nm	f=0.0107	<S**2>=0.000
140 ->147		-0.12446				
143 ->151		0.18465				
143 ->154		-0.16801				
143 ->156		0.14777				
143 ->165		0.10297				
144 ->150		0.20034				
144 ->152		0.30387				
144 ->153		0.28802				
144 ->157		-0.21871				
144 ->166		0.15341				
Excited State	30:	Singlet-A	7.3187 eV	169.41 nm	f=0.0002	<S**2>=0.000
143 ->152		0.18274				

```
143 ->153      0.16843
143 ->157     -0.14315
144 ->151      0.34681
144 ->154     -0.29297
144 ->156      0.21631
144 ->163      0.14280
144 ->165      0.15538
144 ->169     -0.18547
```

TD-DFT excitation energies and oscillator strengths for Th_3(10):

```
Excited State   1:   Singlet-A   3.2352 eV  383.23 nm  f=1.1501  <S**2>=0.000
143 ->146      0.10496
144 ->145      0.69331
This state for optimization and/or second-order correction.
Total Energy, E(TD-HF/TD-DFT) =  -2442.48397494
Copying the excited state density for this state as the 1-particle RhoCI density.
Excited State   2:   Singlet-A   4.4727 eV  277.20 nm  f=0.0114  <S**2>=0.000
140 ->145     -0.13771
143 ->145      0.41305
144 ->146      0.53540
Excited State   3:   Singlet-A   4.9600 eV  249.97 nm  f=0.0009  <S**2>=0.000
139 ->146     -0.12084
140 ->145      0.12421
142 ->145      0.24007
143 ->145      0.52412
144 ->146     -0.33903
Excited State   4:   Singlet-A   5.0025 eV  247.84 nm  f=0.0001  <S**2>=0.000
140 ->145      0.18829
141 ->146     -0.15922
142 ->145      0.57887
143 ->145     -0.17322
144 ->146      0.24500
Excited State   5:   Singlet-A   5.1535 eV  240.58 nm  f=0.0025  <S**2>=0.000
140 ->146      0.11974
141 ->145      0.61946
142 ->146     -0.24148
144 ->149      0.12542
Excited State   6:   Singlet-A   5.2003 eV  238.42 nm  f=0.0002  <S**2>=0.000
139 ->148      0.11993
144 ->148      0.65392
144 ->150      0.20644
Excited State   7:   Singlet-A   5.2659 eV  235.45 nm  f=0.2023  <S**2>=0.000
140 ->145      0.59885
141 ->146      0.21978
142 ->145     -0.17621
143 ->149      0.10311
144 ->146      0.11921
144 ->157     -0.12382
Excited State   8:   Singlet-A   5.3190 eV  233.10 nm  f=0.0000  <S**2>=0.000
143 ->150     -0.22838
144 ->147      0.63700
Excited State   9:   Singlet-A   5.4861 eV  226.00 nm  f=0.0009  <S**2>=0.000
```

143 ->147		-0.30436				
144 ->148		-0.19118				
144 ->150		0.56937				
Excited State	10:	Singlet-A	5.7371 eV	216.11 nm	f=0.0898	<S**2>=0.000
139 ->145		-0.19445				
141 ->145		-0.14198				
143 ->146		0.38087				
144 ->149		0.51601				
Excited State	11:	Singlet-A	6.0924 eV	203.51 nm	f=0.0080	<S**2>=0.000
139 ->145		0.51640				
140 ->146		0.11207				
143 ->146		-0.24081				
143 ->157		-0.10360				
144 ->149		0.35163				
Excited State	12:	Singlet-A	6.2170 eV	199.43 nm	f=0.0000	<S**2>=0.000
140 ->148		0.43473				
140 ->150		0.17204				
142 ->148		0.35622				
143 ->148		-0.32545				
Excited State	13:	Singlet-A	6.2258 eV	199.15 nm	f=0.0001	<S**2>=0.000
140 ->147		-0.21337				
141 ->148		-0.15016				
141 ->150		0.42272				
142 ->147		0.41946				
143 ->147		-0.14830				
Excited State	14:	Singlet-A	6.2301 eV	199.01 nm	f=0.0000	<S**2>=0.000
140 ->148		0.16562				
140 ->150		-0.16123				
141 ->147		0.47248				
142 ->150		0.38420				
143 ->150		-0.12741				
Excited State	15:	Singlet-A	6.3848 eV	194.19 nm	f=0.0000	<S**2>=0.000
139 ->147		0.10077				
143 ->150		0.20691				
144 ->158		0.12446				
144 ->160		-0.29750				
144 ->162		0.52143				
144 ->166		-0.10013				
144 ->173		-0.10046				
Excited State	16:	Singlet-A	6.5583 eV	189.05 nm	f=0.0785	<S**2>=0.000
139 ->145		-0.21790				
140 ->146		0.22034				
141 ->145		0.17733				
141 ->149		-0.15636				
142 ->146		0.47097				
143 ->146		-0.28815				
144 ->149		0.13947				
Excited State	17:	Singlet-A	6.5981 eV	187.91 nm	f=0.0572	<S**2>=0.000
140 ->145		0.22905				
141 ->146		-0.35047				
142 ->145		-0.20032				
142 ->149		0.18471				

143 ->149		-0.25489				
144 ->157		0.35732				
144 ->163		0.12281				
Excited State	18:	Singlet-A	6.7410 eV	183.93 nm	f=0.0047	<S**2>=0.000
121 ->146		-0.15110				
124 ->145		-0.43713				
126 ->145		0.48565				
Excited State	19:	Singlet-A	6.7490 eV	183.71 nm	f=0.0404	<S**2>=0.000
139 ->145		0.32421				
140 ->146		0.17189				
142 ->146		0.32710				
143 ->146		0.41507				
144 ->145		-0.11702				
144 ->149		-0.18198				
Excited State	20:	Singlet-A	6.7642 eV	183.29 nm	f=0.0000	<S**2>=0.000
140 ->148		0.10227				
143 ->148		0.12138				
143 ->168		-0.10086				
144 ->151		0.14359				
144 ->153		-0.14284				
144 ->156		0.16845				
144 ->160		0.50528				
144 ->162		0.26350				
Excited State	21:	Singlet-A	6.8325 eV	181.46 nm	f=0.0029	<S**2>=0.000
139 ->145		-0.12983				
140 ->146		0.55172				
141 ->145		-0.19479				
141 ->149		0.24496				
142 ->146		-0.10988				
144 ->169		0.10752				
144 ->172		0.12705				
Excited State	22:	Singlet-A	6.8371 eV	181.34 nm	f=0.0005	<S**2>=0.000
143 ->151		0.15153				
143 ->153		-0.14615				
143 ->156		0.12087				
144 ->150		-0.12148				
144 ->152		0.39264				
144 ->154		-0.35542				
144 ->155		0.26005				
144 ->164		0.14586				
Excited State	23:	Singlet-A	6.8658 eV	180.58 nm	f=0.0000	<S**2>=0.000
143 ->152		0.13304				
143 ->154		-0.13296				
143 ->155		0.10352				
144 ->151		0.37744				
144 ->153		-0.31825				
144 ->156		0.19876				
144 ->160		-0.23391				
144 ->166		0.17936				
144 ->175		0.12662				
Excited State	24:	Singlet-A	6.9527 eV	178.33 nm	f=0.5434	<S**2>=0.000
140 ->149		0.13155				

141 ->146		0.40916				
142 ->145		0.13324				
142 ->149		-0.18033				
143 ->149		-0.18570				
144 ->157		0.40079				
144 ->163		0.11667				
144 ->170		-0.13318				
144 ->171		0.11015				
Excited State	25:	Singlet-A	6.9796 eV	177.64 nm	f=0.0000	<S**2>=0.000
121 ->145		0.51090				
125 ->145		0.32714				
126 ->146		-0.25877				
Excited State	26:	Singlet-A	7.0175 eV	176.68 nm	f=0.0009	<S**2>=0.000
139 ->148		0.10075				
143 ->147		-0.17571				
143 ->158		-0.12106				
143 ->162		-0.22564				
144 ->150		-0.13309				
144 ->159		-0.10324				
144 ->164		-0.28123				
144 ->168		0.44776				
Excited State	27:	Singlet-A	7.1144 eV	174.27 nm	f=0.0000	<S**2>=0.000
140 ->148		0.24262				
142 ->148		0.10358				
143 ->148		0.45693				
143 ->150		0.21545				
144 ->162		-0.23352				
144 ->165		0.16471				
144 ->173		-0.14445				
Excited State	28:	Singlet-A	7.1596 eV	173.17 nm	f=0.0020	<S**2>=0.000
114 ->145		-0.17717				
118 ->145		-0.28277				
119 ->145		0.10392				
121 ->146		0.11144				
124 ->145		-0.26405				
125 ->146		0.23664				
126 ->145		-0.26854				
126 ->149		-0.11796				
137 ->145		0.28692				
138 ->146		-0.12358				
Excited State	29:	Singlet-A	7.2077 eV	172.02 nm	f=0.0083	<S**2>=0.000
139 ->146		-0.13113				
141 ->146		0.12497				
143 ->169		-0.11253				
143 ->172		-0.13142				
144 ->157		0.16827				
144 ->170		0.57395				
144 ->171		-0.19441				
Excited State	30:	Singlet-A	7.2624 eV	170.72 nm	f=0.0000	<S**2>=0.000
112 ->145		0.25008				
116 ->145		0.13405				
120 ->145		-0.10514				

121 ->145	0.23442
124 ->146	0.14355
125 ->145	-0.25429
137 ->146	-0.13388
138 ->145	0.35980
143 ->148	-0.12958

References

1. Lavigueur, C.; Foster, E.J.; Williams, V.E. A simple and inexpensive capillary furnace for variable-temperature X-ray diffraction. *J. Appl. Crystallogr.* **2008**, *41*, 214–216.
2. Boucher, N.; Leroy, J.; Sergeyev, S.; Pouzet, E.; Lemaur, V.; Lazzaroni, R.; Cornil, J.; Geerts, Y.H.; Sferrazza, M. Mesomorphism of dialkylterthiophene homologues. *Synth. Met.* **2009**, *159*, 1319–1324.
3. Bruker, APEX3, SAINT and SADABS, Bruker AXS Inc., Madison, Wisconsin, USA, 2016.
4. G. M. Sheldrick, TWINABS (Version 2012/1), University of Gçttingen, Germany, 1996.
5. Sheldrick, G.M. Crystal structure refinement with SHELXL. *Acta Crystallogr. Sect. C Struct. Chem.* **2015**, *71*, 3–8.
6. Hübschle, C.B.; Sheldrick, G.M.; Dittrich, B. ShelXle: a Qt graphical user interface for SHELXL. *J. Appl. Crystallogr.* **2011**, *44*, 1281–1284.
7. Farrugia, L.J. WinGX and ORTEP for Windows: an update. *J. Appl. Crystallogr.* **2012**, *45*, 849–854.
8. Fenn, T.D.; Ringe, D.; Petsko, G.A. POVScript+: a program for model and data visualization using persistence of vision ray-tracing. *J. Appl. Crystallogr.* **2003**, *36*, 944–947.
9. Lercher, C.; Röthel, C.; Roscioni, O.M.; Geerts, Y.H.; Shen, Q.; Teichert, C.; Fischer, R.; Leising, G.; Sferrazza, M.; Gbabode, G.; et al. Polymorphism of dioctyl-terthiophene within thin films: The role of the first monolayer. *Chem. Phys. Lett.* **2015**, *630*, 12–17.
10. Van Bolhuis, F.; Wynberg, H.; Havinga, E.E.; Meijer, E.W.; Staring, E.G.J. The X-ray structure and MNDO calculations of α-terthienyl: A model for polythiophenes. *Synth. Met.* **1989**, *30*, 381–389.
11. Resch-Genger, U.; Rurack, K. Determination of the photoluminescence quantum yield of dilute dye solutions (IUPAC Technical Report). *Pure Appl. Chem.* **2013**, *85*, 2005–2013.
12. Brouwer, A.M. Standards for photoluminescence quantum yield measurements in solution (IUPAC Technical Report). *Pure Appl. Chem.* **2011**, *83*, 2213–2228.
13. Stephens, P.J.; Devlin, F.J.; Chabalowski, C.F.; Frisch, M.J. Ab Initio Calculation of Vibrational Absorption and Circular Dichroism Spectra Using Density Functional Force Fields. *J. Phys. Chem.* **1994**, *98*, 11623–11627.
14. Becke, A.D. Density-functional thermochemistry. III. The role of exact exchange. *J. Chem. Phys.* **1993**, *98*, 5648–5652.
15. Tomasi, J.; Mennucci, B.; Cancès, E. The IEF version of the PCM solvation method: an overview of a new method addressed to study molecular solutes at the QM ab initio level. *J. Mol. Struct. THEOCHEM* **1999**, *464*, 211–226.
16. Barone, V.; Cossi, M.; Tomasi, J. Geometry optimization of molecular structures in solution by the polarizable continuum model. *J. Comput. Chem.* **1998**, *19*, 404–417.
17. Barone, V.; Cossi, M.; Tomasi, J. A new definition of cavities for the computation of solvation free energies by the polarizable continuum model. *J. Chem. Phys.* **1997**, *107*, 3210–3221.
18. Miertuš, S.; Scrocco, E.; Tomasi, J. Electrostatic interaction of a solute with a continuum. A direct utilizaion of AB initio molecular potentials for the prevision of solvent effects. *Chem. Phys.* **1981**, *55*, 117–129.
19. Gaussian 16, Revision B.01, M. J. Frisch, G. W. Trucks, H. B. Schlegel, G. E. Scuseria, M. A. Robb, J. R. Cheeseman, G. Scalmani, V. Barone, G. A. Petersson, H. Nakatsuji, X. Li, M. Caricato, A. V. Marenich, J. Bloino, B. G. Janesko, R. Gomperts, B. Mennucci, H. P. Hratchian, J. V. Ortiz, A. F. Izmaylov, J. L. Sonnenberg, D. Williams-Young, F. Ding, F. Lipparini, F. Egidi, J. Goings, B. Peng, A. Petrone, T. Henderson, D. Ranasinghe, V. G. Zakrzewski, J. Gao, N. Rega, G. Zheng, W. Liang, M. Hada, M. Ehara, K. Toyota, R. Fukuda, J. Hasegawa, M. Ishida, T. Nakajima, Y. Honda, O. Kitao, H. Nakai, T. Vreven, K. Throssell, J. A. Montgomery, Jr., J. E. Peralta, F. Ogliaro, M. J. Bearpark, J. J. Heyd, E. N. Brothers, K. N. Kudin, V. N. Staroverov, T. A. Keith, R. Kobayashi, J. Normand, K. Raghavachari, A. P. Rendell, J. C. Burant, S. S. Iyengar, J. Tomasi, M. Cossi, J. M. Millam, M. Klene, C. Adamo, R. Cammi, J. W. Ochterski, R. L. Martin, K. Morokuma, O. Farkas, J. B. Foresman, and D. J. Fox, Gaussian, Inc., Wallingford CT, 2016.

20. Schäfer, A.; Huber, C.; Ahlrichs, R. Fully optimized contracted Gaussian basis sets of triple zeta valence quality for atoms Li to Kr. *J. Chem. Phys.* **1994**, *100*, 5829–5835.
21. Schäfer, A.; Horn, H.; Ahlrichs, R. Fully optimized contracted Gaussian basis sets for atoms Li to Kr. *J. Chem. Phys.* **1992**, *97*, 2571–2577.
22. Stratmann, R.E.; Scuseria, G.E.; Frisch, M.J. An efficient implementation of time-dependent density-functional theory for the calculation of excitation energies of large molecules. *J. Chem. Phys.* **1998**, *109*, 8218–8224.
23. Casida, M.E. Time-Dependent Density Functional Response Theory for Molecules. In *Recent Advances in Density Functional Methods*; Recent Advances in Computational Chemistry; WORLD SCIENTIFIC, 1995; Vol. Volume 1, pp. 155–192 ISBN 978-981-02-2442-4.
24. Huff, G.S.; Gallaher, J.K.; Hodgkiss, J.M.; Gordon, K.C. No single DFT method can predict Raman cross-sections, frequencies and electronic absorption maxima of oligothiophenes. *Synth. Met.* **2017**, *231*, 1–6.
25. Jacquemin, D.; Michaux, C.; Perpète, E.A.; Maurel, F.; Perrier, A. Photochromic molecular wires: Insights from theory. *Chem. Phys. Lett.* **2010**, *488*, 193–197.
26. Yanai, T.; Tew, D.P.; Handy, N.C. A new hybrid exchange–correlation functional using the Coulomb-attenuating method (CAM-B3LYP). *Chem. Phys. Lett.* **2004**, *393*, 51–57.
